# Single-Crystal to Single-Crystal Addition of H_2_ to [Ir(^i^Pr-PONOP)(propene)][BAr^F^_4_] and Comparison Between Solid-State and Solution Reactivity

**DOI:** 10.1021/acs.organomet.2c00274

**Published:** 2022-07-26

**Authors:** Cameron G. Royle, Lia Sotorrios, Matthew R. Gyton, Claire N. Brodie, Arron L. Burnage, Samantha K. Furfari, Anna Marini, Mark R. Warren, Stuart A. Macgregor, Andrew S. Weller

**Affiliations:** † Department of Chemistry, University of York, Heslington YO10 5DD, York, U.K.; ‡ Department of Chemistry, University of Oxford, Mansfield Road, Oxford OX1 3TA, U.K.; § Institute of Chemical Sciences, Heriot-Watt University, Edinburgh EH14 4AS, U.K.; ∥ Diamond Light Source Ltd, Didcot OX11 0DE, U.K.; ⊥ Department of Chemistry, University of Southampton, Southampton SO17 1BJ, U.K.

## Abstract

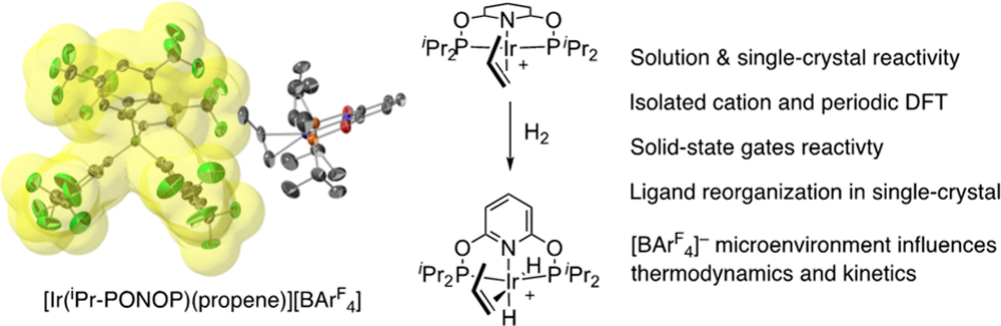

The reactivity of the Ir(I) PONOP pincer complex [Ir(^i^Pr-PONOP)(η^2^-propene)][BAr^F^_4_], **6**, [^i^Pr-PONOP = 2,6-(^i^Pr_2_PO)_2_C_6_H_3_N, Ar^F^ = 3,5-(CF_3_)_2_C_6_H_3_] was studied in solution and the solid state, both experimentally, using molecular density functional theory (DFT) and periodic-DFT computational methods, as well as in situ single-crystal to single-crystal (SC-SC) techniques. Complex **6** is synthesized in solution from sequential addition of H_2_ and propene, and then the application of vacuum, to [Ir(^i^Pr-PONOP)(η^2^-COD)][BAr^F^_4_], **1**, a reaction manifold that proceeds via the Ir(III) dihydrogen/dihydride complex [Ir(^i^Pr-PONOP)(H_2_)H_2_][BAr^F^_4_], **2**, and the Ir(III) dihydride propene complex [Ir(^i^Pr-PONOP)(η^2^-propene)H_2_][BAr^F^_4_], **7**, respectively. In solution (CD_2_Cl_2_) **6** undergoes rapid reaction with H_2_ to form dihydride **7** and then a slow (3 d) onward reaction to give dihydrogen/dihydride **2** and propane. DFT calculations on the molecular cation in solution support this slow, but productive, reaction, with a calculated barrier to rate-limiting propene migratory insertion of 24.8 kcal/mol. In the solid state single-crystals of **6** also form complex **7** on addition of H_2_ in an SC-SC reaction, but unlike in solution the onward reaction (i.e., insertion) does not occur, as confirmed by labeling studies using D_2_. The solid-state structure of **7** reveals that, on addition of H_2_ to **6**, the PONOP ligand moves by 90° within a cavity of [BAr^F^_4_]^−^ anions rather than the alkene moving. Periodic DFT calculations support the higher barrier to insertion in the solid state (Δ*G*^‡^ = 26.0 kcal/mol), demonstrating that the single-crystal environment gates onward reactivity compared to solution. H_2_ addition to **6** to form **7** is reversible in both solution and the solid state, but in the latter crystallinity is lost. A rare example of a sigma amine-borane pincer complex, [Ir(^i^Pr-PONOP)H_2_(η^1^-H_3_B·NMe_3_)][BAr^F^_4_], **5**, is also reported as part of these studies.

## 1 Introduction

**Scheme 1:**
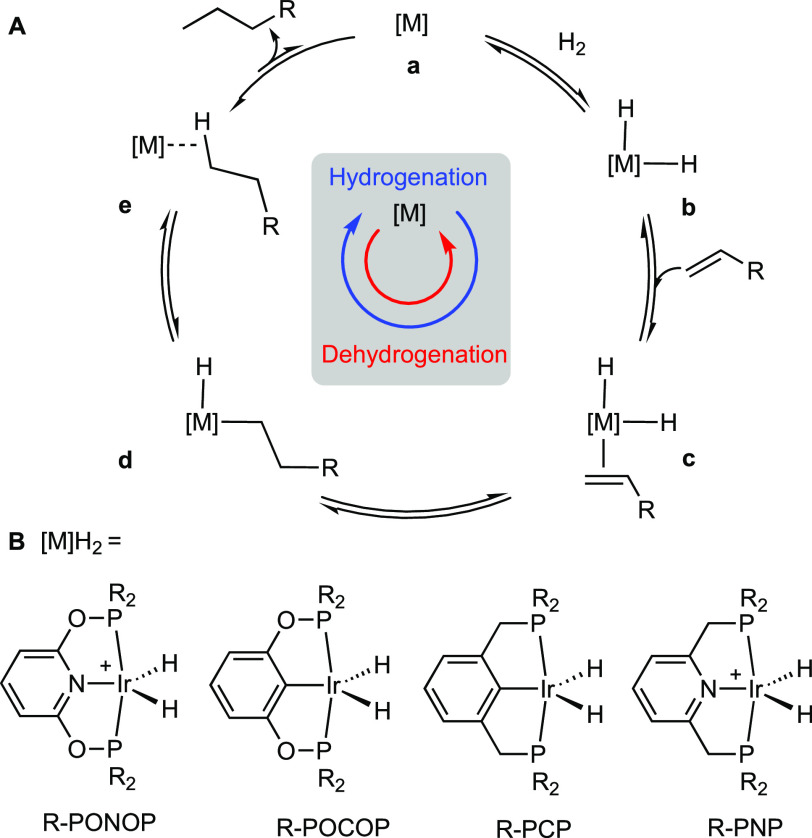
(A) Simplified Alkene Hydrogenation and DeHydrogenation Catalytic Cycles. (B) Exemplar Ir(pincer) Complexes R = Alkyl or Aryl Anions not shown.

The addition of dihydrogen and an alkene to metal centers, followed by migratory insertion and reductive elimination of an alkane, are elementary transformations in organometallic synthesis and catalysis.^1^ As well as being the key steps in catalytic alkene hydrogenation, the microscopic reverse of hydrogenation, alkane dehydrogenation, allows for the generation of simple alkenes from their corresponding alkanes,^2^ by C–H oxidative addition and β-elimination, Scheme 1A. Iridium complexes based upon the Ir(pincer)H_2_ motif (pincer = R-PCP, R-POCOP, or R-PONOP, R = alkyl or aryl, Scheme 1B) are often the systems of choice for alkane dehydrogenation reactions,^3,4^ operating by key, but yet to be experimentally observed, highly reactive Ir(pincer) intermediates such as 14-electron^5^ (**a**) or alkane σ-complexes^6,7^ (**e**). Under transfer dehydrogenation conditions intermediate **a** is generated from dihydride **b** using a sacrificial alkene in a complementary hydrogenation cycle.^8,9^

We have an interest in the generation and reactivity of σ-alkane complexes (i.e., **e**) using single-crystal to single-crystal (SC-SC^10,11^) solid-state molecular organometallic chemistry techniques (SMOM).^12^ For example, addition of H_2_ to crystalline [Rh(chelating-phosphine)(alkene)][BAr^F^_4_] precursors [Ar^F^ = 3,5-(CF_3_)_2_C_6_H_3_] results in the formation of the corresponding cationic alkane σ-complexes in a solid/gas SC-SC transformation by hydrogenation of the alkene to form an alkane, which remains bound to the metal center.^13−15^ In solution σ-alkane complexes are transient (lifetimes of μs to hrs), being observed using in situ spectroscopic methods, often at low temperatures.^16−18^ The stability of the σ-alkane complexes in the solid state comes from the [BAr^F^_4_]^−^ anions that provide a microenvironment of secondary noncovalent interactions organized in a tertiary periodic anionic motif.^15^ This stability also allows these well-defined σ-alkane complexes to be directly connected with the products of C–H activation, for example, in stoichiometric acceptorless alkane dehydrogenation at room temperature.^14,15^

We recently showed that this synthetic methodology can be extended to allow for the isolation of a cobalt σ-alkane complex.^19^ In contrast, the iridium analogue cannot be isolated, and a hydride-bridged dimer results.^20^ Inspired by the work of Brookhart and co-workers in exploring the solid/gas SC-SC reactivity of aryl-fluorinated (Ar^F^) pincer Ir(Ar^F^-POCOP)H_2_ complexes with alkenes and hydrogen in which hydride-bridging dimers do not form,^21^ and their reports of [M(^t^Bu-PONOP)(CH_4_)][BAr^F^_4_] complexes that are σ-alkane complexes (M = Rh^22^) or alkyl hydrides (M = Ir^23^), respectively, in solution at low temperature, we were interested in the SC-SC reactivity of cationic [Ir(PONOP)(propene)][BAr^F^_4_] complexes with H_2_ to explore if a propane^15^ (or propyl hydride) complex was accessible. A further motivation for this was the study of the elementary steps and intermediates of alkene hydrogenation/alkane dehydrogenation using Ir-pincer complexes by SC-SC reactivity. Propane to propene dehydrogenation is particularly interesting, as it is industrially relevant,^24^ and solid-phase supported Ir-pincer catalysts have used propene as a sacrificial acceptor in the gas-phase dehydrogenation of light alkanes^25^ as well as catalyzing direct propene hydrogenation.^26^

**Scheme 2:**
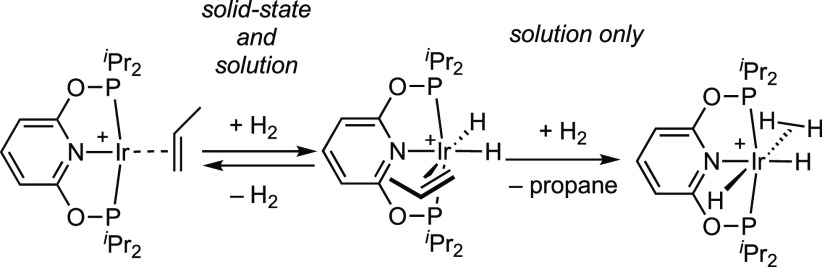
This Work [BAr^F^_4_]^−^ anions not shown.

In this contribution we report the synthesis of the new complex [Ir(^i^Pr-PONOP)(propene)][BAr^F^_4_] and compare its reactivity with H_2_ in solution and the solid state, Scheme 2. While this Ir(I) complex reversibly adds H_2_ in a solid-state SC-SC reaction that results in significant and unexpected reorientation of the ligand framework, onward reaction to form propane does not occur. By comparison, in solution the formation of the Ir(III) dihydrogen/dihydride complex [Ir(^i^Pr-PONOP)(H_2_)H_2_][BAr^F^_4_] results alongside the formation of propane, but with more mechanistic complexity than in the solid state. While, ultimately, a σ-propane complex was not accessible in the solid state, these observations reveal the role of the solid-state environment in mechanically gating organometallic reactivity. A rare example of a σ-bound amine-borane pincer complex is also reported as part of this study.

## 2 Results and Discussion

### 2.1 Solution Synthesis of [Ir(^i^Pr-PONOP)(H_2_)H_2_][BAr^F^_4_], [Ir(^i^Pr-PONOP)H_2_][BAr^F^_4_], and [Ir(^i^Pr-PONOP)H_2_(η^1^-H_3_B·NMe_3_)][BAr^F^_4_]

**Scheme 3:**
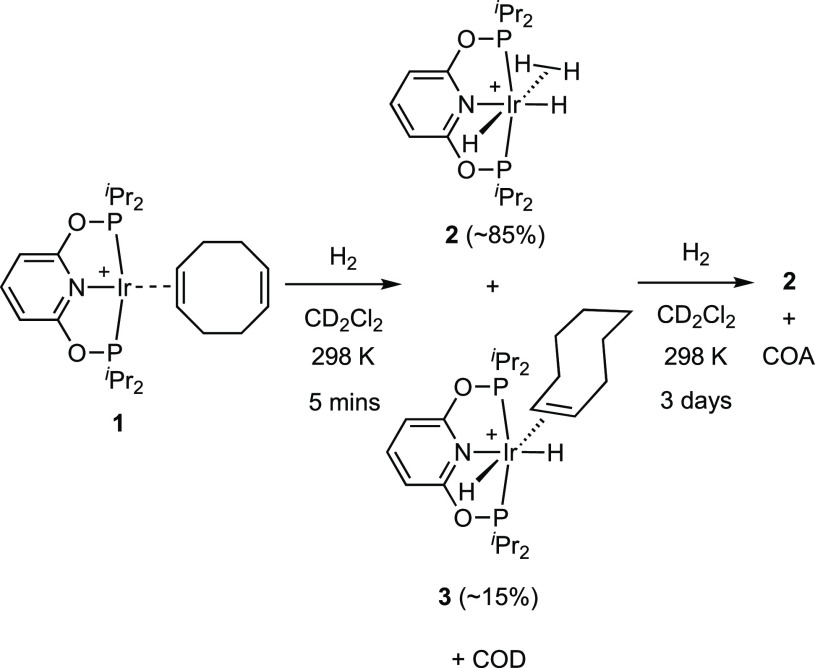
Synthesis of Complex 2 [BAr^F^_4_]^−^ anions are not shown.

A suitable entry point into the synthesis of the desired Ir(I) propene complex is an [Ir[(PONOP)H_2_]^+^ precursor, in the anticipation that reaction with excess propene would generate an Ir(I) propene-bound complex alongside free propane from hydrogenation.^27,28^ The precursor to this, [Ir(^i^Pr-PONOP)(η^2^-COD)][BAr^F^_4_]^29^**1**, was synthesized from addition of free ligand, ^i^Pr-PONOP, to [Ir(COD)_2_][BAr^F^_4_] (COD = cyclooctadiene, see the Supporting Information). In the ^1^H NMR spectrum of complex **1** signals due to bound [δ 4.45] and unbound [δ 5.68] alkene are observed, showing the COD does not reversibly dissociate or undergo intramolecular exchange on the NMR time scale. Resonances assigned to the ^i^Pr-PONOP ligand indicate overall *C*_2*v*_ symmetry for the cation, and consistent with this in the ^31^P{^1^H} NMR spectrum a single environment is observed.^30^ A simple rotation of the COD ligand would account for these data, as also suggested to occur for the Rh analogue [Rh(^i^Pr-PONOP)(η^2^-COD)][BAr^F^_4_].^31^ Addition of excess H_2_ (4 bar) to complex **1** in CD_2_Cl_2_ solution resulted in the rapid formation (5 min) of the dihydrogen/dihydride complex [Ir(^i^Pr-PONOP)(H_2_)H_2_][BAr^F^_4_], **2** (∼85%), alongside free COD and a complex identified as [Ir(^i^Pr-PONOP)H_2_(η^2^-COE)][BAr^F^_4_], **3** (∼15%), Scheme 3, (COE = cyclooctene). No free COE was observed. Complex **3** was characterized in the ^1^H NMR spectrum by the observation of two bound alkene [δ 3.00 and 2.93] and two inequivalent hydride environments [δ −10.41 and −17.77], while an AB doublet is observed in the ^31^P{^1^H} NMR spectrum that shows trans *J*(PP) coupling [280 Hz]. Given that COD does not dissociate rapidly in complex **1**, these observations are best explained by addition of H_2_ to form an unobserved dihydride complex [Ir(^i^Pr-PONOP)H_2_(η^2^-COD)][BAr^F^_4_] from which H_2_ and COE (the latter formed from slow hydrogenation of COD) must bind competitively compared with COD. Further hydrogenation of COE or COD to cyclooctane (COA) takes much longer (3 d, 4 bar H_2_), after which time only the pale yellow complex **2** and COA are observed.

Attempts to isolate complex **2** at the end of reaction by recrystallization led to the formation of intractable oils, while removal of the H_2_ atmosphere resulted in the reversible loss of bound H_2_ (vide infra). Characterization was thus performed in situ under a H_2_ atmosphere. Complex **2** has a highly fluxional manifold of hydride ligands. At 298 K a single slightly broadened resonance is observed in the ^1^H NMR spectrum, δ −8.79, that integrates to 4 H. This chemical shift is very similar to that reported for [Ir(^t^Bu-PONOP)H_4_][BAr^F^_4_], δ −8.9, which is also highly fluxional.^32^ Dissolved H_2_ is also observed as a broadened signal, suggesting a slow exchange occurs with **2** at room temperature. In the ^31^P{^1^σH} NMR spectrum a single environment is observed at δ 181.2. Spin-lattice relaxation time (*T*_1_) measurements^33^ (500 MHz) on the hydride signal showed that, at 295 K *T*_1_ = 119 ± 8 ms, while at 253 K *T*_1_ = 38 ± 1 ms. At the low temperature the hydride signal is now observed as a sharper triplet [δ −8.81, t, *J*(PH) = 7.5 Hz] and still relative integral 4 H. The fluxional behavior and the short *T*_1_ time at 253 K suggest rapidly interconverting isomers that sit on a rather flat potential energy surface, for which there is a significant contribution to the time-averaged structure from complexes with a dihydrogen ligand rather than an Ir(V) tetrahydride. Similar behavior has been studied in detail for Ir(^t^Bu-POCOP)H_4_ (*T*_1_ = 110 ms) and Ir(^t^Bu-PCP)H_4_ (*T*_1_ = 130 ms) but where a significant contribution from a Ir(V) tetrahydride is suggested.^34^

Replacing the H_2_ atmosphere over complex **2** with D_2_ resulted in the disappearance of the hydride signal at δ −8.79 in the ^1^H NMR spectrum and the observation of HD_(dissolved)_ at δ 4.55 [triplet, *J*(HD) = 43 Hz]. This confirms intermolecular exchange with D_2_ and also that subsequent intramolecular H/D exchange between the hydrides is occurring, either by a σ-CAM (σ-complex assisted metathesis) process or a Ir(V)-tetrahydride.^35−37^ In the ^2^H NMR spectrum a broad signal is observed at δ −8.8.

**Figure 1:**
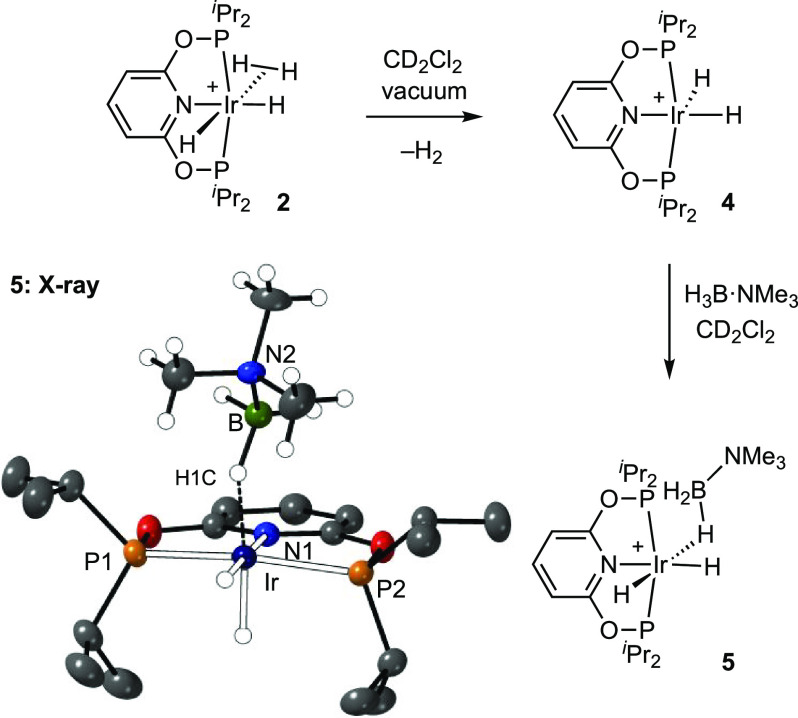
Synthesis of and solid-state structure of complex **5**. Displacement ellipsoids are shown at the 50% probability level. Selected bond lengths (Å) and angles (deg): Ir–P1, 2.2621(7); Ir–P2, 2.2660(6); Ir–N1, 2.103(2); Ir–B 2.771(4); Ir–H1C–B, 134(1). [BAr^F^_4_]^−^ anions are not shown.

In the absence of an H_2_ atmosphere complex **2** slowly (3 h) loses H_2_ in CD_2_Cl_2_ solution to form 16-electron [Ir(^i^Pr-PONOP)H_2_][BAr^F^_4_], **4**, Figure 1. This process can be accelerated by freeze/pump/thawing. In the resulting ^1^H NMR spectrum a single broad hydride environment is observed at δ −19.82, for which *T*_1_ measured at 295 K is fully consistent with a classical dihydride structure (1870 ± 10 ms).^33^ These data can be compared with those for the closely related dihydride [Ir(^t^Bu-PONOP)H_2_][BAr^F^_4_] (δ −25.07, *T*_1(min)_ = 873 ms).^37^

The behavior of the [Ir(^i^Pr-PONOP)H_4_]^+^ cation was also studied with density functional theory (DFT) calculations (see the Supporting Information for details) that identified [Ir(^i^Pr-PONOP)(η^2^-H_2_)(H)_2_]^+^ with *cis*-hydrides (i.e., Figure 1) as the most stable form. The alternative *trans* isomer (with η^2^-H_2_*trans* to N) corresponds to a transition state for intramolecular H/H(D) exchange that proceeds with a barrier of 11.1 kcal/mol. Dissociation of H_2_ from **2** entails a barrier of 9.6 kcal/mol and forms square-pyramidal **4** with an axial hydride, at +1.4 kcal/mol, consistent with facile intermolecular H_2_/D_2_ exchange but endergonic H_2_ loss that should still be possible upon application of a vacuum. Hydride exchange in **4** is computed to be extremely facile and proceeds via a *C*_2*v*_-symmetric dihydride isomer, **4′**, that lies only 2.9 kcal/mol above **4**, consistent with the symmetric structure suggested by ^1^H NMR spectroscopy.^38^

While we were unable to structurally characterize complex **4**, it reacts rapidly with H_3_B·NMe_3_ to quantitatively form an 18-electron σ-amine-borane complex, [Ir(^i^Pr-PONOP)H_2_(η^1^-H_3_B·NMe_3_)][BAr^F^_4_], **5**, Figure 1, for which small number of colorless cuboid crystals were obtained from a CD_2_Cl_2_/pentane recrystallization, allowing for a single-crystal X-ray diffraction study. The resulting solid-state structure shows a pseudo-octahedral Ir(III) complex, with two cis hydride ligands (located, but not freely refined), a PONOP ligand, and an η^1^-coordinated H_3_B·NMe_3_ ligand. The latter coordination mode is signaled by a long Ir···B distance [2.771(4) Å] and a rather open Ir–H–B angle [134(1)°].^13^ Structurally characterized complexes with η^1^-coordination modes of amine-boranes^39−42^ have been previously reported. The solution NMR spectroscopic data (CD_2_Cl_2_) are fully consistent with the solid-state structure. Two Ir–hydride environments are observed in the ^1^H NMR spectrum, at δ −20.01 (td) and −15.91 (td), and a broad, relative integral 3 H, signal at δ −2.20 is assigned to the Ir··H_3_B group that is undergoing rapid site exchange between terminal and bridging B–H groups.^42^ The ^11^B{^1^H} and ^31^P{^1^H} NMR spectra of complex **5** are unremarkable.

### 2.2 Synthesis of [Ir(^i^Pr-PONOP)(propene)][BAr^F^_4_] in Solution

**Scheme 4:**
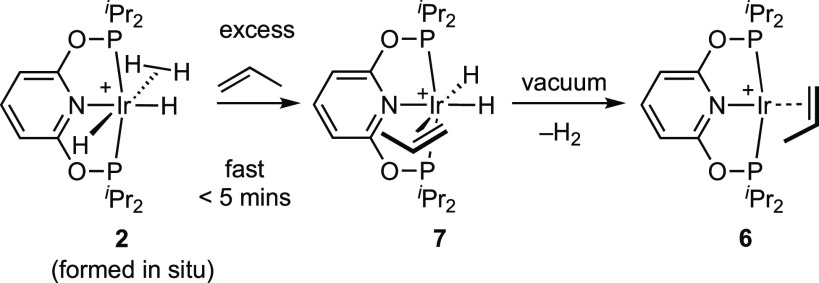
Solution Synthesis of Complex 6 [BAr^F^_4_]^−^ anions are not shown.

Addition of excess propene to in situ generated, pale yellow, complex **2** resulted in the immediate formation of the alkene dihydride complex [Ir(^i^Pr-PONOP)H_2_(η^2^-propene)][BAr^F^_4_], **7**, Scheme 4. While the onward reaction of **7** is slow (see Section 2.3 for characterization and reactivity), simply pumping a solution to dryness results in H_2_ loss and the formation of [Ir(^i^Pr-PONOP)(η^2^-propene)][BAr^F^_4_], **6**. Complex **6** thus formed can be isolated in crystalline form in 94% yield from a subsequent 1,2-difluorobenzene/pentane recrystallization. Analogous Ir(^t^Bu-PCP)(propene) and Ir(^t^Bu-POCOP) complexes are known.^43,44^

In CD_2_Cl_2_ solution a single set of resonances for the propene ligand are observed in the ^1^H and ^13^C{^1^H} NMR spectra of complex **6**, alongside four closely overlapping ^i^Pr methyl, two methine, and two pyridyl environments, the last in a 1:2 ratio. A single environment is observed in the ^31^P{^1^H} NMR spectrum, δ 188.6. These solution data point to a fluxional process for the propene that gives time-averaged *C*_2_ symmetry on the NMR time scale. This process is not frozen out at 183 K; that is, a single ^31^P environment is still observed. A simple rotation around the (C=C)–Ir–N vector accounts for this,^45^ as also suggested for complex **1**.

### 2.3 Addition of H_2_ to [Ir(^i^Pr-PONOP)(propene)][BAr^F^_4_] in Solution, and Characterization of [Ir(^i^Pr-PONOP)H_2_(propene)][BAr^F^_4_]

**Scheme 5:**
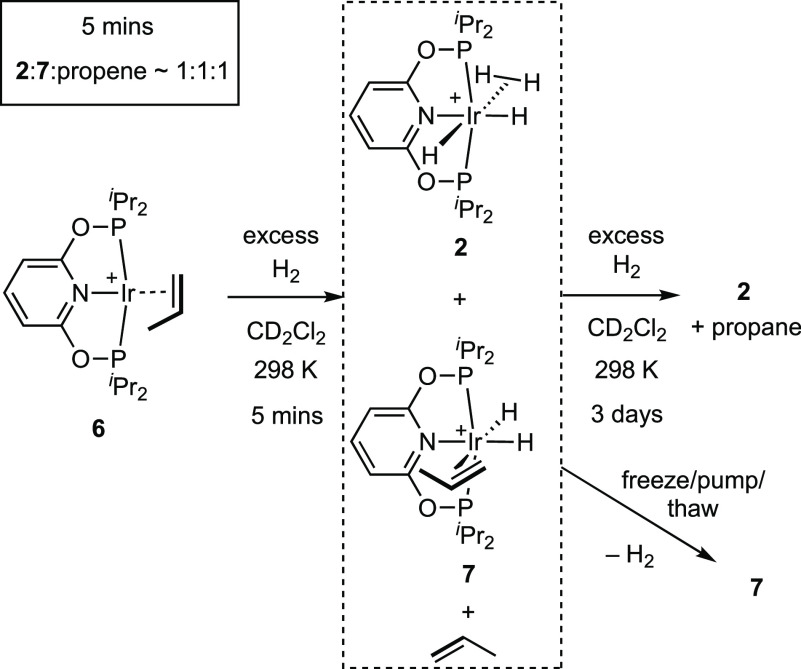
Reactivity of Complex 5 in CD_2_Cl_2_ Solution [BAr^F^_4_]^−^ anions not shown.

With the synthesis of complex **6** in hand its reactivity with H_2_ in CD_2_Cl_2_ solution was studied to determine baseline solution reactivity for comparison with the solid state. Addition of H_2_ (4 bar) to orange complex **6** rapidly (on time of mixing) resulted in the formation of a pale yellow solution that contains dihydride propene complex **7**, alongside dihydrogen/dihydride **2** and dissolved propene, all in approximately equal amounts, Scheme 5. Over the course of 3 d under H_2_ this mixture slowly turns to give complex **2** and propane as the only components. As for the COD complex **1** these observations are best accounted for by competitive binding of propene (i.e., complex **7**) and H_2_ (complex **2**). Evidence for an equilibrium between **2** and **7** is provided by the addition of H_2_ to a solution of complex **6**, shaking the NMR tube to ensure reaction with H_2_ to form **7**, and then removal of noncondensable gases (H_2_) from a frozen (77 K) solution under vacuum. Under these conditions of low H_2_ concentration but persistent propene, complex **7** is the sole organometallic product observed on thawing under Ar. This also allows for its definitive characterization free from other products.

In CD_2_Cl_2_ solution (Ar atmosphere, 298 K) the ^13^C{^1^H} NMR spectrum of complex **7** displays two alkene environments (δ 82.5, 62.0). In the ^1^H NMR spectrum sharp signals for two inequivalent Ir–H groups, at δ −11.64 and −16.50, that also show coupling to two ^31^P nuclei are observed alongside a single set of resonances due the bound propene. The ^i^Pr methyl groups resolve as at least five broad signals, and the methine signals are also complex. These data report on *cis*-hydride environments, with the propene in an axial coordination site. Two tightly coupled AB doublets are observed in the ^31^P{^1^H} NMR spectrum at δ 168.2 and 170.0 that show trans PP coupling [*J*(PP) = 320 Hz]. Inequivalent phosphine environments are expected for propene coordinated via a π-face in the axial position that is not undergoing reversible dissociation that is fast on the NMR time scale. At low temperature (185 K) the ^31^P{^1^H} NMR spectrum becomes significantly more complex, suggesting the resolution of two different isomeric species associated with the orientation of the propene ligand, which is undergoing fast rotation at room temperature. Two sets of hydride resonances are now observed at chemical shifts consistent with their frequency averaging at room temperature.

Under an Ar atmosphere in a sealed NMR tube complex **7** does not lose H_2_ in solution. However, as stated, pumping to dryness results in the reformation of **6**, showing that H_2_ addition is reversible, but its loss is likely an endergonic process. Over a 24 h period decomposition occurs in solution (∼25%) to unidentified products, and attempts to recrystallize **7** led to poorly diffracting crystalline material from which no structural solution was possible.

**Figure 2:**
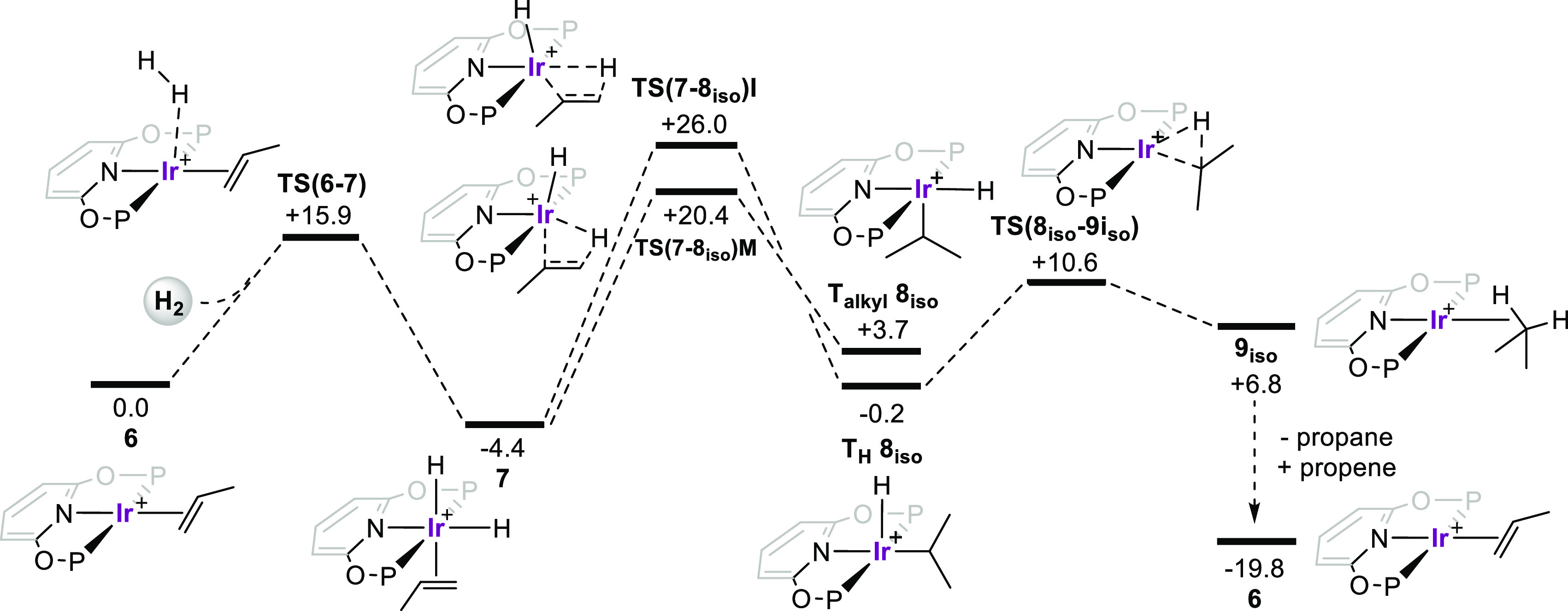
Computed free energy profile (kcal/mol) for propene hydrogenation from **6** modeled in dichloromethane solvent (*P* = P^i^Pr_2_; level of theory: BP86[D3BJ,CH_2_Cl_2_]/Def2TZVP//BP86/SDD (Rh, P, with polarization on P); 6-31G** on other atoms.

DFT calculations confirm the facile addition of H_2_ to **6** to form **7** (Δ*G*^‡^ = 15.9 kcal/mol; Δ*G*° = −4.4 kcal/mol, Figure 2) and that this reaction, with a return barrier of 20.3 kcal/mol, should be reversible if H_2_ is removed from the system. In contrast, the onward hydrogenation of propene from **7** is much less accessible due to the significant barriers associated with the propene migratory insertion step. The lowest-energy process is shown in Figure 2 and has an overall barrier of 24.8 kcal/mol via **TS(7–8**_**iso**_**)M**, consistent with a slow process at room temperature. **TS(7–8**_**iso**_**)M** leads to an isopropyl intermediate, **8**_**iso**_, and geometrically this step is best described as a hydride migration onto the propene ligand (labeled “M”) that is coupled to movement of the spectator hydride such that the isopropyl ligand occupies the axial site in **8**_**iso**_ as the T_alkyl_ isomer. A second pathway where propene inserts (labeled “I”) into the adjacent Ir–H bond proceeds via **TS(7–8**_**iso**_**)I** at +26.0 kcal/mol and leads to the more stable T_H_ isomer of **8**_**iso**_ at −0.2 kcal/mol. Hydride migration and propene insertion transition states to form the *n*-propyl species (**8**_**n**_) were also located (**TS(7–8**_**n**_**)M**: +21.9 kcal/mol; **TS(7–8**_**n**_**)I**: 24.7 kcal/mol) and gave the T_alkyl_ and T_H_ isomers of **8**_**n**_ at +4.3 kcal/mol and −3.1 kcal/mol, respectively (see the Supporting Information). All these migratory insertion transition states exhibit “late” geometries; for example, in **TS(7–8**_**iso**_**)M** the forming C–H and Ir–C_alkyl_ bonds (1.21 Å/2.19 Å) are already close to their computed values in **T**_**alkyl**_**8**_**iso**_ (1.10 Å/2.15 Å). The high barriers to insertion are likely linked to the formation of a strongly donating alkyl ligand trans to the spectator hydride, although movement of the spectator hydride ligands does serve to mitigate this effect (the H–Ir–C_alkyl_ angle is ca. 140° in all cases).

Once formed, **T**_**alkyl**_**8**_**iso**_ must first isomerize to **T**_**H**_**8**_**iso**_ in order to access C–H reductive coupling via **TS(8**_**iso**_**-9**_**iso**_**)** (+10.6 kcal/mol) to give the propane complex **9**_**iso**_ (+6.8 kcal/mol). The reaction is then driven to completion by displacement of propane by either propene (Δ*G*° = −19.8 kcal/mol relative to **6**) or (if present) H_2_ to give **2** (Δ*G*° = −14.7 kcal/mol). The calculations thus indicate the equilibrium between **2** (+ propene) and **7** (+ H_2_) will favor the latter (Δ*G* = −5.1 kcal/mol) while **2** is still accessible under H_2_ pressure.

### 2.4 SC-SC Reactivity in the Solid State

In contrast to the solution behavior, addition of H_2_ to finely crushed crystalline samples of complex **6** (∼50 mg, 15 min, 4 bar) did not result in the formation of tetrahydride complex **2** or the hydrogenation of propene to propane, even after 5 d under H_2_. Instead room-temperature ^31^P{^1^H}/^13^C{^1^H} solid-state NMR (SSNMR) and solution (CD_2_Cl_2_) spectroscopy of dissolved crystalline material showed the formation of the dihydride alkene complex [Ir(^i^Pr-PONOP)H_2_(η^2^-propene)][BAr^F^_4_], **7**, with data identical to those obtained by solution methods. Under a system open to Ar, in the solid-state complex **7** loses H_2_ to reform **6**, a process that is speeded up by application of a dynamic vacuum (2 h). No decomposition is observed, unlike in solution. Because of the experimental challenges of keeping samples under a H_2_ atmosphere when manipulating in the solid state, these samples show 90% complex **7** with the remainder being complex **6**. Analysis of single crystals of complex **7** hydrogenated ex situ in the bulk, by rapid transfer to the diffractometer, leads to a structural refinement in which the electron density due to the alkene ligand could not be satisfactorily modeled, a consequence of partial H_2_ loss to reform complex **6**. Analysis of material that had completely reformed complex **6** after H_2_ loss showed only weak Bragg peaks and showed evidence of significant crystal cracking (scanning electron microscopy (SEM), Supporting Information). As the resulting ^31^P{^1^H} SSNMR spectrum of this material was indistinguishable from initially synthesized complex **6**, this suggests retention of short-range order but loss of bulk crystallinity.^46^ The formation of microcrystalline, or amorphous, products as a response to solid-state reactivity in single crystals is well-documented.^47−50^

**Figure 3:**
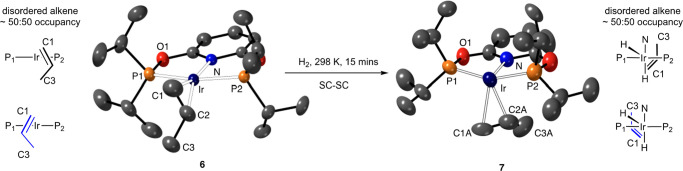
Solid-state structures of the cations in complexes **6** and **7** (from an SC-SC reaction) as determined by single-crystal X-ray diffraction (273 K). [BAr^F^_4_]^−^ anions and hydrogen atoms are not shown; displacement ellipsoids are shown at the 30% probability level. Hydrides were not located in **7**. Only one disordered component of the propene is shown in each case. Selected bond distances (Å) and angles (deg): **Complex 6** Ir–C1, 2.138(5); Ir–C2, 2.209(5); Ir–P1, 2.2603(7); Ir–P2, 2.2635(7); Ir–N, 2.044(2); C1–C2, 1.271(8); C2–C3 1.56(1); ∠Ir–C1–C2/P1–P2–N–Ir, 66.7(4). **Complex 7** Ir–C1A, 2.36(6); Ir–C2A, 2.25(5); Ir–P1, 2.26(1); Ir–P2, 2.26(1); Ir–N, 2.12(2); C1A–C2A, 1.31(8); C2–C3, 1.46(6); ∠C1–C2/N–Ir, 48(3)°.

To circumvent this problem of H_2_ loss in complex **7** we used in situ SC-SC techniques on the I19 Beamline at the Diamond Light Source for its synthesis from **6**. This allowed for the collection of data under an atmosphere of H_2_ and thus the formation of complex **7** without reforming **6**. The solid-state structure of complex **6** is shown in Figure 2. Data were collected at 273 K allowing for the subsequent reaction with H_2_ to be followed crystallographically in situ. The structural solution is good [*R*(2σ) = 4.0%, *R*_int_ = 4.3%, *P*1̅ space group] and shows a pseudo-square-planar cation, as expected for an Ir(I) complex. The propene is disordered essentially equally between two sites, related to one another by a noncrystallographic mirror plane (inset left Figure 2 and Figure 3), accounted for by coordination of a different π-face of propene in each. The C–C distances in the propene are consistent with a double and a single bond [1.271(8) and 1.56(1) Å, respectively]. The Ir···C distances show alkene coordination with a non-interacting methyl group: Ir–C1, 2.138(5); Ir–C2; 2.209(5); Ir–C3, 3.15(5) Å. Data collection at 150 K on a laboratory-based diffractometer (Cu source), while more precise [*R*(2σ) = 1.9%, *R*_int_ = 2.0%], showed the same disorder indicating that there are no dynamic processes operating in the solid state that involve reorientation of the propene.^51^

The supporting [BAr^F^_4_]^−^ anions present a skewed-bicapped square prism motif, in which 10 anions surround two crystallographically identical cations.^52^ This is discussed in more detail in Section 2.6.

**Figure 4:**
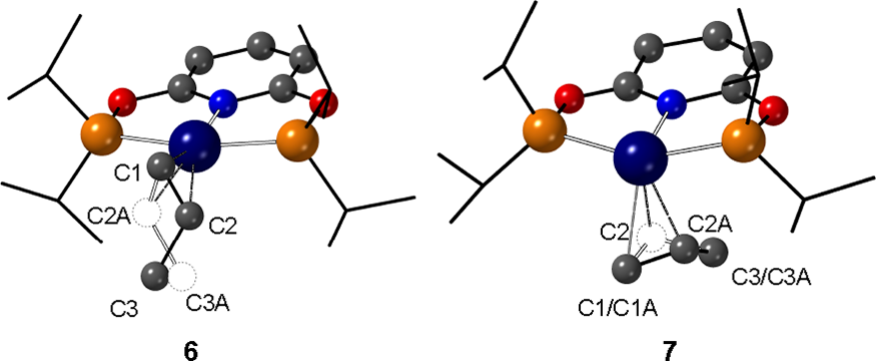
Comparison of structures of complexes **6** and **7** orientated to compare the propene coordination and disorder. [BAr^F^_4_]^−^ anions are not shown.

Addition of H_2_ (2 bar) to the *same* single crystal used for the structural analysis of complex **6** at 273 K led to the formation of complex **7**, for which a satisfactory structural solution could be determined, Figure 3 [*R*(2σ) = 9.3%, *R*_int_ = 8.7%, *P*1̅ space group]. However, there was a significant deterioration in data quality on hydrogenation resulting in a drop in resolution from 0.65 Å in **6** to 1.2 Å for **7**. Complex **7** has a pseudo-octahedral coordination geometry, in which the propene has moved from the equatorial position found in **6** to an axial position. The remaining coordination sites are occupied by the hydrides, which were not located, but their presence is confirmed by solution NMR spectroscopy on dissolved crystals. In this SC-SC transformation the disorder observed in the propene ligand in **6** is retained in complex **7**, with two orientations observed, in an ∼50:50 ratio, Figures 3 and 4. Because of the lower quality of data the C–C and C=C distances in the propene were restrained to sensible distances [1.46(6), 1.31(8) Å, respectively], and detailed discussion of the metrics of propene binding are not appropriate. Confidence in the assignment of C=C and C–C bonds comes from the orientation of the disordered propene fragments that map onto those observed in starting **6**, the longer Ir···C distance to the third propene carbon atom [Ir–C3A, 3.29(4) Å], and solid-state NMR data that support the formation of two isomers, as discussed next.

### 2.5 Solid-State NMR Analysis of Complexes **6** and **7**

In the ^31^P{^1^H} SSNMR spectrum of complex **6** two ^31^P environments are observed [δ 189.5, 194.1], as an AB doublet showing large ^31^P–^31^P coupling, consistent with trans-disposed, crystallographically distinct, phosphines, *J*(PP) = 310 Hz.^53^ While the two, noncrystallographically generated, disordered components observed in the solid-state structure suggest there should be four ^31^P environments, we suggest a coincidence of signals results in only two being observed. In contrast, in the ^13^C{^1^H} NMR spectrum four signals are observed in the bound alkene region [δ 57.4, 56.6, 44.8, 42.9], fully consistent with the solid-state structure. In the ^31^P{^1^H} SSNMR spectrum of complex **7** a complex set of overlapping resonances is observed, centered around δ 174,^54^ while in the ^13^C{^1^H} SSNMR spectrum two sets of alkene signals are observed. These data are consistent with two isomers being formed for complex **7** in the solid state, leading on from the two isomers observed for propene binding in complex **6** that are related by the relative orientation of the methyl group.

### 2.6 Movement of the PONOP-Ligand on the SC-SC Transformation

**Figure 5:**
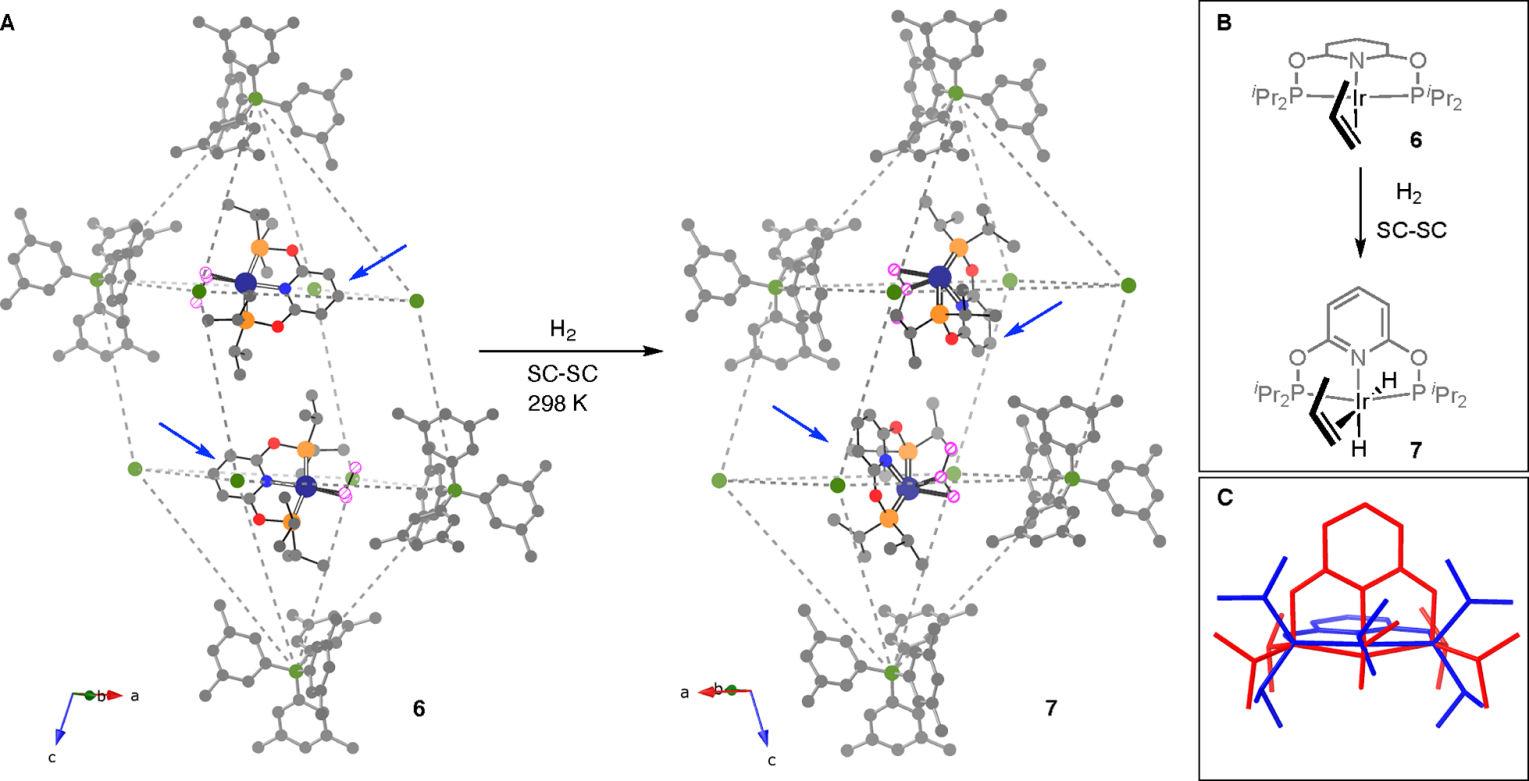
(A) Arrangement of anions and cations for complexes **6** and **7**. The anion motif is described by B atoms of the [BAr^F^_4_]^−^ (with selected anions shown, without F atoms), and enclosed cations are depicted in ball-stick format. The propene ligand in each is highlighted as a hatched circle, and only one disordered component is shown (see Figure 3). Arrows indicate the PONOP pyridyl ligand that moves on H_2_ addition. (B) Diagram of **6** and **7** orientated with regard to propene, showing the movement of the PONOP ligand. (C) Overlaid **6** (blue) and **7** (red) stick representations in the same orientation as shown in (B).

Oxidative addition of H_2_ to complex **6** results in the alkene in **7** now being orientated orthogonal to its starting position, Figures 3 and 4. We initially interpreted this as coming from movement of the alkene ligand around the coordination sphere of the iridium in the SC-SC transformation. However, inspection of the wider crystalline environment of [BAr^F^_4_]^−^ anions surrounding the cationic metal center shows that, surprisingly, it is the ^i^Pr-PONOP ligand that moves on oxidative addition of H_2_, not the alkene. Figure 5A shows that the [BAr^F^_4_]^−^ anions in **6** form a skewed bicapped square prism (BCSP) that surrounds two crystallographically related [Ir(^i^Pr-PONOP)H_2_(η^2^-propene)]^+^ cations. BCSP motifs have been reported previously in SMOM chemistry.^55,56^ The cations are orientated so that the propene is directed into a pocket formed by three of the [BAr^F^_4_]^−^ aryl groups of a single anion, with the pyridyl group directly trans to the propene. On addition of H_2_ in the SC-SC transformation the skewed BCSP motif is retained (difference in unit cell volumes = 1.9%), but there is a small contra-translation of the [BAr^F^_4_]^−^ anions in upper and lower basal planes. The orientation of the propene is also retained in the same pocket of aryl groups. What has changed significantly is the orientation of the PONOP ligand, which has pivoted ∼90° around the P–Ir–P vector so that it now sits orthogonal to the propene. Figure 5B,C shows this schematically and by an overlay of the crystallographically determined structures, respectively. While such large movements of metal–ligand fragments on SC-SC transformations are precedented, they are rare.^57−59^ In the case here, this reorientation may explain why the crystal quality degrades significantly on cycling H_2_ addition/removal, that is, **6**-**7**-**6**.

The addition of H_2_ to **6** to form **7** also results in a change in the oxidation state of the metal, accompanied by a move from pseudo square planar to octahedral. SC-SC transformations that involve an overall change in oxidation state have been reported previously,^10^ for example, Mn(II)/Mn(IV);^60^ Co(II)/Co(III);^61^ Rh(I)/Rh(III);^13,62,63^ Ir(I)/Ir(III);^21^ Ir(III)/Ir(V);^64^ and Au(I)/Au(III).^65^

### 2.7 Differences between Solution and Solid-State Reactivity

That the dihydrogen/dihydride complex **2** is formed in solution on hydrogenation of complex **6** while only complex **7** formed in the solid-state could arise from two restraining conditions imposed by the crystalline microenvironment of [BAr^F^_4_]^−^ anions. First, the reversible dissociation of propene from complex **7** that occurs in solution on addition of H_2_ is clearly disfavored in the solid state. Second, onward reactivity of the propene hydride to ultimately form free propane, by migratory insertion and reductive elimination, and **2** could be disfavored due to local steric effects of the proximal [BAr^F^_4_]^−^ anion that hinder either of these transformations. In both solid state and solution complex **7** undergoes H_2_ loss to reform **6**. We thus turned to computational studies in the extended solid state to understand these fundamental steps in greater detail and compare with solution DFT studies on the molecular cations reported above.

### 2.8 Computational Studies in the Solid State

**Figure 6:**
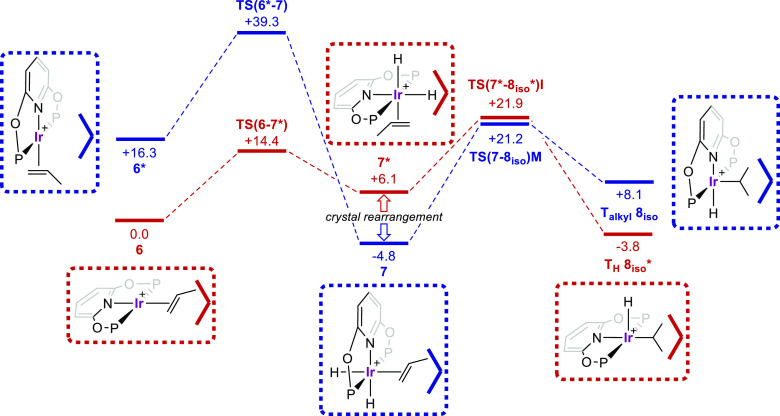
Computed free energy profiles (kcal/mol) for reversible H_2_ addition to **6** and propene migratory insertion in **7** (P = P^i^Pr_2_). Data are computed either in the unit cell of **6** (red) or **7** (blue). The heavy open V indicates the relative position of the adjacent BAr^F^_4_^–^ anion. Level of theory: periodic-DFT: PBE-D3/DZVP-MOLOPT-SR-GTH/GTH-PBE (plane wave cutoff = 500 Ry).

The structures of **6** and **7** in the extended solid state were computed with periodic DFT calculations using the PBE-D3 functional. Geometries for both species were based on the experimental crystal structures, and subsequent reactivity studies considered changes at one of the two Ir centers present in each unit cell (see the Supporting Information for full details). Key stationary points are presented in Figure 6, where the profile in red is computed using the unit cell of **6**, while that in blue uses the unit cell of **7**.

H_2_ addition to **6** to form **7** has a computed free energy change of −4.8 kcal/mol, indicating that, while **7** is favored thermodynamically, **6** may still be accessible from **7** under conditions of H_2_ loss. Kinetically, however, H_2_ loss directly from **7** is inaccessible with a barrier of 44.1 kcal/mol; the extended crystal environment within **7** therefore blocks this process. In contrast, H_2_ oxidative addition to **6** is feasible (Δ*G*^‡^ = +14.4 kcal/mol) and forms a propene dihydride complex within the crystal environment of **6**. This species, 7*, is 10.9 kcal/mol less stable than **7**, and we propose that one reason for this arises from the movement of the propene ligand to outside the pocket defined by the adjacent [BAr^F^_4_]^−^ anion (represented by the open “V” in Figure 6) that occurs upon H_2_ addition. On the basis of the experimentally observed, reversible H_2_ addition to **6** to give **7**, we speculate that the reorganization of the crystal lattice (that occurs with the net movement of the pincer ligand relative to the anionic framework) must occur at 7* to move between the “red” (7*) and “blue” (**7**) crystal lattices.^66^ Viewed in reverse, and assuming facile rearrangement between the two crystal lattices, the loss of H_2_ from **7** can occur with an overall barrier of 19.2 kcal/mol via TS(6–7*).

The positioning of the propene relative to the [BAr^F^_4_]^−^ anion pocket appears to be a key factor in determining the energy of other stationary points in these systems. Thus, in 6* the propene sits outside the pocket, and this structure is 16.3 kcal/mol higher than that of **6**. Similarly, H_2_ reductive elimination from **7** is also strongly disfavored, as this requires the propene to move out of the pocket in order to restore square-planar coordination at the Ir(I) center in 6*. The preferred pathways for propene migratory insertion are also impacted by the crystal environment. Thus, for **7** the hydride migration pathway is favored via **TS(7–8**_**iso**_**)M** at +21.2 kcal/mol, as this allows the propene to remain in the anion pocket. For 7* alkene insertion into the hydride bond via TS(7*-8_iso_*)I at +21.9 kcal/mol is preferred, as this permits the propene to remain within the pocket. In contrast, propene insertion in **7** and hydride migration in 7* have barriers in excess of 40 kcal/mol as they necessitate movement of propene out of the anion pocket (see the Supporting Information). This demonstrates a significant impact of the solid-state environment: in solution the barriers to the four different migratory insertion steps spanned only 5.6 kcal/mol, whereas in the solid state the range is over 30 kcal/mol.

The barrier to propene migratory insertion in **7** in the solid state is 26.0 kcal/mol, slightly higher than that of 24.8 kcal/mol computed in solution. Thus, the slow hydrogenation of propene that does occur in solution is somewhat more disfavored in the solid state. Experimentally, formation of propane is not observed in the solid state.^67^ The possibility of reversible migratory insertion in the solid state was probed by addition of D_2_ (15 min) to finely crushed **6**. This resulted in the formation of [Ir(^i^Pr-PONOP)D_2_(propene)][BAr^F^_4_], **d**_**2**_**-7**, as indicted by a featureless high-field region of the ^1^H NMR spectrum of dissolved material, and two Ir–D signals observed in the ^2^H NMR spectrum at chemical shifts essentially the same as for **7**. Longer reactions times (3 d) did not result in H/D exchange into the bound propene as measured by ^1^H and ^2^H NMR spectroscopy, consistent with the significant computed barrier to migratory insertion. Reversible alkene insertion in Ir(pincer)(alkene)D_2_ complexes is known, as determined by H/D exchange experiments into the bound alkene.^28^ Interestingly there is an example of insertion being reversible, but no H/D exchange is observed.^27^ DFT calculations suggest that this is due to a high barrier for ring opening of the insertion product, which has an agostic alkyl group. Such a scenario is not happening in our systems.

## 3 Conclusions

While still relatively uncommon, organometallic synthesis and reactivity in the single-crystalline environment is becoming an increasingly appreciated methodology.^10,11^ However, examples where solution and crystalline-phase reactivity can be directly compared experimentally^68^ and computationally are rare, in part a consequence of the stabilizing role that the solid state has on reactive complexes that are challenging to access in solution (e.g., σ-alkane complexes^6^). With [Ir(^i^Pr-PONOP)(η^2^-propene)][BAr^F^_4_] we have such a platform through reactivity with H_2_ to initially form [Ir(^i^Pr-PONOP)(η^2^-propene)H_2_][BAr^F^_4_]. Subsequent migratory insertion and reductive elimination of propane occurs in solution but in the solid state does not. While the solution-phase chemistry is more complex than that observed in the SC-SC transformation, with equilibria operating through reversible alkene dissociations that are not available in the solid state, the solid-state crystalline microenvironment, and, in particular, the positioning of the propene ligand relative to the [BAr^F^_4_]^−^ aryl groups, is a key factor in determining both the relative (higher) barrier to migratory insertion and the observed structures. The significantly more stable arrangement when propene sits in the pocket formed by the aryl groups in the crystalline lattice leads to both ^i^Pr-PONOP ligand movement and plasticity in the anion lattice to accommodate this motif on reaction of **6** with H_2_ to form **7**. While this reorganization provides access to the most stable structures in the solid state, a consequence is that the barriers to onward reactivity (e.g., migratory insertion) are significant, meaning that the targeted complex that has propane interacting with the Ir center is not accessible via this route. What will be interesting to explore is if engineering both the anion-motif and metal–ligand fragment in related SMOM systems allows for a reduction in these barriers through ground-state destabilization and/or transition-state stabilization.^69^ These are concepts that are familiar in enzyme catalysis, where the primary, secondary, and tertiary structures also work in concert to lower barriers to elementary reaction steps.^70^

## Supporting Information Available

Full experimental details, synthesis of new complexes, spectroscopic data, details of in situ X-ray diffraction experiments, and computational studies (PDF)
Cartesian coordinates of the DFT calculated structures (XYZ)

## Supplementary Material

om2c00274_si_001.pdf

om2c00274_si_002.xyz

## Dedication

Dedicated to Professor Maurice Brookhart (Brook) for his immense contributions to organometallic synthesis and catalysis, and his generosity and support of others in the field.
