# Supplementary material for: Single-Crystal to Single-Crystal Addition of H2 to [Ir(iPr-PONOP)(propene)][BArF4] and Comparison Between Solid-State and Solution Reactivity
Source: Organometallics. 2022 Jul 26;41(22):3270–80. doi: 10.1021/acs.organomet.2c00274 (PMC9709992; doi:10.1021/acs.organomet.2c00274)
Supplement: Supplementary file 1 — om2c00274_si_001.pdf [file om2c00274_si_001.pdf]

Electronic Supporting Information (ESI)

Single-Crystal to Single-Crystal Addition of H_2 to [Ir(^{i}Pr-PONOP)(propene)][BAr^F$_4$] and Comparison Between Solid-State and Solution Reactivity.

Cameron G. Royle,[a,b] Lia Sotorrios,[c] Matthew R. Gyton,[a] Claire N. Brodie,[a] Arron L. Burnage,[c] Samantha K. Furfari,[a] Anna Marini,[d,e] Mark R. Warren,[d] Stuart A. Macgregor,[c]* Andrew S. Weller[a]*

[a] Department of Chemistry, University of York, Heslington, York, YO105DD (U.K.). [b] Department of Chemistry, University of Oxford, Mansfield Road, Oxford, OX1 3TA (UK). [c] Institute of Chemical Sciences, Heriot-Watt University, Edinburgh, EH14 4AS (U.K.). [d] Diamond Light Source Ltd., Didcot OX11 0DE (U.K.); [e] Department of Chemistry, University of Southampton, Southampton SO17 1BJ (U.K.).

Table of Contents

1. Experimental 2

1.1. General Procedures 2

1.2. Synthesis and Characterisation 3

1.2.1. Synthesis of [(^{i}Pr-PONOP)Ir(η^2-COD)][BAr^F$_4$] (1) 3

1.2.2. Synthesis of [(^{i}Pr-PONOP)IrH$_2$(H$_2$)][BAr^F$_4$] (2) 6

1.2.3. Synthesis of [(^{i}Pr-PONOP)IrH$_2$][BAr^F$_4$] (4) 10

1.2.4. Synthesis of [(^{i}Pr-PONOP)IrH$_2$(σ-H$_3$BNMe$_3$)][BAr^F$_4$] (5) 12

1.2.5. Synthesis of [(^{i}Pr-PONOP)Ir(η^2-propene)][BAr^F$_4$] (6) 14

1.2.6. Synthesis of [(^{i}Pr-PONOP)IrH$_2$(η^2-propene)][BAr^F$_4$] (7) 18

1.3. Crystallography 26

1.4. SEM Imaging 30

2. Computational details 31

2.1. Solid-state calculations 31

2.2. Molecular calculations 32

2.3. Additional free energy diagrams 32

3. References 35

1. Experimental

1.1. General Procedures

All manipulations, unless otherwise stated, were performed under an argon atmosphere using standard Schlenk line and glove-box techniques. Glassware was oven-dried at 130 °C overnight and flamed under vacuum prior to use. CH_2Cl_2 and pentane were dried using a Grubbs-type solvent purification system (Innovative Technologies) and degassed by three successive freeze-pump-thaw (FPT) cycles. CD_2Cl_2 and 1,2-$C_6H_4F_2$ (pre-treated with alumina) were dried over CaH_2, vacuum distilled, degassed (3 × FPT) and stored over 3 Å molecular sieves. 1,5-Cyclooctadiene (COD) was dried over sodium and fractionally distilled before use and stored over 3 Å molecular sieves. $H_3B{\cdot}NMe_3$ was recrystallised from diethyl ether before use. Na[BAr^F_4],[1] [Ir(COD)$_2$][BAr^F_4][2] and iPr-PONOP[3] were prepared by literature methods.

Solution state NMR spectra were recorded on Bruker Avance III HD 500 MHz or 600 MHz NMR spectrometers at 298 K unless otherwise specified. Residual protio solvent was used as reference for ^1H spectra in deuterated solvent samples.[4] $^{31}P\{^1H\}$ NMR spectra were externally referenced to 85% H_3PO_4. ^1H assignments were aided by $^1H\{^{31}P\}$ experiments. All chemical shifts (δ) are quoted in ppm and coupling constants (*J*) in Hz. NMR assignments were aided by 2D spectra (^1H,^1H-COSY, ^1H,^{13}C-HSQC, ^1H,^{13}C-HMBC, ^1H,^1H-NOESY) where required.

Solid state NMR spectra were recorded on a wide bore Bruker Avance III HD spectrometer (^{13}C = 100.66 MHz; ^{31}P = 162.03 MHz) in 4.0 mm zirconia rotors with a MAS rate of 10 kHz unless otherwise specified. Rotors were packed and sealed in an argon filled glovebox. Spectra are referenced externally to $SiMe_4$ or H_3PO_4 using the secondary references adamantane (^{13}C δ = 29.5 for the shielded methylene resonance)[5] or triphenylphosphine (^{31}P, δ = -9.3)[6]

Elemental microanalyses were performed by Dr Graeme McAllister on an Exeter Analytical Inc. CE-440 at the University of York.

Electrospray Ionisation Mass Spectrometry (ESI-MS) was carried out using a Bruker compact® Time of Flight mass spectrometer by Mr Karl Heaton at the University of York.

1.2. Synthesis and Characterisation

1.2.1. Synthesis of [(^{i}Pr-PONOP)Ir(η^2-COD)][BAr$^F_4$] (1)

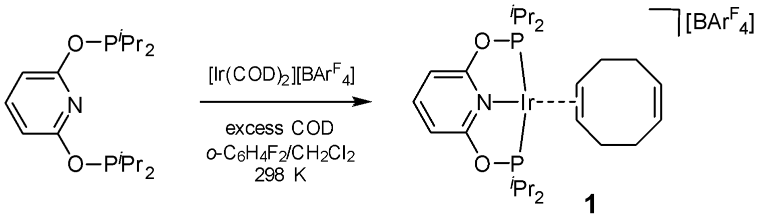

COD (50 μL, 4.1 x 10^1 μmol) was added to a solution of [Ir(η^2,η^2-COD)$_2$][BAr$^F_4$] (100 mg, 78.6 μmol) in CH_2Cl_2 (2 mL). A solution of ^{i}Pr-PONOP (27 mg, 79 μmol) in 1,2-difluorobenzene (0.2 mL) was added dropwise to the stirred solution of [Ir(η^2,η^2-COD)$_2$][BAr$^F_4$] at 298 K, inducing a colour change from rusty pink to bright orange. The mixture was stirred for an additional 60 minutes before transfer to an appropriate crystallisation vessel and layering with pentane at 298 K to give [(^{i}Pr-PONOP)Ir(η^2-COD)][BAr$^F_4$] (**1**; 107 mg, 90%) as large orange-red crystals amenable to *sc*-XRD studies after two weeks.

^{1}H NMR (400 MHz, CD_2Cl_2, 298 K) δ: 7.93 (t, $^3J_{HH}$ = 8.2 Hz, 1 H; heterocyclic C4), 7.73 (br. m, 8 H; [BAr$^F_4$]$^-$ *o*-H), 7.57 (s, 4 H; [BAr$^F_4$]$^-$ *p*-H), 6.90 (d, $^3J_{HH}$ = 8.2 Hz, 2 H; heterocyclic C3,5), 5.68 (t, $^3J_{HH}$ = 3.7 Hz, 2 H; uncoordinated alkene CH), 4.45 (br. m, 2 H; bound alkene CH), 2.56 (septet, $^3J_{HH}$ = 7.2 Hz, 4 H; ^{i}Pr CH), 2.49 (m, 4 H; COD Ir-C$_{sp2}$H-C$_{sp3}$$H_2$ + Ir-C$_{sp2}$H-C$_{sp3}$H$_2$-C$_{sp3}$$H_2$), 2.20 (m, 2 H; COD Ir-C$_{sp2}$H-C$_{sp3}$H$_2$-C$_{sp3}$$H_2$), 1.89 (m, 2 H; COD Ir-C$_{sp2}$H-C$_{sp3}$$H_2$), 1.26 (m, 24 H; ^{i}Pr CH$_3$).

^{13}C{^{1}H} NMR (102 MHz, CD_2Cl_2, 298 K) δ: 164.5 (vt, $^2J_{CP}$ = 2.8 Hz; heterocyclic C2,6), 162.2 (q, $^1J_{CB}$ = 49.8 Hz (^{11}B); [BAr$^F_4$]$^-$ *i*-C$_{aryl}$), 146.3 (s; heterocyclic C4), 135.2 (s; [BAr$^F_4$]$^-$ *o*-C$_{aryl}$), 130.0 (s; spectator alkene CH), 129.3 (qq, $^2J_{CF}$ = 31.5 Hz, $^4J_{CF}$ = 2.9 Hz; [BAr$^F_4$]$^-$ C$_{aryl}$-CF$_3$), 125.0 (q, $^1J_{CF}$ = 272.6 Hz; [BAr$^F_4$]$^-$ CF$_3$), 117.9 (septet, $^4J_{CF}$ = 4.0 Hz; [BAr$^F_4$]$^-$ *p*-C$_{aryl}$), 102.8 (vt, $^3J_{CP}$ = 2.3 Hz; heterocyclic C3,5), 65.3 (s; bound alkene CH), 36.8 (vt, J = 3.2 Hz; COD Ir-C$_{sp2}$-C_{sp3}), 31.9 (s; COD Ir-C$_{sp2}$-C$_{sp3}$-C_{sp3}), 31.0 (vt, $^1J_{CP}$ = 13.2 Hz; ^{i}Pr CH), 17.2 (vt, $^2J_{CP}$ = 2.4 Hz; ^{i}Pr CH$_3$), 16.3 (s; ^{i}Pr CH$_3$).

^{13}C{^{1}H} SSNMR (101 MHz, 10 kHz spin rate, 298 K) δ: 165.6 (heterocyclic C2,6), 165.1 (heterocyclic C2,6), 162−165 ([BAr$^F_4$]$^-$ *i*-C$_{aryl}$), 145.1 (heterocyclic C4), 121–139 (spectator COD C_{sp2} + [BAr$^F_4$]$^-$), 117-121 ([BAr$^F_4$]$^-$ *p*-C$_{aryl}$), 101.9 (heterocyclic C3,5), 68.2 (bound COD C_{sp2}), 26-40 (^{i}Pr CH + COD C_{sp3}), 11−20 (^{i}Pr CH$_3$),

^{31}P{^{1}H} NMR (162 MHz, CD_2Cl_2, 298 K) δ 184.4.

^{31}P{^{1}H} SSNMR (162 MHz, 10 kHz spin rate, 298 K) δ 194.4 (d, $^2J_{PP'}$ ~ 330 Hz), 185.4 (d, $^2J_{PP'}$ ~ 330 Hz),

^{19}F{^{1}H} NMR (377 MHz, CD_2Cl_2, 298 K) δ –62.8.

^{11}B{^{1}H} NMR (128 MHz, CD_2Cl_2, 298 K) δ –6.63.

Elemental analysis found (calculated) for $C_{57}H_{55}BF_{24}IrNO_2P_2$: C, 45.27 (45.43); H, 3.56 (3.68); N, 0.89 (0.93).

ESI-MS (CH_2Cl_2) *m/z* found (calculated) for $C_{25}H_{43}IrNO_2P_2$ [M]$^+$: 644.2426 (644.2394).

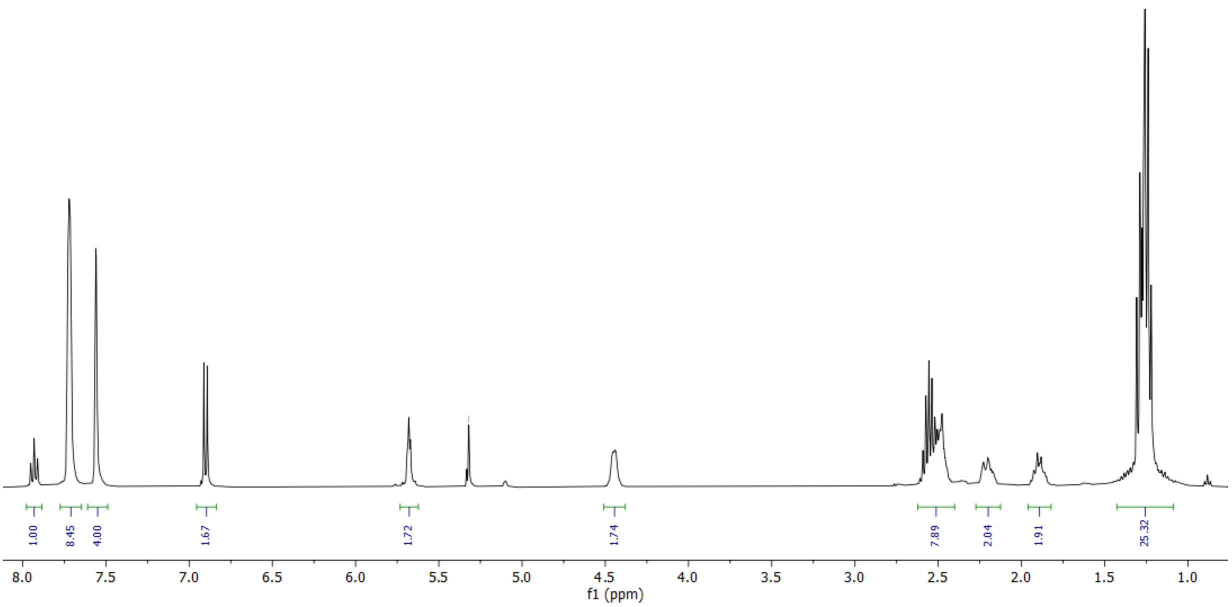

Figure S1 ^{1}H NMR spectrum of **1** (400.20 MHz, 298 K, CD_2Cl_2).

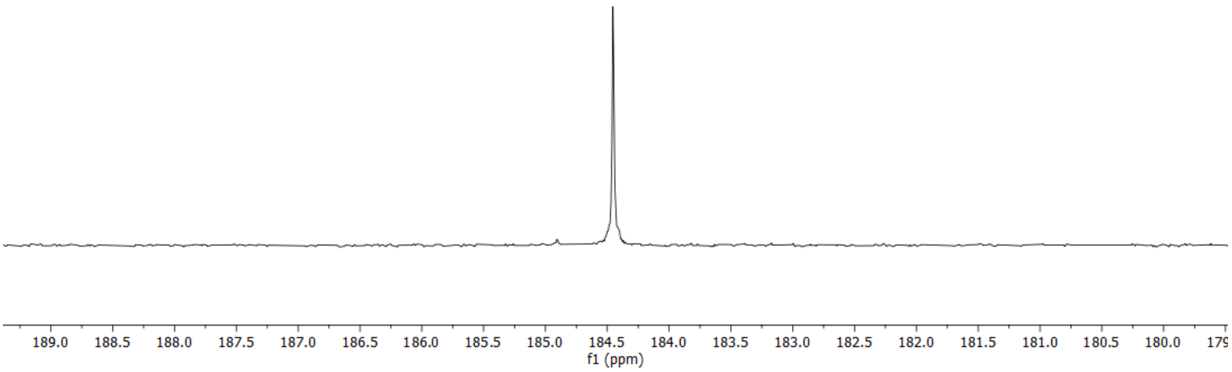

Figure S2 $^{31}P\{^{1}H\}$ NMR spectrum of **1** (162 MHz, 298 K, CD_2Cl_2).

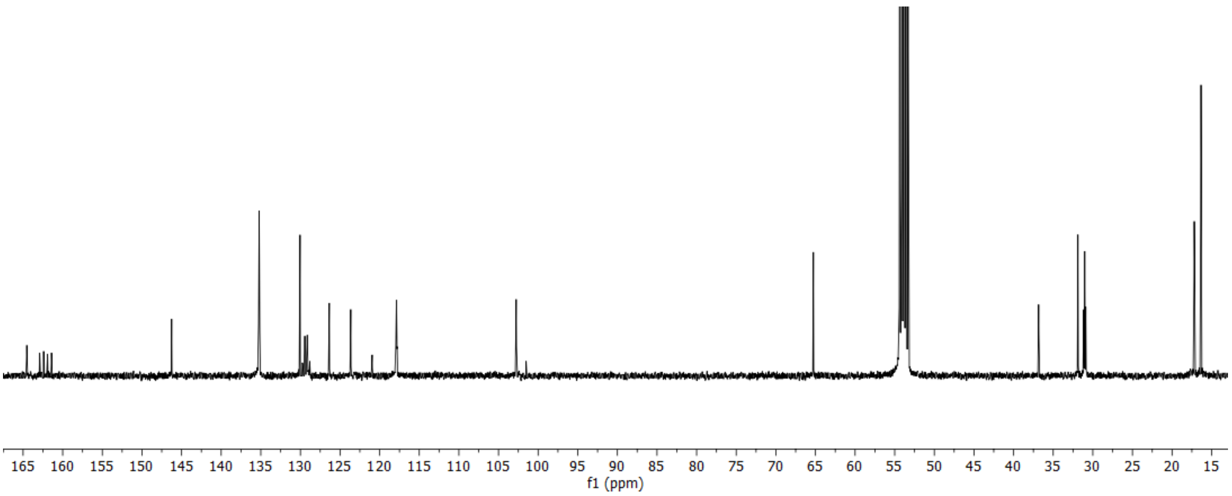

Figure S3 $^{13}C\{^{1}H\}$ NMR spectrum of **1** (100.64 MHz, 298 K, CD_2Cl_2).

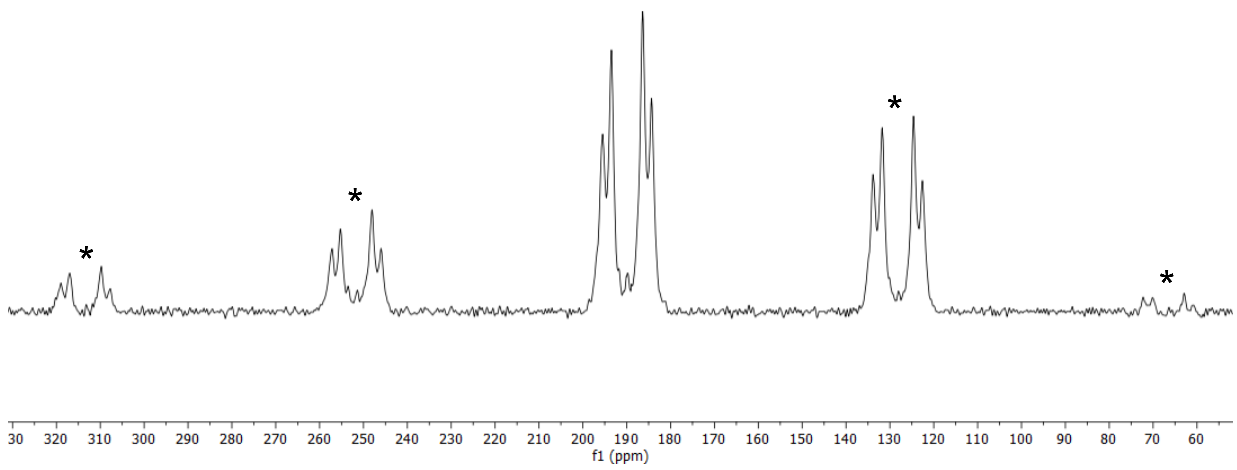

Figure S4 $^{31}P\{^1H\}$ SSNMR spectrum of **1** (162.03 MHz, 10 kHz spin rate, 298 K). * indicate spinning side-bands.

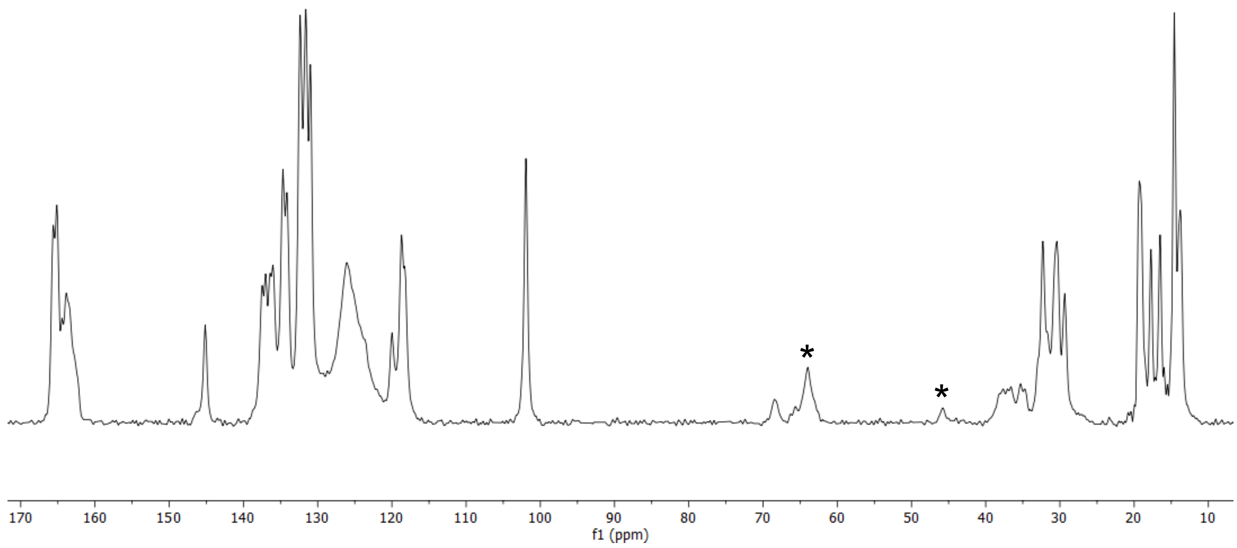

Figure S5 $^{13}C\{^1H\}$ SSNMR spectrum of **1** (100.66 MHz, 10 kHz spin rate, 298 K). * indicate spinning side-bands.

1.2.2. Synthesis of [(^{i}Pr-PONOP)IrH$_2$(H$_2$)][BAr$^F_4$] (2)

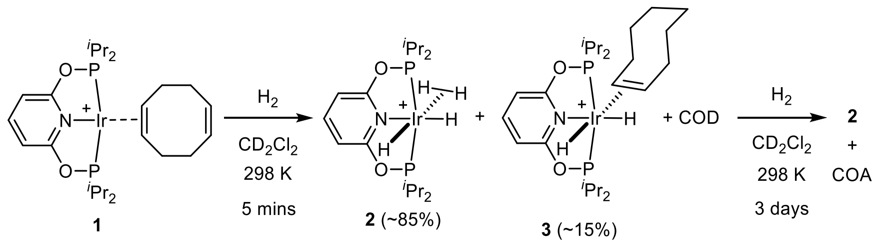

[(^{i}Pr-PONOP)Ir(η^2-COD)][BAr$^F_4$] (15.3 mg, 10.2 μmol) was introduced to a medium-walled NMR tube fitted with a controlled atmosphere valve and dissolved in CD_2Cl_2 (0.5 mL) that was added *via* vacuum transfer. The tube was charged with H_2 (4.0 bar absolute), upon shaking, the solution turned from orange to a pale golden-yellow, generating the non-classical 'tetrahydride' [(^{i}Pr-PONOP)IrH$_2$(H$_2$)][BAr$^F_4$] (**2**) *in situ* alongside [(^{i}Pr-PONOP)IrH$_2$(COE)][BAr$^F_4$] (**3**) (85:15 ratio **2**:**3**) and free 1,5-cyclooctadiene (COD). Complete hydrogenation of this COD to cyclooctane and corresponding quantitative conversion to **2** takes 72 hours under these conditions.

Spin-lattice relaxation time (T_1) measurements on the hydride resonance were carried out by the inversion-recovery method using standard[7,8] 180°-θ-90° pulse sequence: 119 ± 8 ms (295 K) and 38 ± 1 ms (253 K) under a pressure of H_2 (1.5 bar).

In the absence of an H_2 atmosphere, solutions of **2** lost H_2 to yield the dihydride [(^{i}Pr-PONOP)IrH$_2$][BAr$^F_4$] (**4**; *vide infra*).

NMR data for **2:**

^{1}H NMR (500 MHz, CD_2Cl_2, 298 K) δ: 7.79 (t, $^3J_{HH}$ = 8.2 Hz, 1 H; heterocyclic C4), 7.72 (br. m, 8 H; [BAr$^F_4$]$^-$ *o*-H), 7.56 (s, 4 H; [BAr$^F_4$]$^-$ *p*-H), 6.85 (d, $^3J_{HH}$ = 8.0 Hz, 2 H; heterocyclic C3,5), 2.48-2.40 (m, 4 H; ^{i}Pr CH), *1.53 (liberated cyclooctane),* 1.21 (m, $^3J_{HH}$ = 6.9 Hz, 12 H; ^{i}Pr CH$_3$), 1.16 (m, $^3J_{HH}$ = 7.0 Hz, 12 H; ^{i}Pr CH$_3$), –8.79 (br. s, 4 H; Ir-*H*).

^{1}H NMR (500 MHz, CD_2Cl_2, 253 K) δ: 7.76 (t, $^3J_{HH}$ = 8.2 Hz, 1 H; heterocyclic C4), 7.71 (br. m, 8 H; [BAr$^F_4$]$^-$ *o*-H), 7.55 (s, 4 H; [BAr$^F_4$]$^-$ *p*-H), 6.83 (d, $^3J_{HH}$ = 8.1 Hz, 2 H; heterocyclic C3,5), 2.43-2.37 (m, 4 H; ^{i}Pr CH), *1.49 (liberated cyclooctane),* 1.16 (m, $^3J_{HH}$ = 7.1 Hz, 12 H; ^{i}Pr CH$_3$), 1.12 (m, $^3J_{HH}$ = 7.0 Hz, 12 H; ^{i}Pr CH$_3$), –8.81 (br. t, $^2J_{HP}$ 7.5 Hz, 4 H; Ir-*H*).

^{31}P{^{1}H} NMR (203 MHz, CD_2Cl_2, 298 K) δ: 181.2 (s).

^{31}P{^{1}H} NMR (203 MHz, CD_2Cl_2, 253 K) δ: 181.2 (s).

^{13}C{^{1}H} NMR (151 MHz, CD_2Cl_2, 298 K) δ: 162.2 (q, $^1J_{CB}$ = 49.8 Hz (^{11}B); [BAr$^F_4$]$^-$ *i*-C$_{aryl}$), 161.1 (s; heterocyclic C2,6), 145.3 (s; heterocyclic C4), 135.2 (s; [BAr$^F_4$]$^-$ *o*-C$_{aryl}$), 129.3 (qq, $^2J_{CF}$ = 31.5 Hz, $^4J_{CF}$ = 2.9 Hz; [BAr$^F_4$]$^-$ C$_{aryl}$-CF$_3$), 125.0 (q, $^1J_{CF}$ = 272.6 Hz; [BAr$^F_4$]$^-$ CF$_3$), 117.9 (septet, $^4J_{CF}$ = 4.0 Hz; [BAr$^F_4$]$^-$ *p*-C$_{aryl}$), 103.9 (s; heterocyclic C3,5), 31.3 (vt, $^1J_{CP}$ = 15.8 Hz; ^{i}Pr CH), 17.7 (br. vt, $^2J_{CP}$ ~ 3 Hz; ^{i}Pr CH$_3$), 17.3 (s; ^{i}Pr CH$_3$).

Selected NMR data for **3**, free COD and dissolved H_2 present in mixture after 5 minutes:

^{1}H NMR (500 MHz, CD_2Cl_2, 298 K) δ: 6.79 (d, $^1J_{HH}$ = 8.2 Hz, 1 H; heterocyclic C3/5), 6.76 (d, $^1J_{HH}$ 8.1 Hz, 1H; heterocyclic C3/5), 5.55 (br. s, 4H; *liberated* COD), 4.60 (*dissolved* H_2), 3.04-2.97 (m, 1H, alkene), 2.96-2.91 (m, 1H, alkene), 2.77-2.68 (m, 2H; ^{i}Pr CH), 2.35 (br. s, 8H; *liberated* COD), 1.56 (dd, $^3J_{PH}$ =

12.3 Hz, $^3J_{HH}$ 7.0 Hz, 3H; ^{i}Pr); 1.53 (m, overlapping signals; COA, ^{i}Pr), 1.38 (dd, $^3J_{PH}$ = 19.2 Hz, $^3J_{HH}$ = 7.5 Hz, 3H; ^{i}Pr) 1.32 (dd, $^3J_{PH}$ = 19.8 Hz, $^3J_{HH}$ = 7.6 Hz, 3H; ^{i}Pr), 0.84 (dd, $^3J_{PH}$ = 15.4 Hz, $^3J_{HH}$ = 6.9 Hz, 3H; ^{i}Pr), 0.79 (dd, $^3J_{PH}$ = ~15 Hz, $^3J_{HH}$ = 6.6 Hz, 3H; ^{i}Pr), –10.41 (vdt, $^3J_{PH}$ = 20.7 Hz, $^3J_{PH}$ = 19.6 Hz, 1H; Ir-H), –17.77 (vdt, $^3J_{PH}$ = 10.3 Hz, $^3J_{PH}$ 10.1 Hz, 1H; Ir-H).

$^{31}P\{^1H\}$ NMR (203 MHz, CD_2Cl_2, 298 K) δ: 170.7 (d, $^2J_{PP}$ 280 Hz), 167.8 (d, $^2J_{PP}$ 280 Hz).

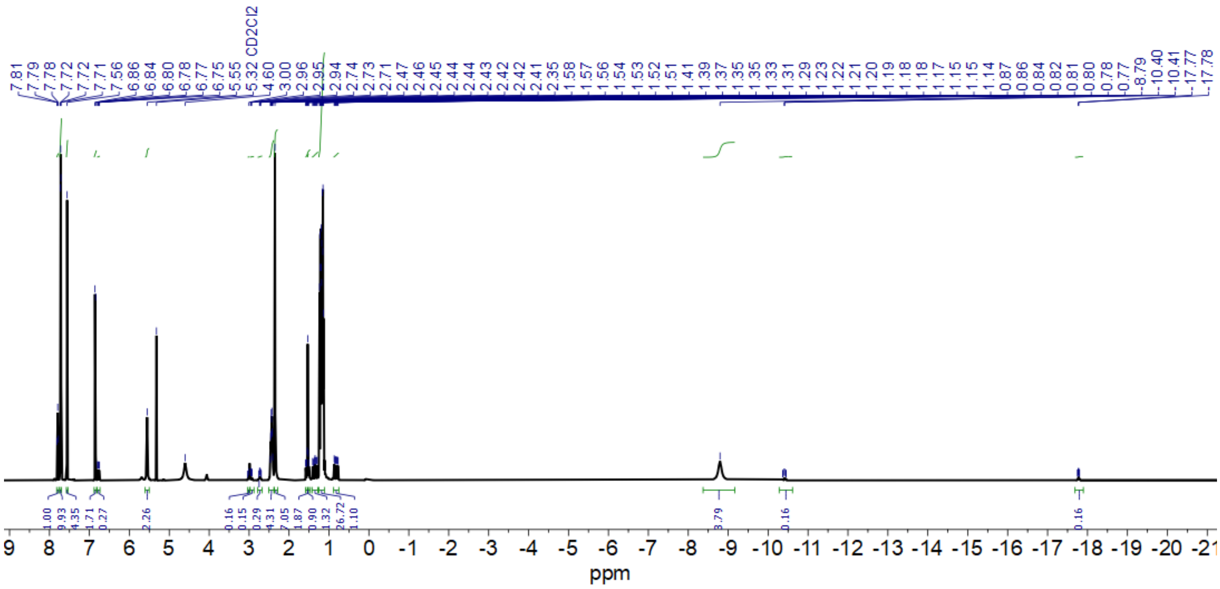

Figure S6 ^1H NMR (500 MHz, 298 K, CD_2Cl_2) spectrum of mixture of **2** and **3** generated *in situ* from **1** (15.3 mg) and H_2 (4.0 bar, 298 K, 5 minutes).

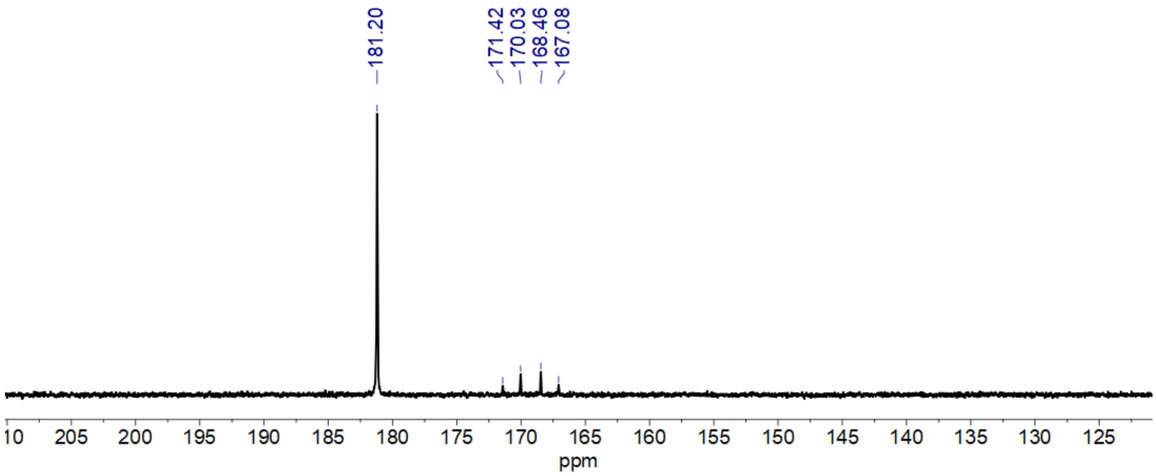

Figure S7 $^{31}P\{^1H\}$ NMR (202 MHz, 298 K, CD_2Cl_2) spectrum of mixture of **2** and **3** generated *in situ* from **1** (15.3 mg) and H_2 (4.0 bar, 298 K, 5 minutes).

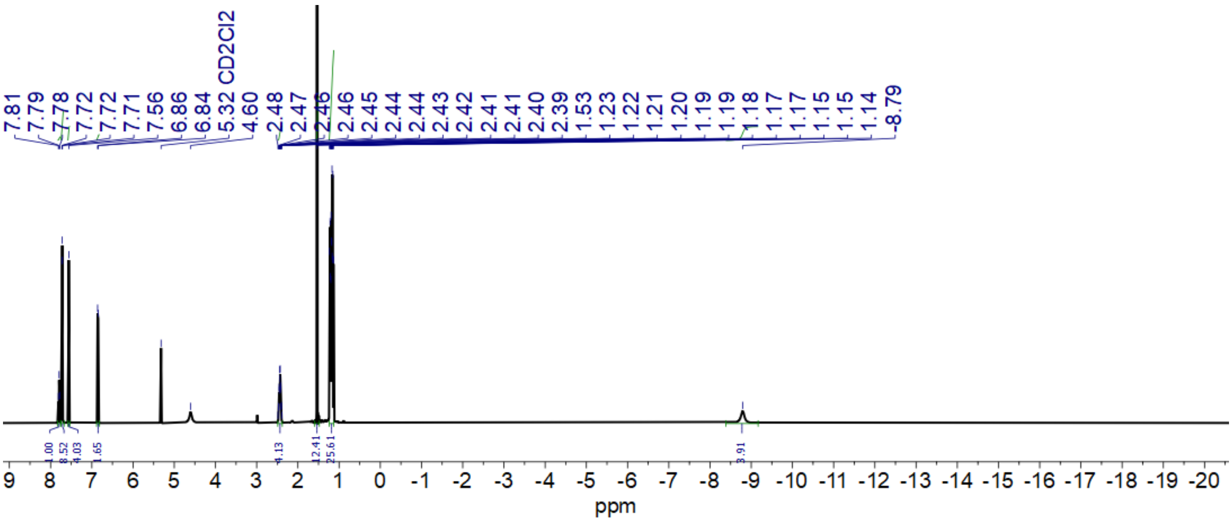

Figure S8 ^{1}H NMR (500 MHz, 298 K, CD_2Cl_2) spectrum of **2** generated *in situ* from **1** (15.3 mg) and H_2 (4.0 bar, 298 K, 72 hours). δ at 1.53 is cyclooctane.

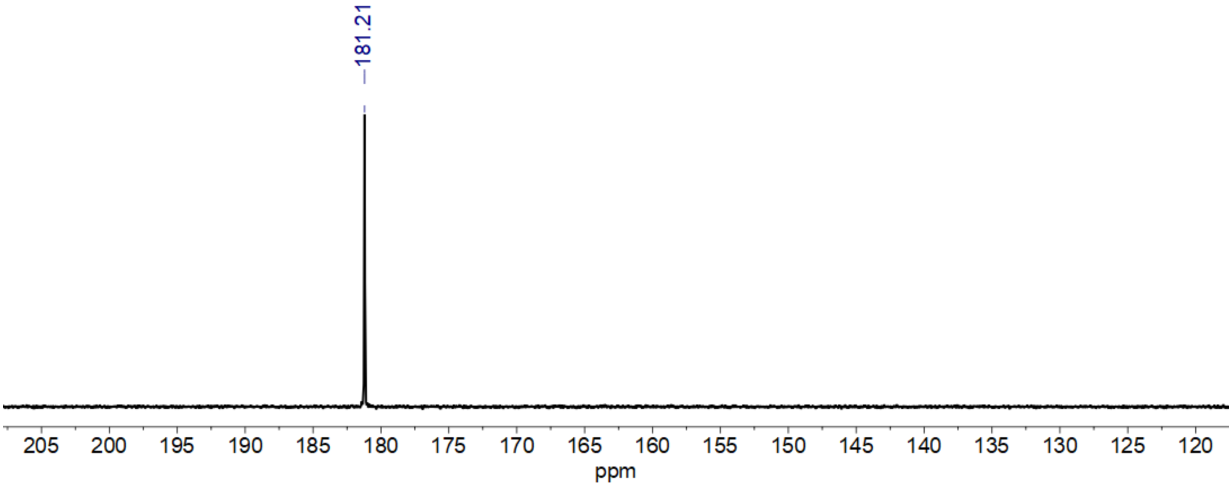

Figure S9 ^{31}P{^{1}H} NMR (202 MHz, 298 K, CD_2Cl_2) spectrum of mixture of **2** generated *in situ* from **1** (15.3 mg) and H_2 (4.0 bar, 298 K, 72 hours).

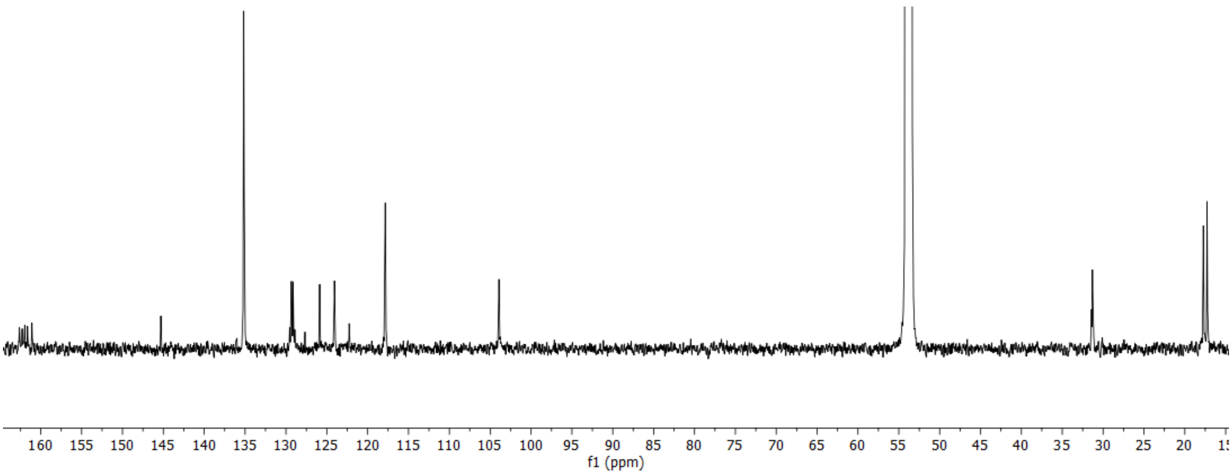

Figure S10 ^{13}C{^{1}H} NMR spectrum of **2** (151 MHz, 298 K, CD_2Cl_2), generated *in situ* from **4** (~2 mg; *vide infra*) under an atmosphere of H_2 (1.5 bar).

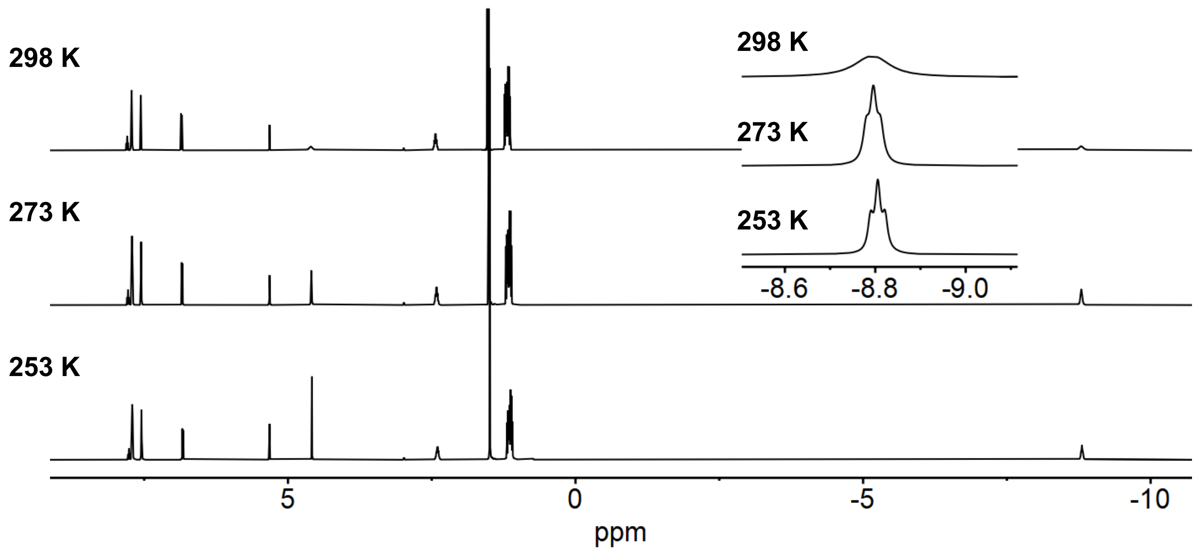

Figure S11 ^{1}H VT-NMR (500 MHz, CD_2Cl_2) spectrum of **2** generated *in situ* from **1** (15.3 mg) and H_2 (4.0 bar, 298 K, 72 hours). δ at 1.53 is cyclooctane.

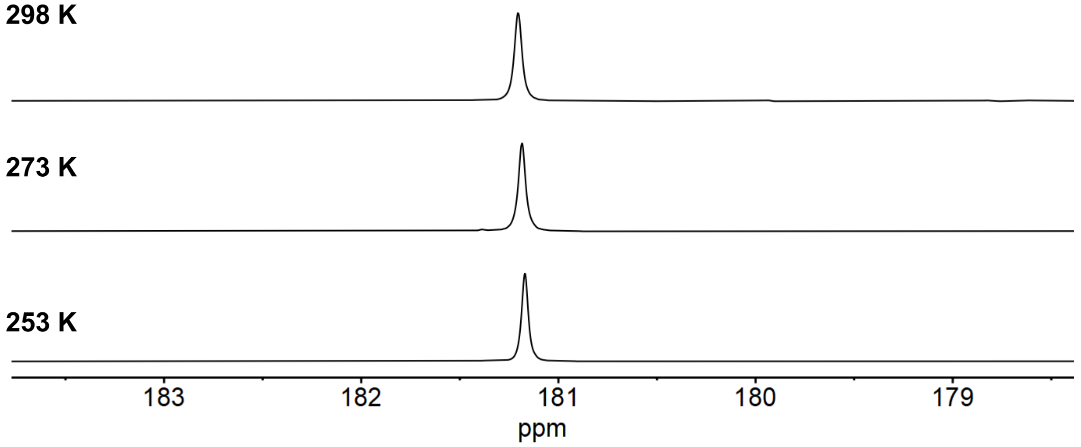

Figure S12 $^{31}P\{^{1}H\}$ NMR (202 MHz, CD_2Cl_2) spectrum of mixture of **2** generated *in situ* from **1** (15.3 mg) and H_2 (4.0 bar, 298 K, 72 hours).

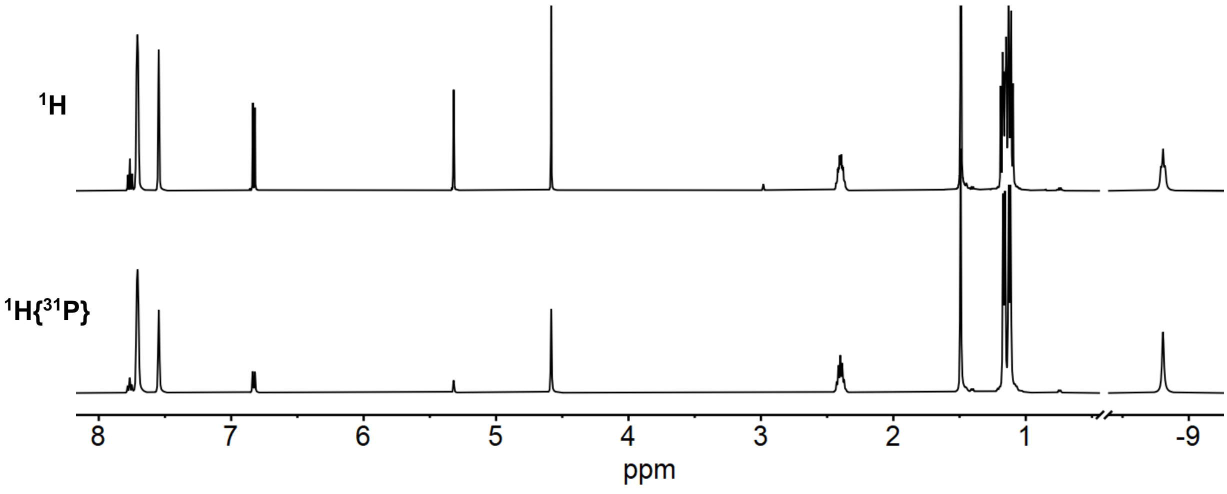

Figure S13 ^{1}H NMR and $^{1}H\{^{31}P\}$ NMR (500 MHz, 253 K, CD_2Cl_2) spectrum of **2** generated *in situ* from **1** (15.3 mg) and H_2 (4.0 bar, 298 K, 72 hours). δ at 1.53 is cyclooctane.

1.2.3. Synthesis of [(^{i}Pr-PONOP)IrH$_2$][BAr$^F{}_4$] (4)

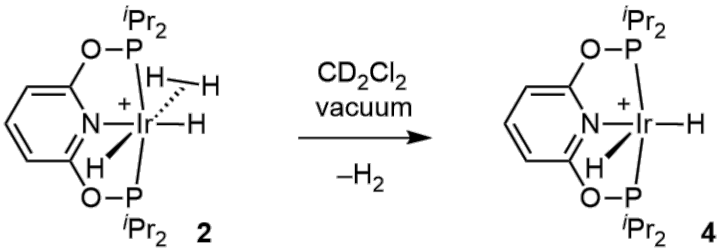

Samples of **2** as generated above from **1** *in situ* were refrozen with liquid N_2 and the headspaces of the tubes were evacuated; upon thawing, the dihydride [(^{i}Pr-PONOP)IrH$_2$][BAr$^F{}_4$] (**4**) slowly formed over the course of 3-6 hours, encouraged by further freeze-pump-thaw cycles. Near-pure samples of dihydride **4** were obtained by removing all volatiles (including liberated cyclooctane) *in vacuo*, leaving behind the dihydride as an oily orange solid.

Spin-lattice relaxation time (T_1) measurements on the hydride position were carried out by the inversion-recovery method using the standard 180°-θ-90° pulse sequence: 1870 ± 10 ms (295 K).

^{1}H NMR (600 MHz, CD$_2$Cl$_2$, 298 K) δ: 7.83 (t, $^3J_{HH}$ = 8.2 Hz, 1 H; heterocyclic C4), 7.71 (br. m, 8 H; [BAr$^F{}_4$]$^-$ *o*-H), 7.56 (s, 4 H; [BAr$^F{}_4$]$^-$ *p*-H), 6.90 (d, $^3J_{HH}$ = 8.2 Hz, 2 H; heterocyclic C3,5), 2.68 (br. septet, $^3J_{HH}$ ~ 7 Hz, 4 H; ^{i}Pr CH), 1.33 (vq, $^3J_{HH}$ = 6.8 Hz, $^3J_{HP}$ ~ 8 Hz, $^3J_{HP'}$ ~ 9 Hz 12 H; ^{i}Pr CH$_3$), 1.16 (vq, $^3J_{HH}$ = 7.1 Hz, $^3J_{HP}$ ~ 8 Hz, $^3J_{HP'}$ ~ 9 Hz, 12 H; ^{i}Pr CH$_3$), −19.82 (br. s, 2 H; Ir-*H*).

^{31}P{^{1}H} NMR (243 MHz, CD$_2$Cl$_2$, 298 K) δ 183.3 (br.).

^{31}P{^{1}H} NMR (243 MHz, 1,2-difluorobenzene, 298 K) δ 186.5 (br.).

^{13}C{^{1}H} NMR (151 MHz, CD$_2$Cl$_2$, 298 K) δ: 162.2 (q, $^1J_{CB}$ = 49.8 Hz (^{11}B); [BAr$^F{}_4$]$^-$ *i*-C$_{aryl}$), 161.6 (s; heterocyclic C2,6), 145.0 (s; heterocyclic C4), 135.2 (s; [BAr$^F{}_4$]$^-$ *o*-C$_{aryl}$), 129.3 (qq, $^2J_{CF}$ = 31.5 Hz, $^4J_{CF}$ = 2.9 Hz; [BAr$^F{}_4$]$^-$ C$_{aryl}$-CF$_3$), 125.0 (q, $^1J_{CF}$ = 272.6 Hz; [BAr$^F{}_4$]$^-$ CF$_3$), 117.9 (septet, $^4J_{CF}$ = 4.0 Hz; [BAr$^F{}_4$]$^-$ *p*-C$_{aryl}$), 103.8 (s; heterocyclic C3,5), 30.5 (vt, $^1J_{CP}$ = 14.5 Hz; ^{i}Pr CH), 18.0 (br. vt, $^2J_{CP}$ ~ 2 Hz; ^{i}Pr CH$_3$), 17.3 (s; ^{i}Pr CH$_3$),

ESI-MS (CH$_2$Cl$_2$) *m*/*z* found (calculated) for $C_{17}H_{33}IrNO_2P_2$ [M]$^+$: 538.1604 (538.1616); ~60% relative intensity for [(^{i}Pr-PONOP)Ir]$^+$: 536.1460 (536.1459); ~5% relative intensity for [(^{i}Pr-PONOP)Ir(N$_2$)]$^+$: 564.1403 (564.1521).

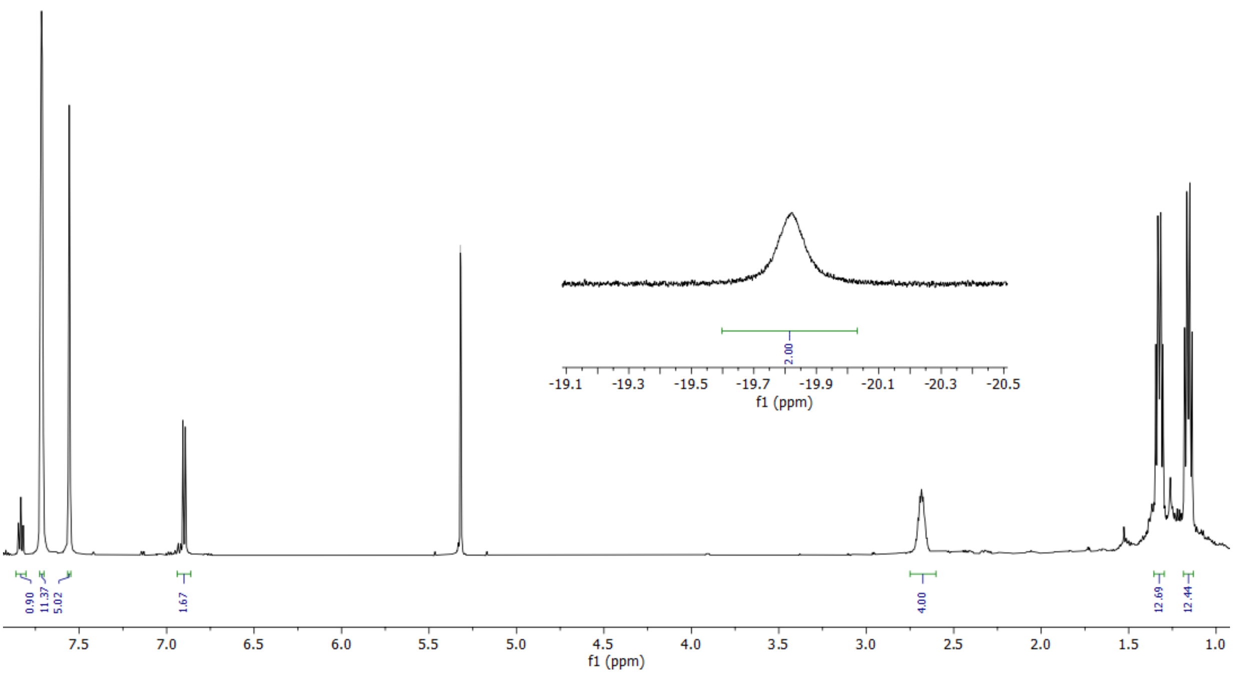

Figure S14: ^{1}H NMR spectrum of **4** (600 MHz, 298 K, CD_2Cl_2).

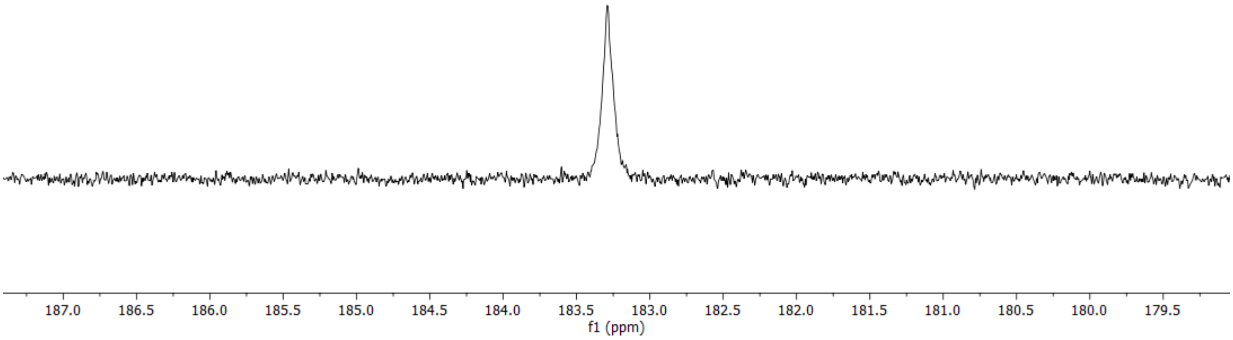

Figure S15: ^{31}P{^{1}H} NMR spectrum of **4** (243 MHz, 298 K, CD_2Cl_2).

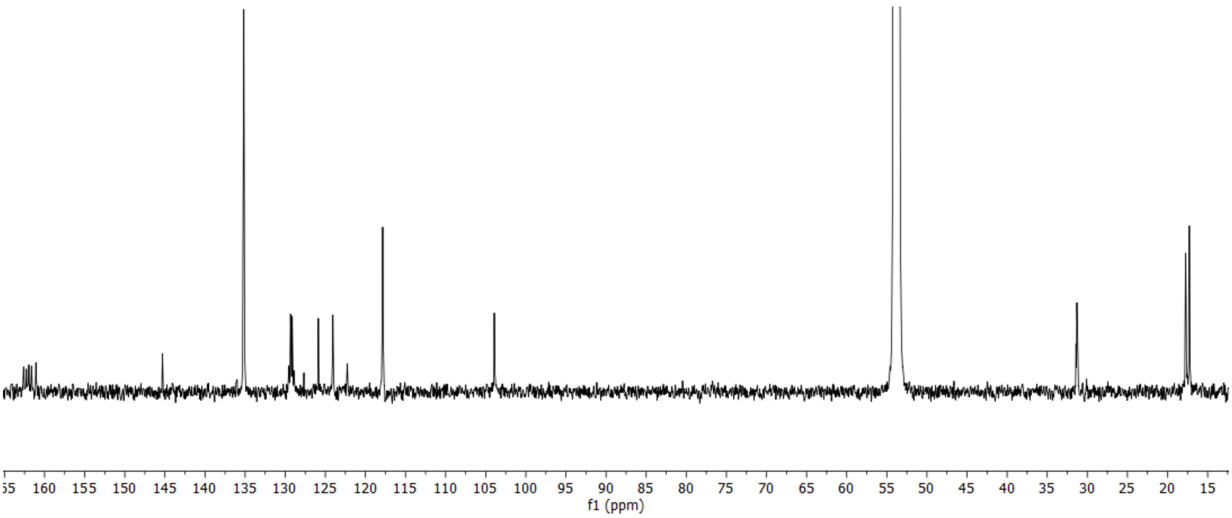

Figure S16: ^{13}C{^{1}H} NMR spectrum of **4** (151 MHz, 298 K, CD_2Cl_2).

1.2.4. Synthesis of [(^{i}Pr-PONOP)IrH$_2$(σ-H$_3$BNMe$_3$)][BAr$^F_4$] (5)

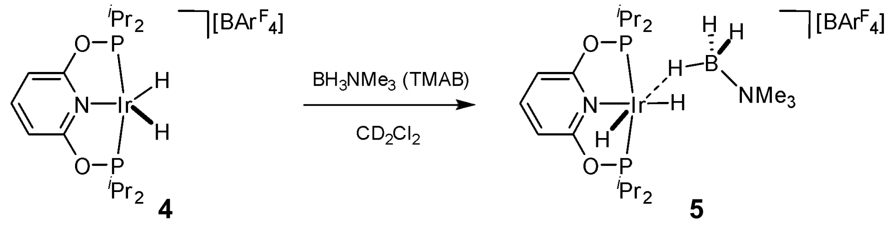

A solution of Complex **1** (10 mg, ~7 μmol) in CH_2Cl_2 (0.4 mL) in a medium-walled NMR tube fitted with a controlled atmosphere valve was freeze-pump-thaw degassed and the tube was repressurised with H_2 (4 bar absolute). Upon thawing and shaking, the solution turned from orange to a pale golden-yellow, generating non-classical 'tetrahydride' **2** *in situ*. After this, all volatiles were removed *in vacuo*, leaving behind the dihydride **4** as an oily orange solid. An excess of trimethylamine-borane (TMAB; ~0.7 mg, ~10 μmol) was placed in the tube and the solids were redissolved in CD_2Cl_2 (0.4 mL), generating a colourless solution, which was interrogated by solution-phase NMR spectroscopy, revealing the TMAB-dihydride [(^{i}Pr-PONOP)IrH$_2$(σ-H$_3$BNMe$_3$)][BAr$^F_4$] (**5**) as generated *in situ*, in addition to non-stoichiometric free TMAB (*ca*. 30% by ^{1}H NMR integrals). The contents of the NMR tube were layered with pentane at 298 K to yield a limited number of small, colourless crystals of TMAB-dihydride **5** after one week, amenable to *sc*-XRD studies.

OR

Complex **1** (70 mg, 46 μmol) and H$_3$B•NMe$_3$ (20 mg, 0.27 mmol) were dissolved in CH_2Cl_2 (5 mL) and the resulting solution degassed by three successive freeze-pump-thaw cycles before charging with H_2 (4 bar). The resulting solution was stirred at room temperature overnight, after which time *in situ* NMR spectroscopic interrogation displayed complete conversion to **5**. All volatile components were removed and the residual white solid was washed with pentane (3 × 5 mL) and dried under reduced pressure to yield **5** as a white powder (58 mg, 40 mmol, 87%). This method allows for the isolation of analytically pure solid.

^{1}H NMR (600 MHz, CD_2Cl_2, 298 K) δ: 7.72 (br. m, 8 H; [BAr$^F_4$]$^-$ *o*-H), 7.69 (t, $^3J_{HH}$ = 8.2 Hz, 1 H; heterocyclic C4), 7.56 (s, 4 H; [BAr$^F_4$]$^-$ *p*-H), 6.76 (d, $^3J_{HH}$ = 8.2 Hz, 2 H; heterocyclic C3,5), 2.85 (septet, $^3J_{HH}$ = 7.1 Hz, 2 H; ^{i}Pr CH), 2.63 (s, 9 H; NMe$_3$), 2.59-2.53 (m, 2 H; ^{i}Pr CH), 1.49 (vq, $^3J_{HH}$ = 6.6 Hz, $^3J_{HP}$ 6.6 Hz, 6 H; ^{i}Pr CH$_3$), 1.39-1.34 (m, 6 H; ^{i}Pr CH$_3$), 1.13-1.08 (m, $^3J_{HH}$ = 6.9 Hz, $^3J_{HP}$ 10.3 Hz, 6 H; ^{i}Pr CH$_3$), 0.88 (vq, $^3J_{HH}$ = 7.4 Hz, $^3J_{HP}$ 6.5 Hz, 6 H; ^{i}Pr CH$_3$), −2.20 (br. s, FWHM 390 Hz, 3 H; BH$_3$), −15.91 (td, $^2J_{HP}$ = 12.0 Hz, $^2J_{HH}$ = 5.6 Hz, 1 H; Ir-*H*), −20.01 (br. td, $^2J_{HP}$ = 16.9 Hz, $^2J_{HH}$ ~4 Hz, 1 H; Ir-*H*),

^{31}P{^{1}H} NMR (243 MHz, CD_2Cl_2, 298 K) δ 179.8.

^{11}B{^{1}H} NMR (193 MHz, CD_2Cl_2, 298 K) δ –6.6 (s; [BAr$^F_4$]$^-$), −14.2 (br. S, FWHM 325 Hz; σ-H$_3$BNMe$_3$).

Elemental analysis found (calculated) for $C_{52}H_{57}N_2OP_2F_{24}B_2Ir$: C, 42.25 (42.84); H, 3.64 (3.94); N, 1.65 (1.92).

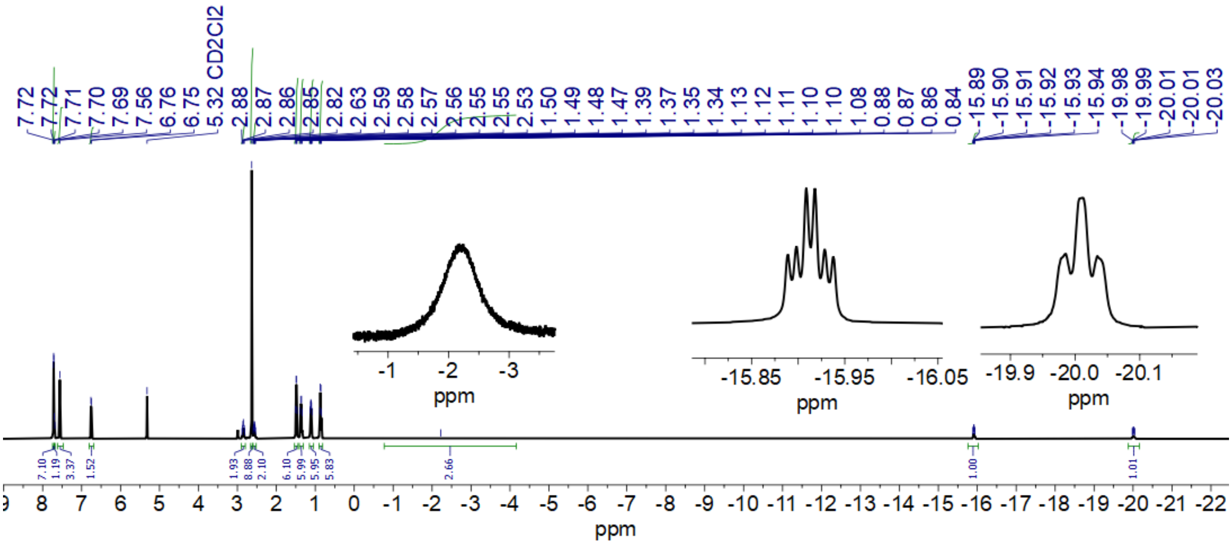

Figure S17 ^{1}H NMR (600 MHz, 298 K, CD_2Cl_2) spectrum of **5.**

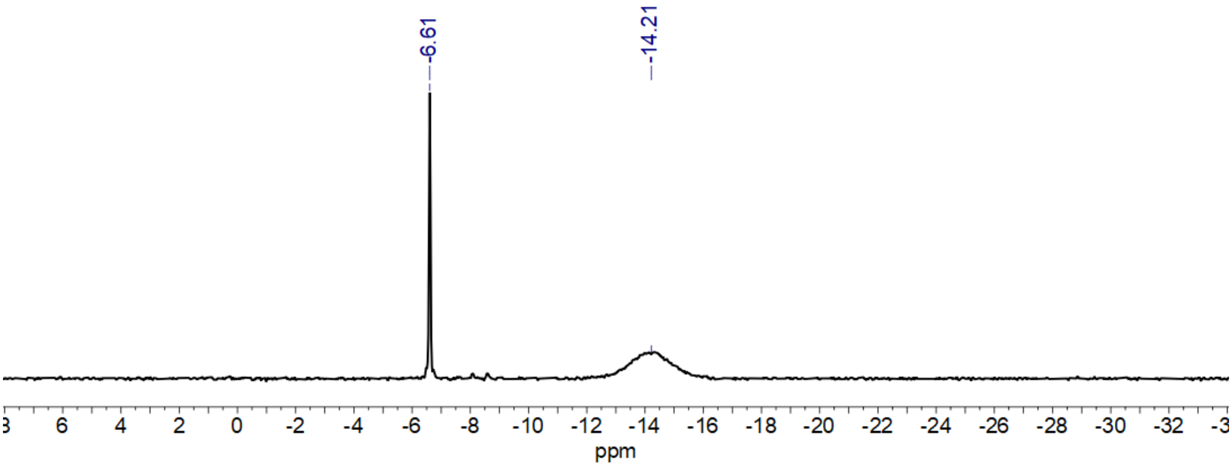

Figure S18 ^{11}B NMR (192 MHz, 298 K, CD_2Cl_2) spectrum **5**.

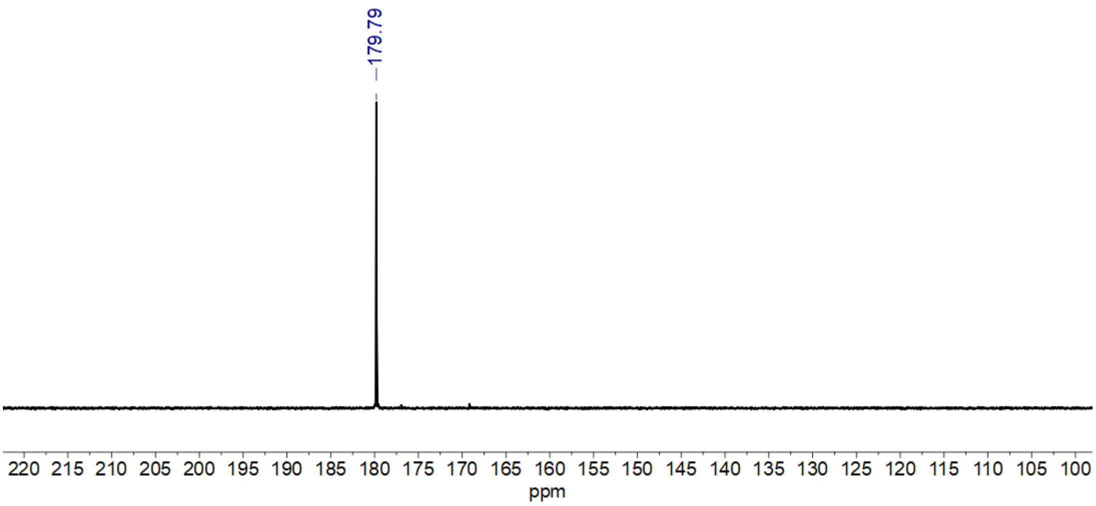

Figure S19 $^{31}P\{^{1}H\}$ NMR (203 MHz, 298 K, CD_2Cl_2) spectrum of **5**.

1.2.5. Synthesis of [(^{i}Pr-PONOP)Ir(η^2-propene)][BAr$^F{}_4$] (6)

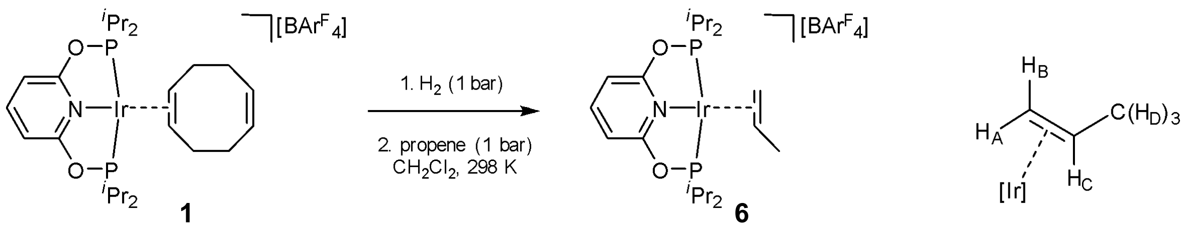

A solution of complex **1** (100 mg, 66.4 μmol) in CH_2Cl_2 (5 mL) was freeze-pump-thaw degassed and placed under an atmosphere of H_2 (4 bar absolute) and stirred at room temperature for three days, over which time the initially orange-red solution turned colourless.. The solution was again freeze-pump-thaw degassed and placed under propene (2 bar absolute). The solution was stirred at room temperature for 24 hours; a colour change from pale golden-yellow to bright orange was observed. All volatiles were then removed *in vacuo* to yield an amorphous orange solid. The solid was dried *in vacuo* overnight, redissolved in 1,2-difluorobenzene (2 mL), layered with excess pentane and stored at room temperature to yield orange blocks of [(^{i}Pr-PONOP)Ir(η^2-propene)][BAr$^F{}_4$] (**6**; 90 mg, 94%) suitable for *sc*-XRD studies after two weeks.

^{1}H NMR (500 MHz, CD_2Cl_2, 298 K) δ: 7.95 (tt, $^3J_{HH}$ = 8.0 Hz, $^5J_{HP}$ = 1.0 Hz, 1 H; heterocyclic C4), 7.76 (br. m, 8 H; [BAr$^F{}_4$]$^-$ *o*-H), 7.60 (s br, 4 H; [BAr$^F{}_4$]$^-$ *p*-H), 6.92 (d, $^3J_{HH}$ = 8.0 Hz, 2 H; heterocyclic C3,5), 4.33 (ddqt, $^3J_{HCHD}$ = 5.8 Hz, $^3J_{HCHA}$ = 7.5 Hz, $^3J_{HCHB}$ = 10.6 Hz, $^3J_{HP}$ ~ 5.5 Hz, 1 H; H_C), 3.40 (dt, $^3J_{HBHC}$ = 10.6 Hz, $^3J_{HP}$ = 6.3 Hz, 1 H; H_B), 3.08 (d, $^3J_{HAHC}$ = 7.5 Hz, 1 H; H_A), 2.77 (septet, $^3J_{HP}$ = 7.2 Hz, 2 H; ^{i}Pr CH), 2.57 (br. septet, $^3J_{HP}$ ~ 7.0 Hz, 2 H; ^{i}Pr CH), 1.72 (d, $^3J_{HDHC}$ = 5.8 Hz, 3 H; H_D), 1.38 (dd, $^3J_{HH}$ ~ 6.5 Hz, $^3J_{HP}$ = 7.5 Hz, 6 H; ^{i}Pr CH_3), 1.36 (dd, $^3J_{HH}$ ~ 6.5 Hz, $^3J_{HP}$ 7.0 Hz, 6 H; ^{i}Pr CH_3),1.14 (dd, $^3J_{HH}$ ~ 6.5 Hz, $^3J_{HP}$ = 7.0 Hz, 6 H; ^{i}Pr CH_3), 1.13 (dd, $^3J_{HH}$ ~ $^3J_{HP}$ = 7.0 Hz, 6 H; ^{i}Pr CH_3).

^{1}H NMR (500 MHz, CD_2Cl_2, 183 K) δ: 7.87 (t, $^3J_{HH}$ = 8.0 Hz, 1 H; heterocyclic C4), 7.72 (br. m, 8 H; [BAr$^F{}_4$]$^-$ *o*-H), 7.53 (s br, 4 H; [BAr$^F{}_4$]$^-$ *p*-H), 6.86 (d, $^3J_{HH}$ = 8.0 Hz, 2 H; heterocyclic C3,5), 4.19 (app. sept, J = 6 Hz , 1 H; H_C), 3.30 (dt, $^3J_{HBHC}$ = 10.5 Hz, $^3J_{HP}$ = 6.0 Hz, 1 H; H_B), 2.95 (d, $^3J_{HAHC}$ = 7.5 Hz, 1 H; H_A), 2.68 (septet, $^3J_{HP}$ = 7.0 Hz, 2 H; ^{i}Pr CH), 2.49 (septet, $^3J_{HP}$ = 7.0 Hz, 2 H; ^{i}Pr CH), 1.57 (d, $^3J_{HDHC}$ = 6.0 Hz, 3 H; H_D), 1.16-1.32 (m, 12 H; ^{i}Pr CH_3), 0.89-1.05 (m, 12 H; ^{i}Pr CH_3).

^{13}C{^{1}H} NMR (126 MHz, CD_2Cl_2, 298 K) δ: 164.2 (vt, $^2J_{CP}$ = 3.5 Hz; heterocyclic C2,6), 162.1 (q, $^1J_{CB}$ = 49.9 Hz (^{11}B); [BAr$^F{}_4$]$^-$ *i*-C_{aryl}), 145.4 (s; heterocyclic C4), 135.2 (m; [BAr$^F{}_4$]$^-$ *o*-C_{aryl}), 129.2 (qq, $^2J_{CF}$ = 31.5 Hz, $^4J_{CF}$ = 3.0 Hz; [BAr$^F{}_4$]$^-$ C_{aryl}-CF_3), 124.9 (q, $^1J_{CF}$ = 272.6 Hz; [BAr$^F{}_4$]$^-$ CF_3), 117.9 (septet, $^4J_{CF}$ = 4.0 Hz; [BAr$^F{}_4$]$^-$ *p*-C_{aryl}), 103.2 (vt, $^3J_{CP}$ = 2.7 Hz; heterocyclic C3,5), 54.8 (s; alkene CH), 42.6 (s; alkene CH_2), 31.4 (vt, $^1J_{CP}$ = 13.7 Hz; ^{i}Pr CH), 30.5 (vt, $^1J_{CP}$ = 14.3 Hz; ^{i}Pr CH), 23.9 (s; alkene CH_3), 17.8 (vt, $^2J_{CP}$ = 2.0 Hz; ^{i}Pr CH_3), 16.8 (s; ^{i}Pr CH_3), 16.4 (s; ^{i}Pr CH_3), 16.3 (vt, $^2J_{CP}$ = 2.9 Hz; ^{i}Pr CH_3).

^{13}C{^{1}H} SSNMR (101 MHz, 10 kHz spin rate, 298 K) δ: 165.2 (heterocyclic C2,6), 162−167 ([BAr$^F{}_4$]$^-$ *i*-C_{aryl}), 144.0 (heterocyclic C4), 143.6 (heterocyclic C4), 121–140 ([BAr$^F{}_4$]$^-$), 117.7-120.0 ([BAr$^F{}_4$]$^-$ *p*-C_{aryl}), 102.9 (heterocyclic C3,5), 57.4 (alkene CH), 56.6 (alkene CH), 44.8 (alkene CH_2), 42.9 (alkene CH_2), ~32.0 (^{i}Pr CH), 29.7 (^{i}Pr CH), 23.3 (alkene CH_3), 23.0 (alkene CH_3), 13−20 (^{i}Pr CH_3).

^{31}P{^{1}H} NMR (202 MHz, CD_2Cl_2, 298 K) δ 188.6.

^{31}P{^{1}H} NMR (202 MHz, CD_2Cl_2, 183 K) δ 188.9 (br, fwhm = 70 Hz).

$^{31}P\{^1H\}$ SSNMR (162 MHz, 10 kHz spin rate, 298 K) δ 194.1 (d, $^2J_{PP'}$ ~ 310 Hz), 189.5 (d, $^2J_{PP'}$ ~ 310 Hz).

ESI-MS (CH_2Cl_2) m/z found (calculated) for $C_{20}H_{37}IrNO_2P_2$ $[M]^+$: 578.1930 (578.1923).

Elemental analysis found (calculated) for $C_{52}H_{49}BF_{24}IrNO_2P_2$: C, 43.10 (43.35); H, 3.39 (3.43); N, 0.85 (0.97).

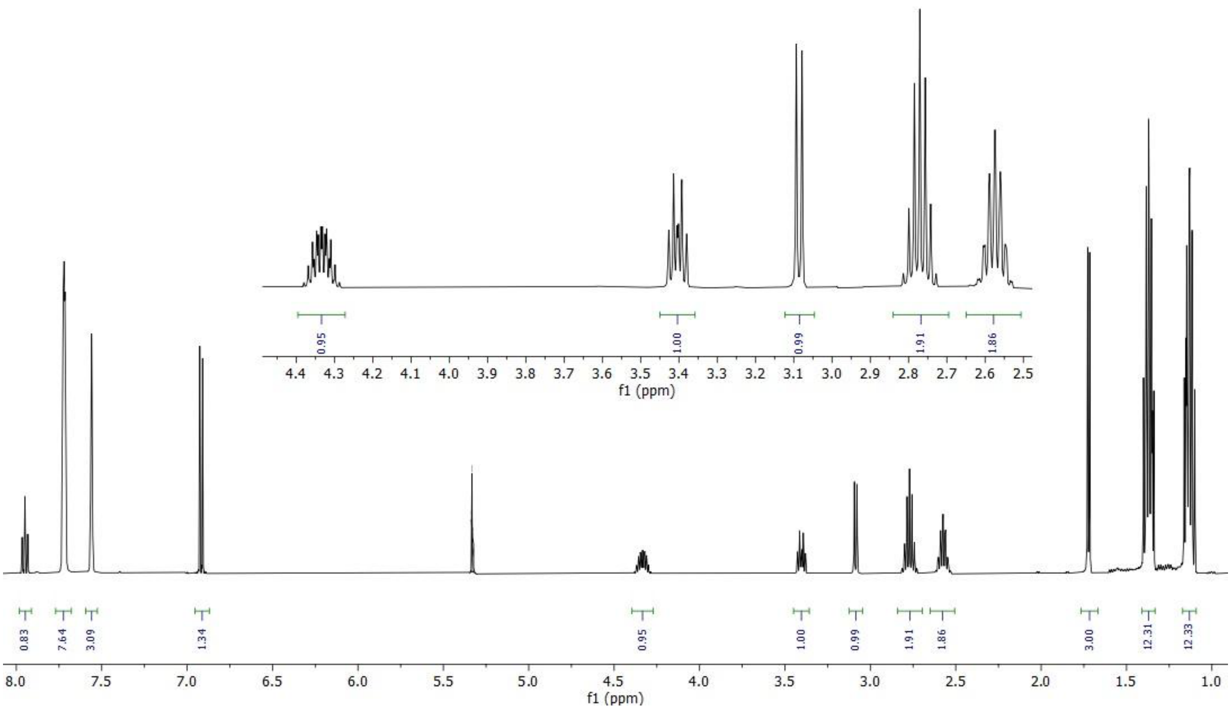

Figure S20 ^1H NMR spectrum of **6** (500 MHz, 298 K, CD_2Cl_2).

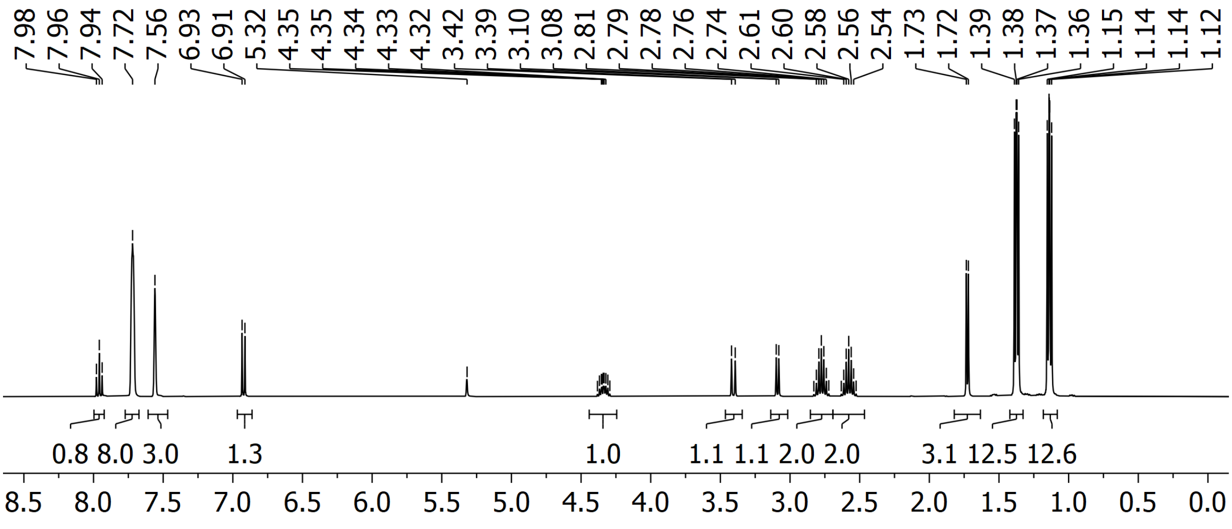

Figure S21 $^1H\{^{31}P\}$ NMR spectrum of **6** (400.12 MHz, 298 K, CD_2Cl_2).

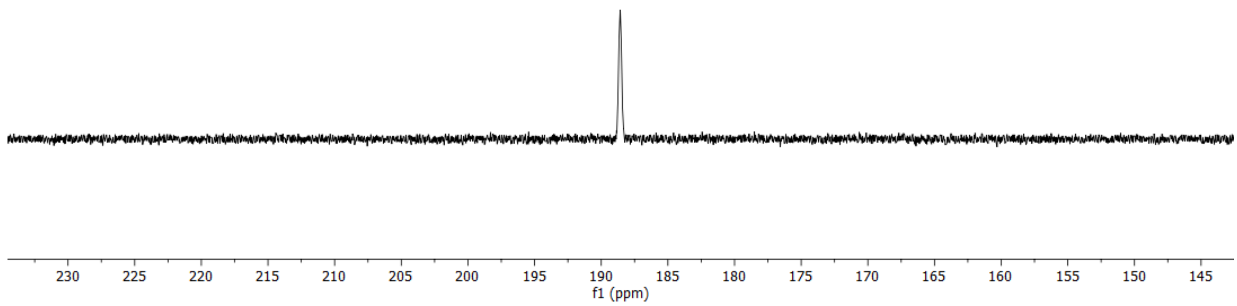

Figure S21 $^{31}P\{^1H\}$ NMR spectrum of **6** (203 MHz, 298 K, CD_2Cl_2).

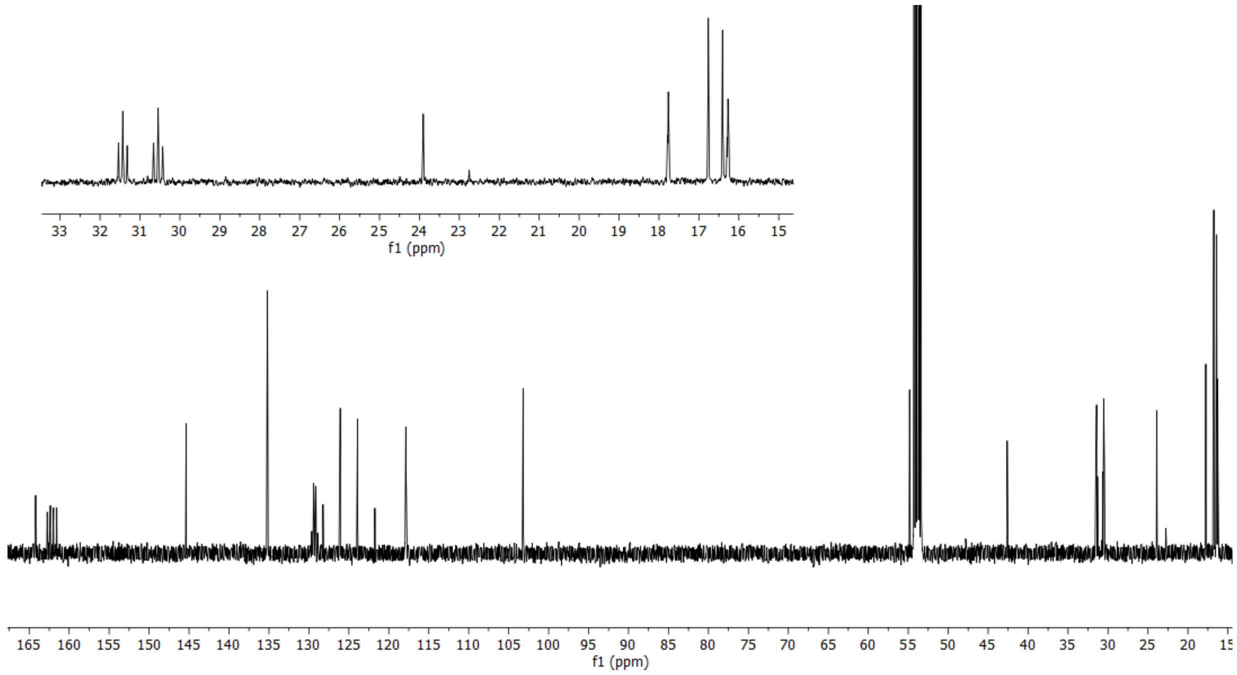

Figure S22 $^{13}C\{^1H\}$ NMR spectrum of **6** (126 MHz, 298 K, CD_2Cl_2).

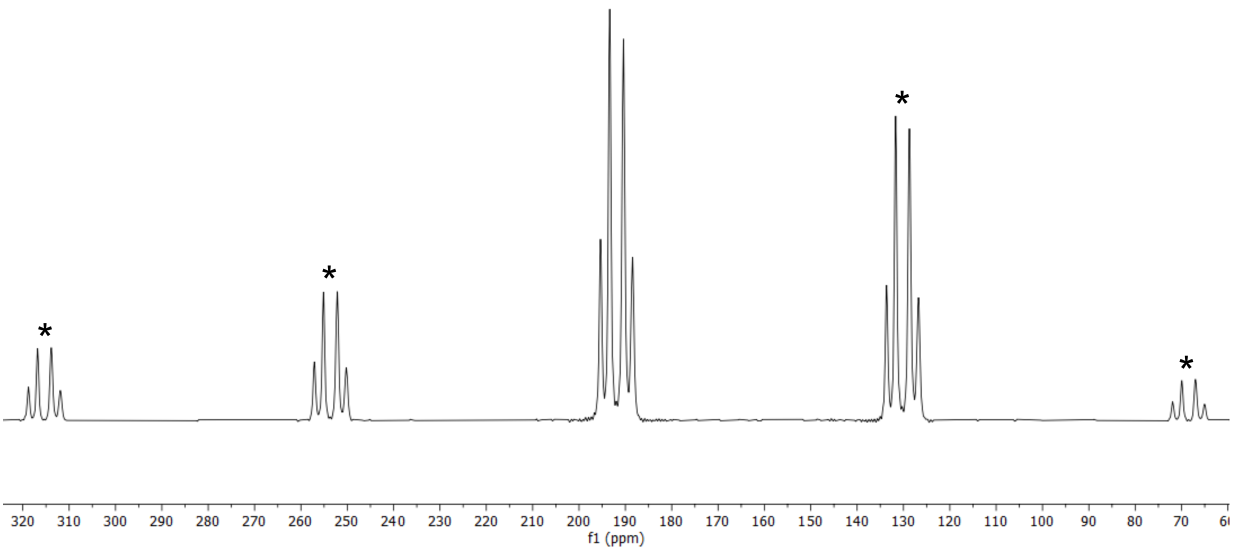

Figure S23 $^{31}P\{^1H\}$ SSNMR spectrum of **6** (162 MHz, 10 kHz spin rate, 298 K). * indicate spinning side-bands.

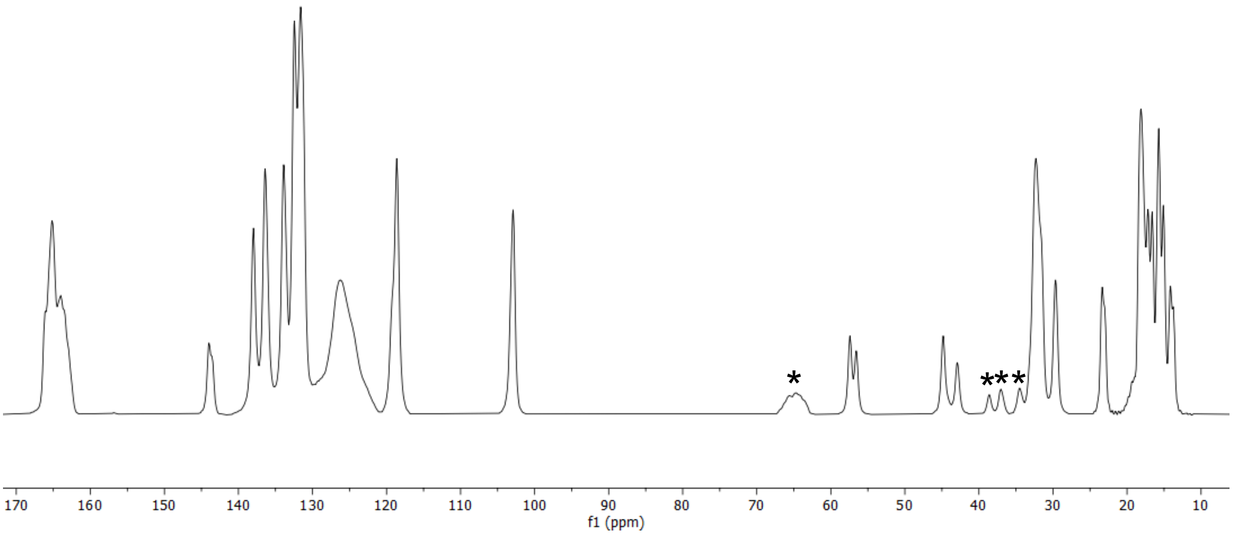

Figure S24 $^{13}C\{^1H\}$ SSNMR spectrum of **6** (101 MHz, 10 kHz spin rate, 298 K). * indicate spinning side-bands.

1.2.6. Synthesis of [(^{i}Pr-PONOP)IrH_2(η^2-propene)][BAr$^F{}_4$] (7)

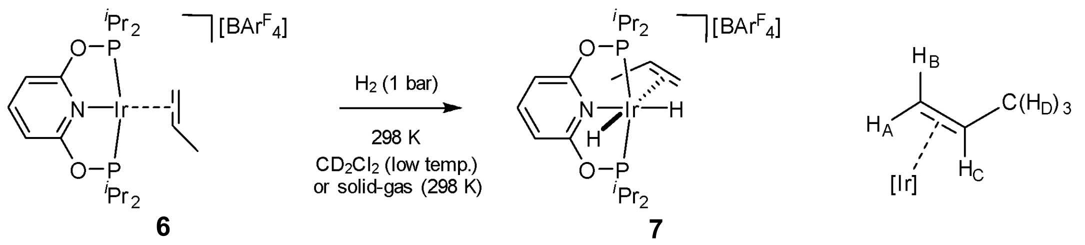

A solution of **6** (13.9 mg, 9.65 µmol) in CD_2Cl_2 (*ca*. 0.6 mL) treated with H_2 (4 bar absolute) in the absence of propene afforded on mixing an approximately equimolar mixture of **7** and **2** and propene, which is then observed to convert to exclusively **2** over the period of approximately 3 days. Note that attempts to obtain a solid-state structure of **7** *via* recrystallisation of CH_2Cl_2 solutions were not successful.

Propene-dihydride **7** was also obtained *via* a solid-gas route, monitored by SSNMR spectroscopy. A 4.0 mm zirconia rotor was packed with ~25 mg of propene complex **6** (~17 µmol). Left unsealed, the rotor was placed under H_2 (2 bar absolute) for 15 min before the system was gently purged with argon and the rotor was capped promptly inside an argon-filled glovebox. The rotor was then uncapped and exposed to dynamic vacuum for two hours, regenerating **6** as measured by ^{31}P{^{1}H} SSNMR. Exposure to H_2 (2 bar absolute) *in situ* in an uncapped rotor for a further five days resulted in no change.

^{1}H NMR (600 MHz, CD_2Cl_2, 298 K) *δ:* 7.76 (tt, $^3J_{HH}$ = 8.0 Hz, $^5J_{HP}$ = 1.0 Hz 1 H; heterocyclic C4), 7.72 (br. m, 8 H; [BAr$^F{}_4$]$^-$ *o*-H), 7.56 (s, 4 H; [BAr$^F{}_4$]$^-$ *p*-H), 6.83 (d, $^3J_{HH}$ = 7.0 Hz, 2 H; heterocyclic C3,5), 4.84 (br. s, fwhm = 40 Hz, 1 H; H_C), 3.56-3.68 (br., multiple resonances, 2 H; H_A + H_B), 2.73 (br. s, 2 H; ^{i}Pr CH), 2.63 (br. s, 2 H; ^{i}Pr CH), 1.72 (d, $^3J_{HH}$ = 5.9 Hz, 3 H; H_D), 1.41-1.51 (br. m, 6 H; ^{i}Pr CH_3), 1.22-1.36 (br. m, 6 H; ^{i}Pr CH_3), 1.05-1.19 (br. m, 6 H; ^{i}Pr CH_3), 0.77-0.92 (br. m, 6 H; ^{i}Pr CH_3), −11.64 (t, $^2J_{HP}$ = 18.0 Hz, 1 H; Ir-*H*), −16.50 (t, $^2J_{HP}$ = 10.5f Hz, 1 H; Ir-*H*).

^{13}C{^{1}H} NMR (151 MHz, CD_2Cl_2, 298 K) *δ* 162.1 (q, $^1J_{CB}$ = 49.9 Hz (^{11}B); [BAr$^F{}_4$]$^-$ *i*-C_{aryl}), 160.3 (br. s; heterocyclic C2,6), 144.8 (s; heterocyclic C4), 135.2 (m; [BAr$^F{}_4$]$^-$ *o*-C_{aryl}), 129.2 (qq, $^2J_{CF}$ = 31.5 Hz, $^4J_{CF}$ = 3.0 Hz; [BAr$^F{}_4$]$^-$ C_{aryl}-CF_3), 124.9 (q, $^1J_{CF}$ = 272.6 Hz; [BAr$^F{}_4$]$^-$ CF_3), 117.9 (septet, $^4J_{CF}$ = 4.0 Hz; [BAr$^F{}_4$]$^-$ *p*-C_{aryl}), 103.9 (s; heterocyclic C3,5), 82.5 (s; alkene CH), 62.0 (s; alkene CH_2), 30.9 (br. s; ^{i}Pr CH), 28.5 (br. vt, $^1J_{CP}$ ~ 15 Hz; ^{i}Pr CH), 21.2 (s; alkene CH_3), 19.5 (br. s; ^{i}Pr CH_3), 17.2 (br. s; ^{i}Pr CH_3), 17.1 (br. s; ^{i}Pr CH_3), 15.3 (br. s; ^{i}Pr CH_3).

^{13}C{^{1}H} SSNMR (101 MHz, 10 kHz spin rate, 298 K) *δ*: 162-167 ([BAr$^F{}_4$]$^-$ *i*-C_{aryl}), 161.2 (heterocyclic C2,6), 144.3 (heterocyclic C4), 120.5–141.0 ([BAr$^F{}_4$]$^-$), 117.0−120.5 ([BAr$^F{}_4$]$^-$ *p*-C_{aryl}), 105.1 (heterocyclic C3,5), 104.5 (heterocyclic C3,5), 84.5 (alkene CH), 77.9 (alkene CH), 61.8 (alkene CH_2), 59.2 (alkene CH_2), 28.2-33.7 (^{i}Pr CH), 21.1 (alkene CH_3), 20.0 (alkene CH_3), 13.0-19.3 (^{i}Pr CH_3).

^{31}P{^{1}H} NMR (243 MHz, CD_2Cl_2, 298 K) *δ*: 170.0 (br. d, $^2J_{PP'}$ ~ 320 Hz), 168.2 (br. d, $^2J_{PP'}$ ~ 320 Hz).

^{31}P{^{1}H} NMR (243 MHz, CD_2Cl_2, 185 K) *δ*: 172.3 (br. d, $^2J_{PP'}$ ~ 300 Hz, ~⅙), 169.7 (br. d, $^2J_{PP'}$ ~ 300 Hz, ~⅙), 167.7 (br. s, FWHM ~ 80 Hz, ~⅔),

^{31}P{^{1}H} SSNMR (162 MHz, 10 kHz spin rate, 298 K) *δ* 169-178 (multiple signals).

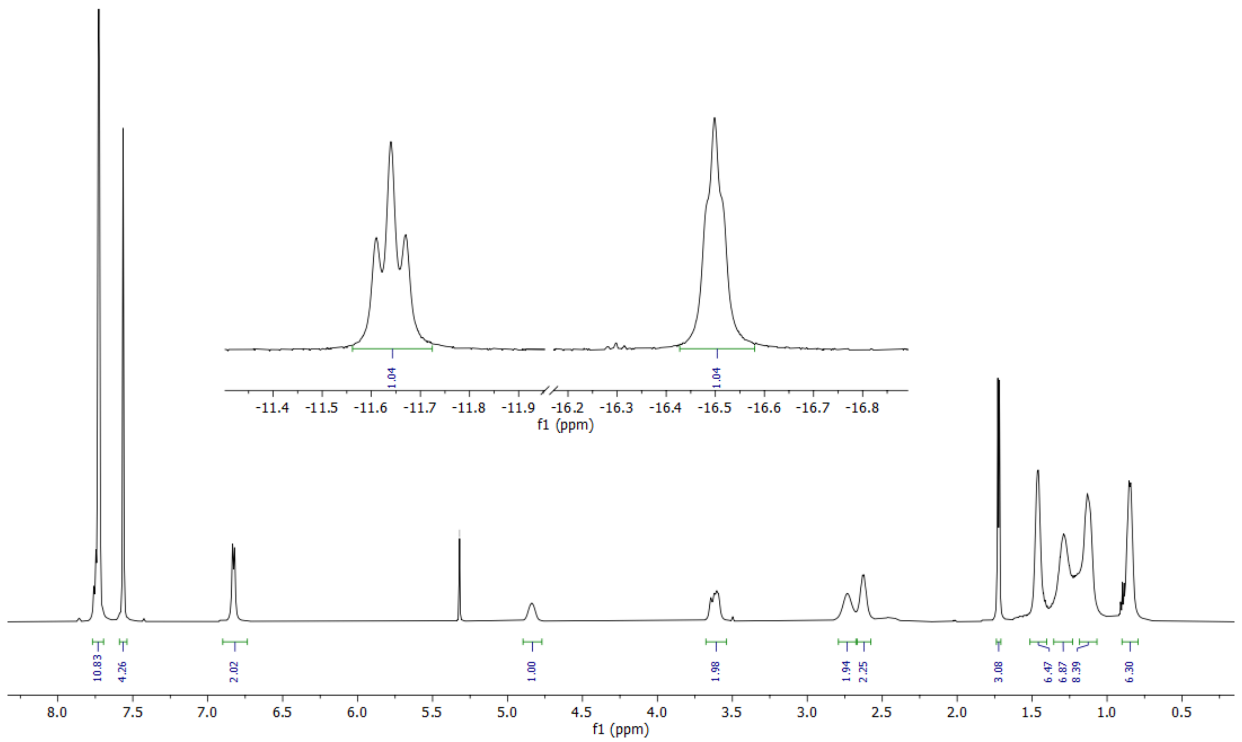

Figure S25 ^{1}H NMR spectrum of **7** (600 MHz, 298 K, CD_2Cl_2).

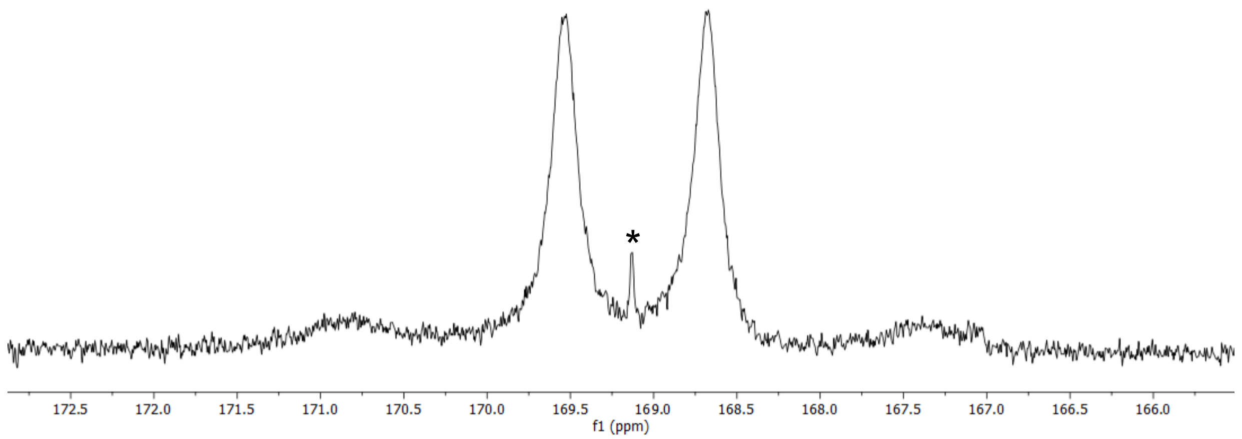

Figure S26 $^{31}P\{^{1}H\}$ NMR spectrum of **7** (243 MHz, 298 K, CD_2Cl_2). *indicates an unknown impurity.

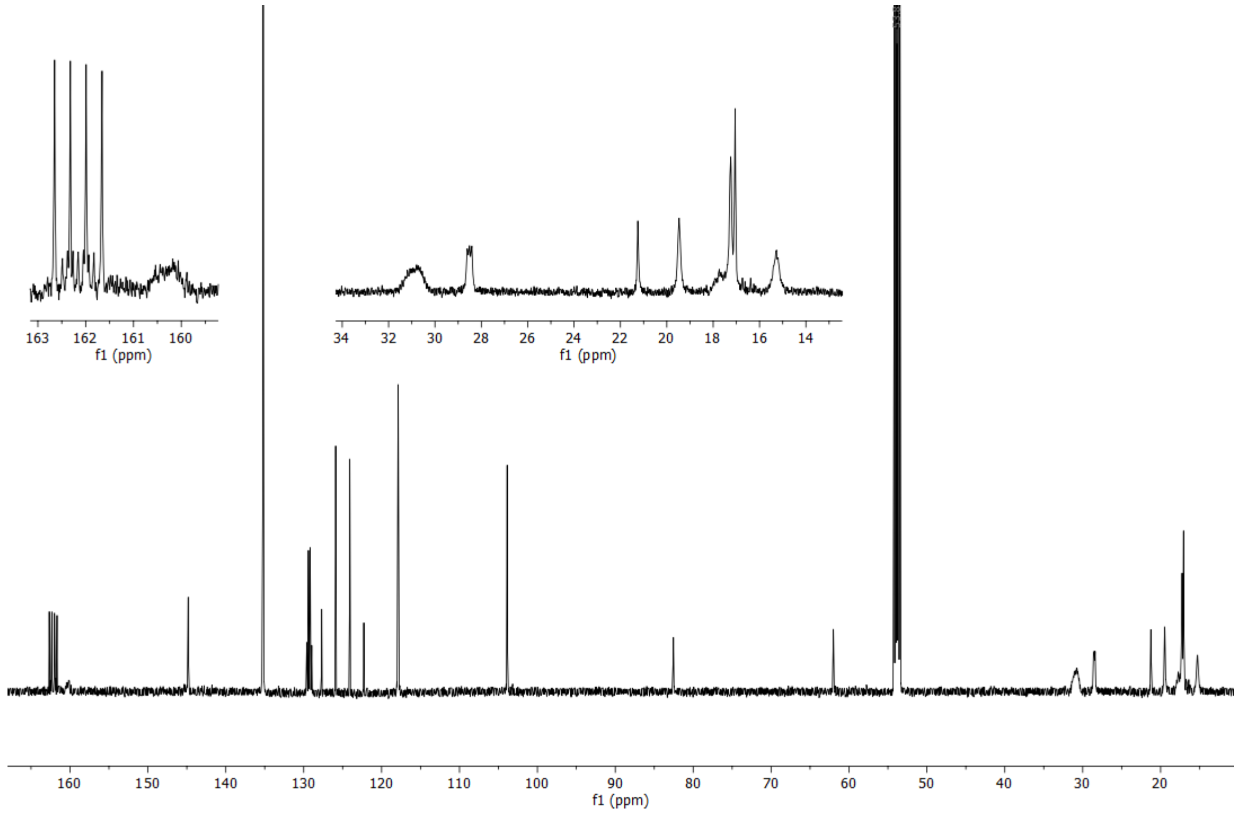

Figure S27 $^{13}C\{^1H\}$ NMR spectrum of **7** (151 MHz, 298 K, CD_2Cl_2).

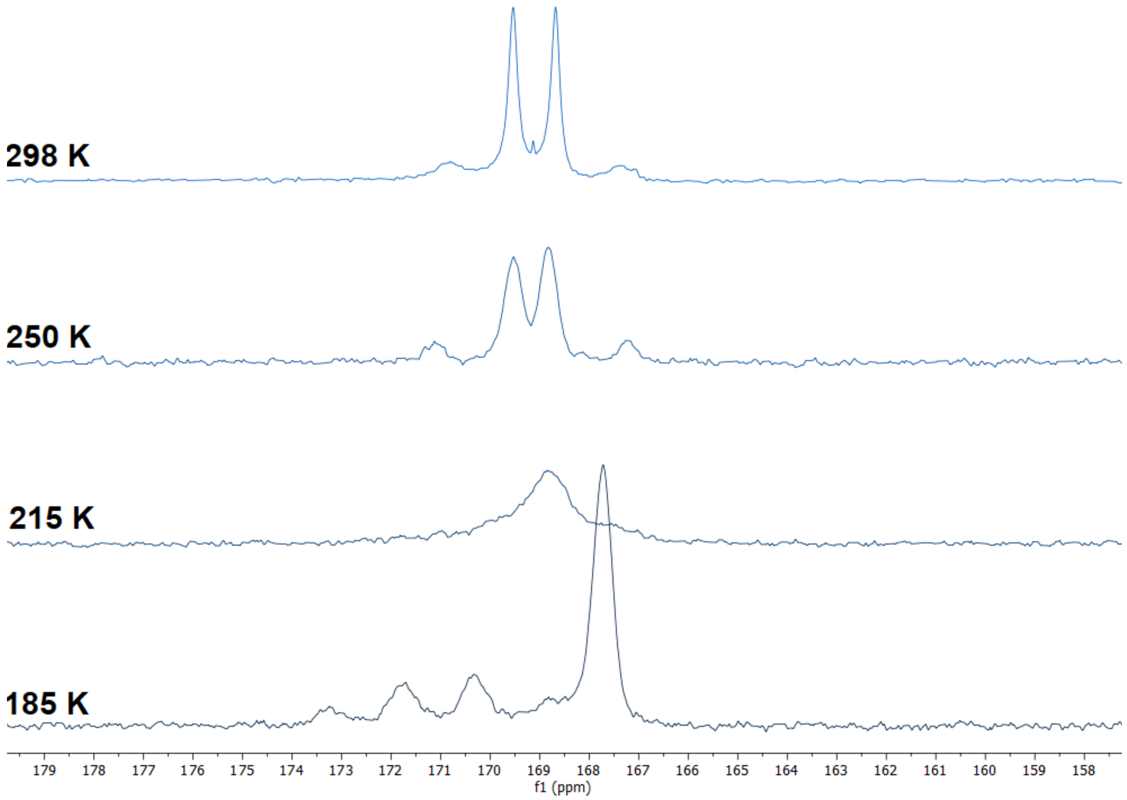

Figure 28 $^{31}P\{^1H\}$ VT-NMR spectra of **7** (203 MHz, CD_2Cl_2).

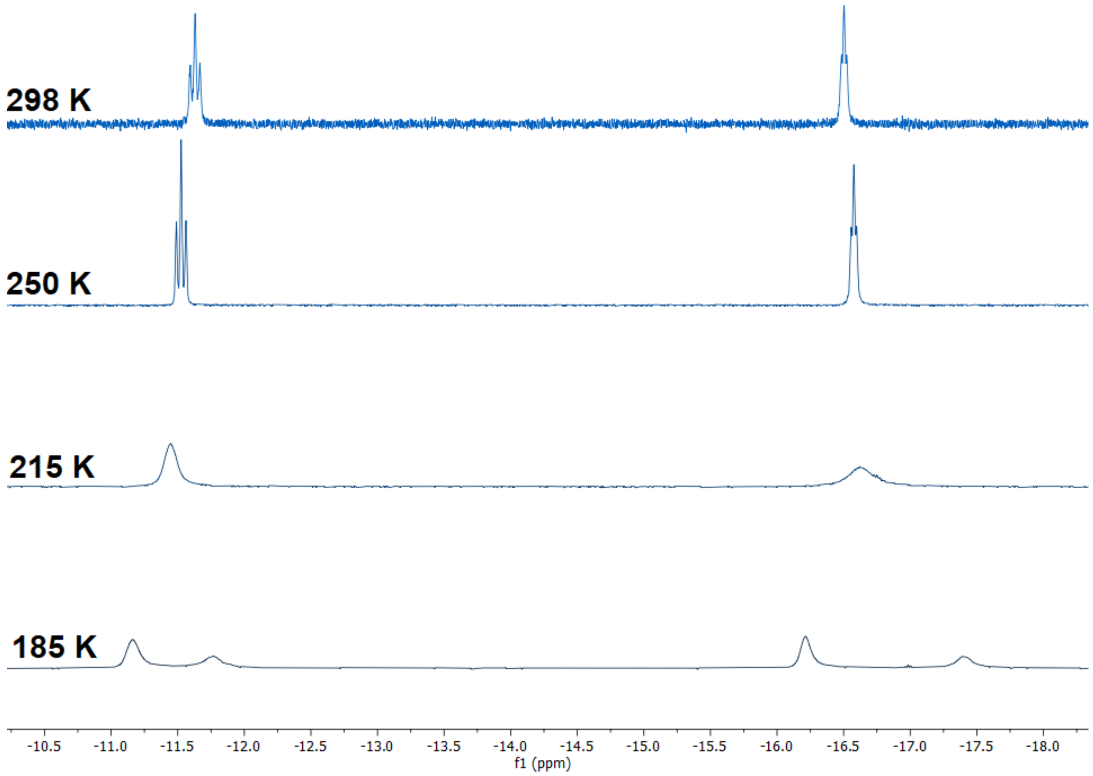

Figure S29 Hydridic region of the ^1H VT-NMR spectra of **7** (500 MHz, CD_2Cl_2).

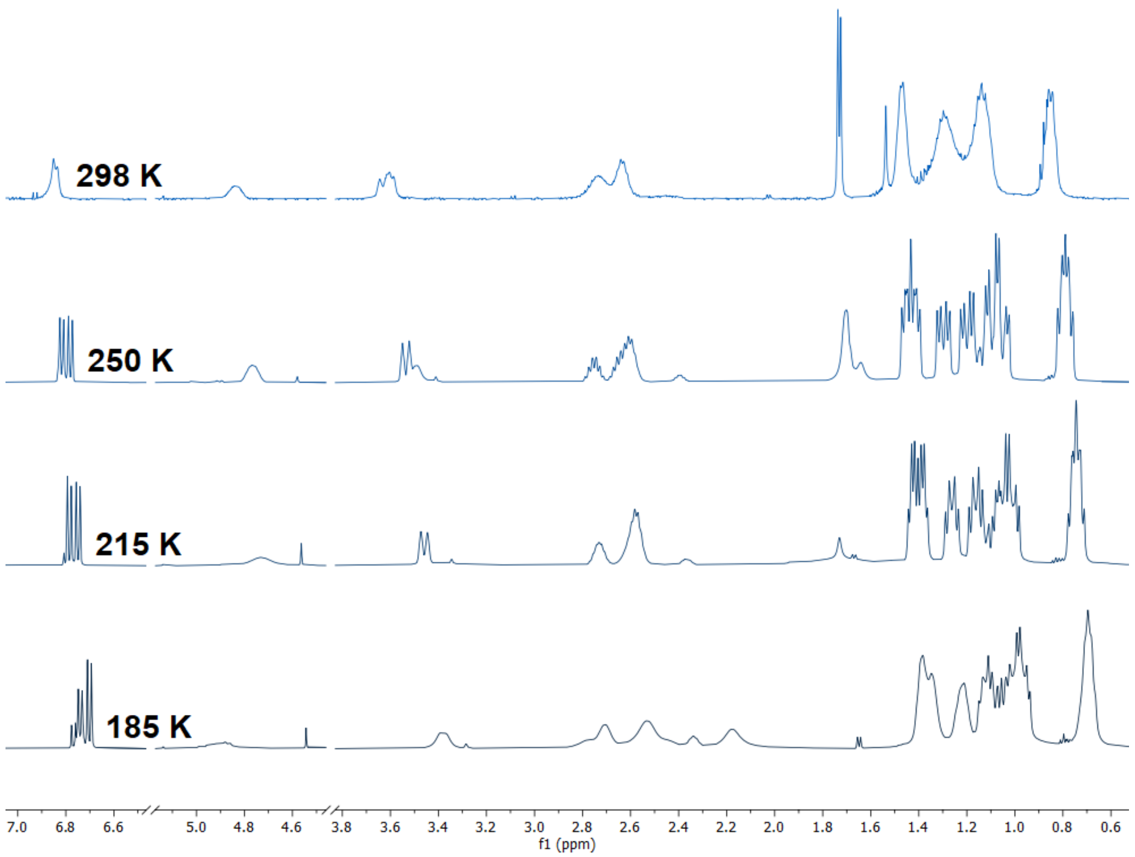

Figure 30 Aliphatic and aromatic regions of the ^{1}H VT-NMR spectra of **7** (500 MHz, CD_2Cl_2).

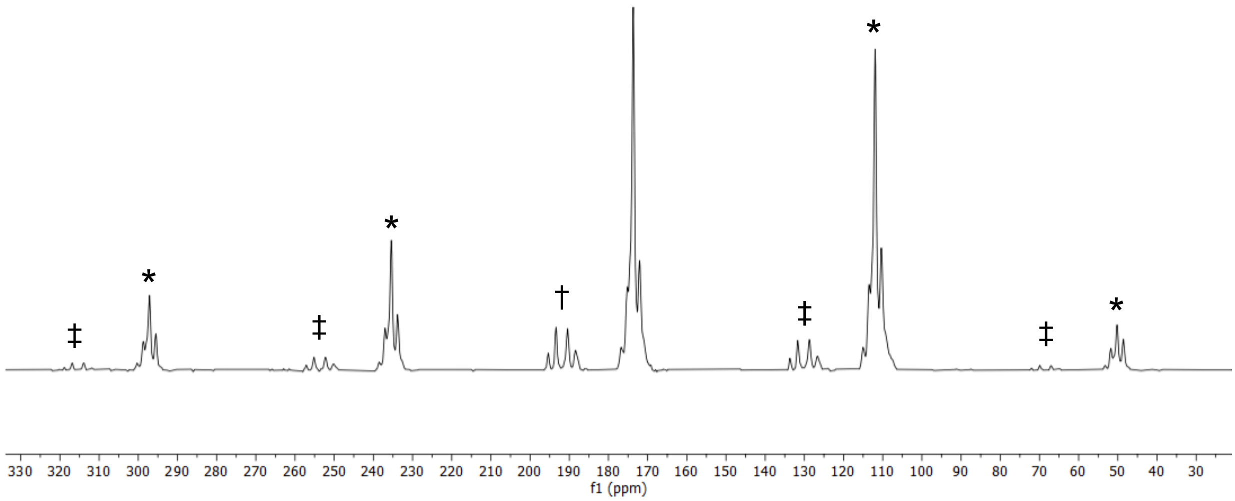

Figure S31 $^{31}P\{^{1}H\}$ SSNMR spectrum (162.03 MHz, 10 kHz spin rate, 298 K) of **7** (~90%) as generated by exposure of the packed rotor to H_2 (1.0 bar) for 15 minutes, with some loss of H_2 upon removal of the H_2 atmosphere to regenerate **6** (~10%). * indicate spinning side-bands; † indicate regenerated **6**, and ‡ indicate its corresponding side-bands.

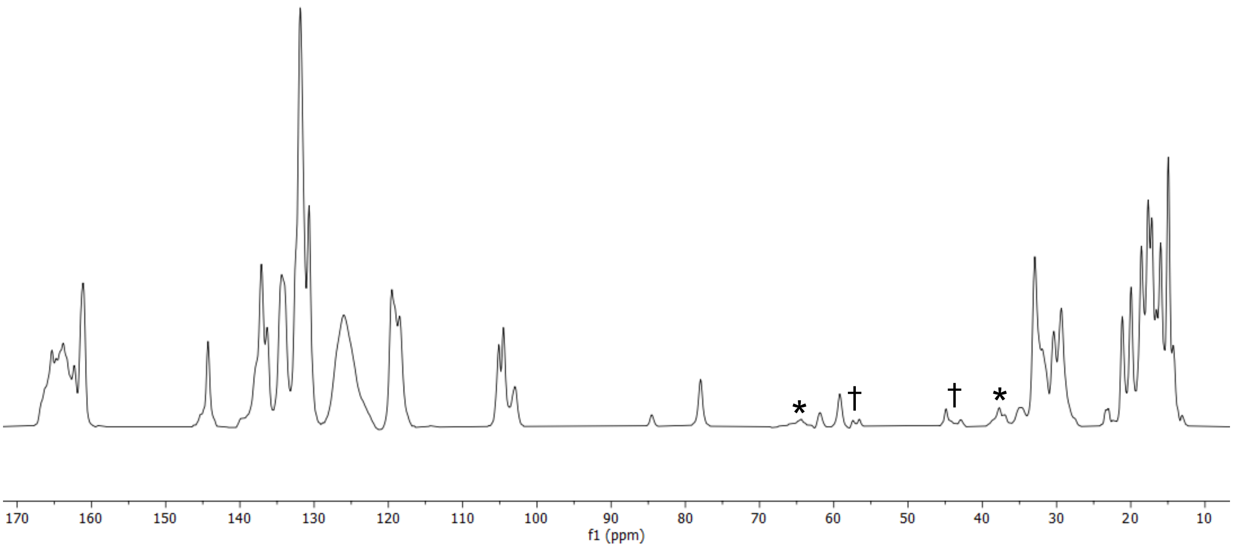

Figure S32 $^{13}C\{^{1}H\}$ SSNMR spectrum (101 MHz, 10 kHz spin rate, 298 K) of **7** (~90%) as generated by exposure of the packed rotor to H_2 (1.0 bar) for 15 minutes, with some loss of H_2 upon removal of the H_2 atmosphere to regenerate **6** (~10%). * indicate spinning side-bands; † indicate regenerated **6**.

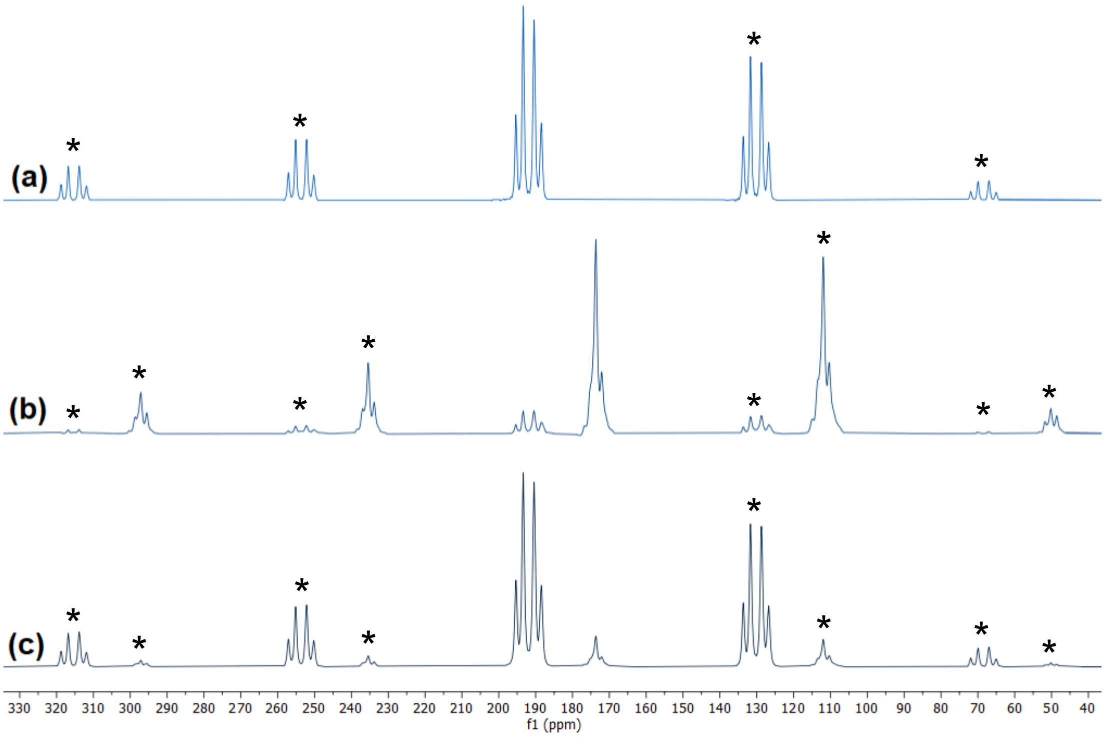

Figure S33 $^{31}P\{^{1}H\}$ SSNMR spectra (162.03 MHz, 10 kHz spin rate, 298 K) of (**a**) propene complex **6**; (**b**) propene-dihydride **7** (~90%) as generated by exposure of the packed rotor to H_2 (1.0 bar) for 15 minutes, with some loss of H_2 upon removal of the H_2 atmosphere to regenerate **6** (~10%), and (**c**) near-complete regeneration of **6** (~90%) by exposure of the rotor contents to dynamic vacuum for two hours. * indicate spinning side-bands.

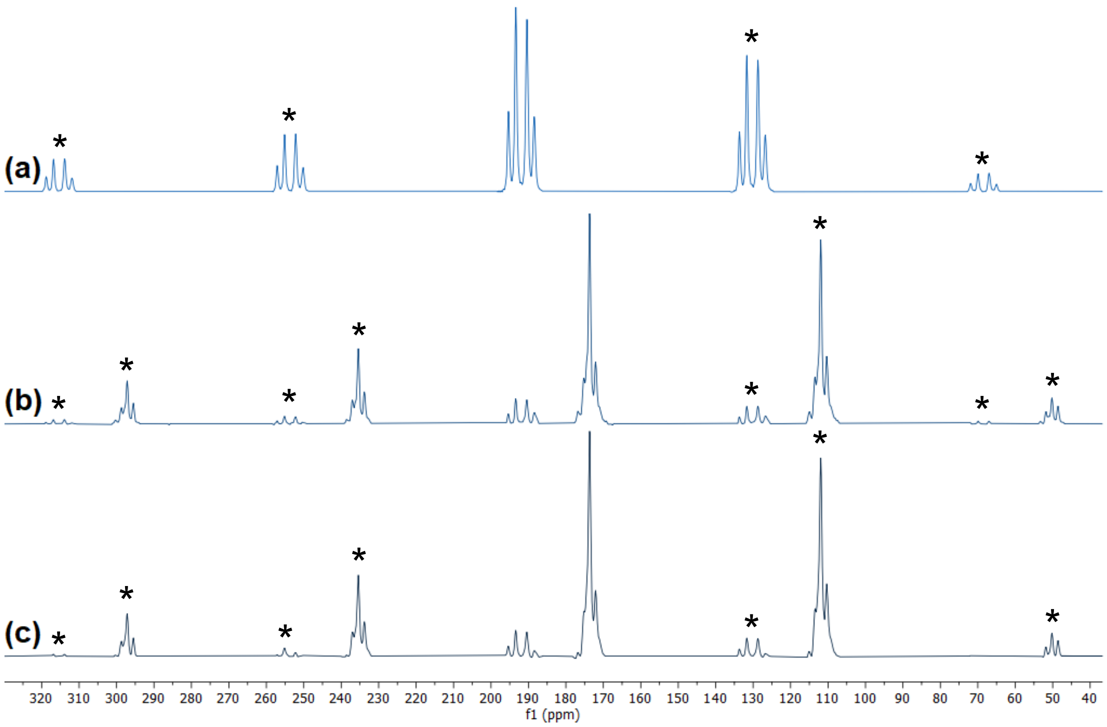

Figure S34 $^{31}P\{^1H\}$ SSNMR spectra (162.03 MHz, 10 kHz spin rate, 298 K) of (**a**) propene complex **6**; (**b**) propene-dihydride **7** (~90%) as generated by exposure of the packed rotor to H_2 (1.0 bar) for 15 minutes, with some loss of H_2 upon removal of the H_2 atmosphere to regenerate **6** (~10%), and (**c**) further exposure of the packed rotor to H_2 (1.0 bar) for five days, with no significant changes visible. * indicate spinning side-bands.

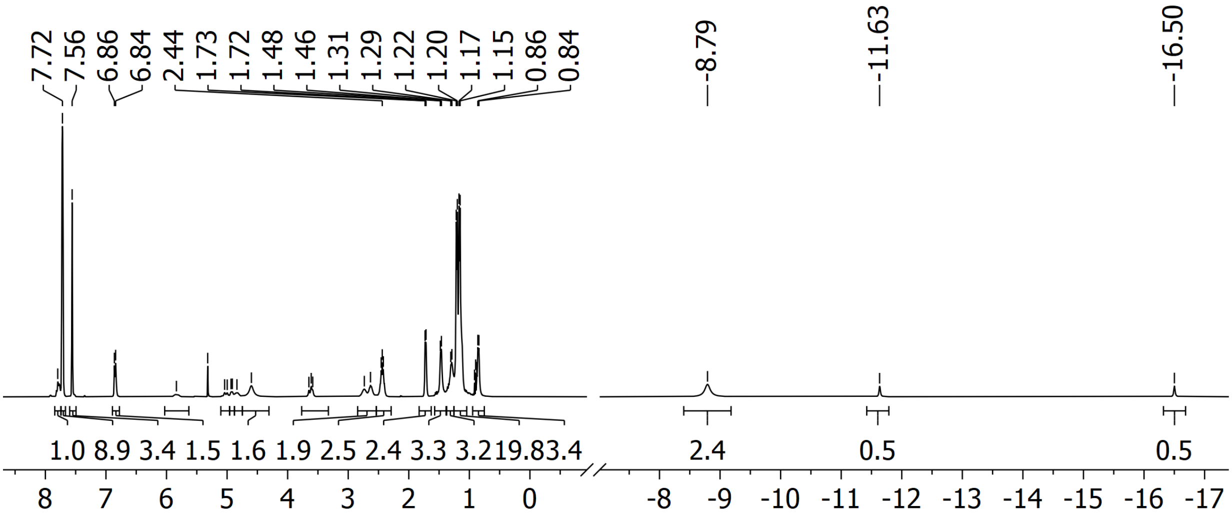

Figure S35 ^1H NMR spectrum (400.12 MHz, CD_2Cl_2) recorded after the time of mixing of a solution of **6** and H_2 (4 bar absolute)

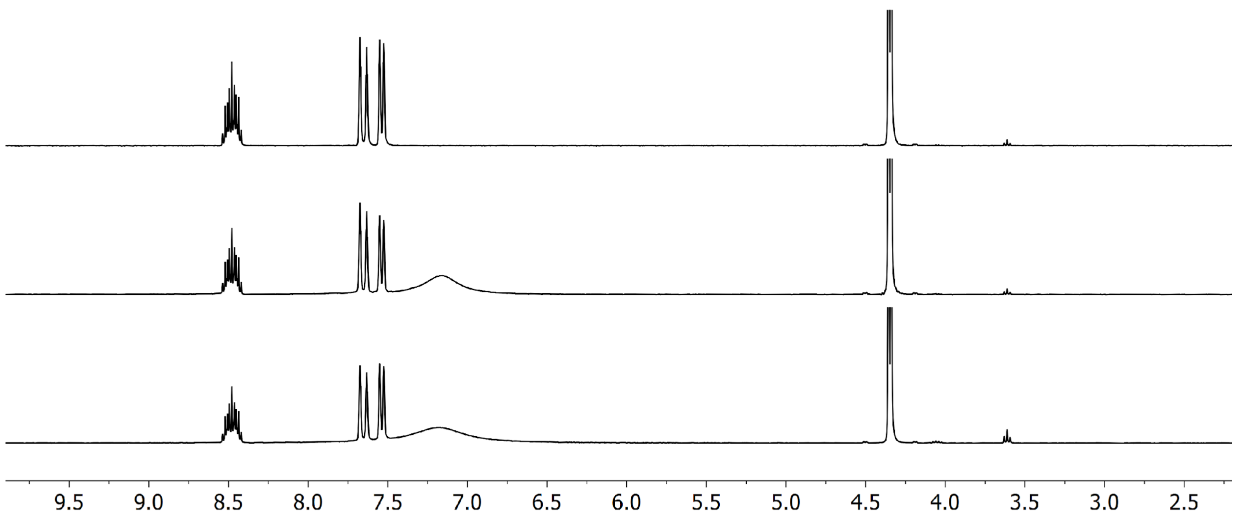

Figure S36 Gas phase ^{1}H NMR spectra of recorded of the headspace over a sample of **6** under exclusively propene (top), after addition of excess H_2 (middle) and after 18 hours (bottom). Spectra in the gas phase are not referenced.

1.3. Crystallography

XRD:

Single crystal X-ray diffraction data for all samples were collected as follows: a typical crystal was mounted on a MiTeGen Micromount using perfluoropolyether or polyisobutylene oil and cooled rapidly to the collection temperature in a stream of nitrogen gas using an Oxford Cryosystems Cryostream unit.[9] The structures were collected at the Department of Chemistry, University of York on an Oxford Diffraction SuperNova diffractometer using an EOS CCD camera.

Raw frame data were reduced using CrysAlisPro.[10] The structures were solved using *SHELXT*[11] and refined using full-matrix least squares refinement on all F2 data using *SHELXL*-2018[12] in the Olex2 GUI.[13] All hydrogen atoms were placed in calculated positions (riding model) with the exception of **5** where the hydride ligands were located against the difference map and freely refined isotropically. Disorder of the -CF_3 groups of all $[BAr^F{}_4]^-$ anions was treated by introducing a split site model and restraining geometries and displacement parameters appropriately.

In situ gas cell experiments were collected in the Experiments Hutch 2 (EH2) of Beamline I19, at the Diamond Light Source, using the Newport kappa-geometry four-circle diffractometer fitted with a Dectris Pilatus 300 K pixel-array photon-counting detector using an X-ray wavelength of 0.4859 A (Ag edge). Data sets consisted of three Ω sweeps with step size and exposure time of 0.2° and 0.2 s, respectively. All datasets recorded on I19 were at 273K using an Oxford Cryosystems - CryostreamPlus 700 device.

Under an inert atmosphere within a glove box equipped with a microscope, a crystal (70 x 70 x 50 microns) of complex **6** was selected without the use of manipulation oil and glued using a minimum amount of epoxy resin onto a mitogen loop. The glue only covered the corner of the crystal to avoid blocking any crystal channels. The sample mount was then inserted into the I19 beamline gas cell which consists of a 1 mm quartz capillary, Swagelok connections, and miniature quick connect. The gas cell was mounted on the diffractometer and the connected to the gas rig apparatus (1 bar N_2 atmosphere) using the swagelok miniature quick connector. The system was then slowly put under vacuum before an ambient dataset was obtained for comparison purposes. The system was then subjected to a 2 bar pure H_2 environment and an additional dataset was obtained yielding complex **7**. The diffraction pattern deteriorated upon H_2 with a drop of resolution from 0.65 A to 1.2 A and a smearing of diffraction peak shape.

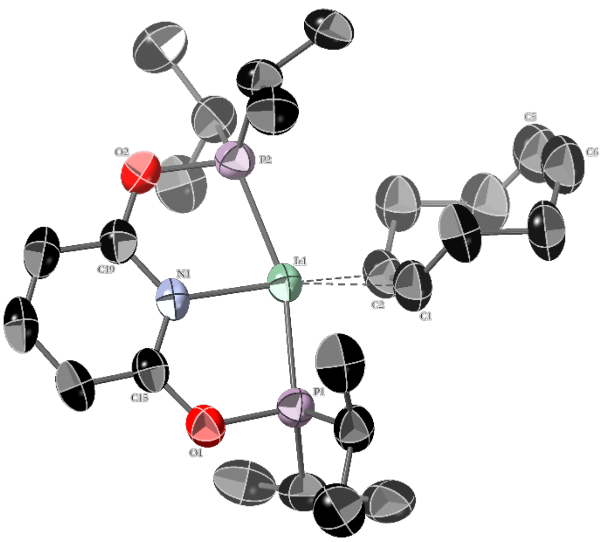

Figure S37 Molecular structures of **1**. Displacement ellipsoids at 50% probability. Selected key bond angles (°) and bond lengths (Å) for **1**: Ir1-N1, 2.055(3); Ir1-P1, 2.2556(8); Ir1-P2, 2.280(1); Ir1-C1, 2.181(4); Ir1-C2, 2.171(4); C1-C2, 1.359(6); C5-C6, 1.297(8); P1-Ir1-N1, 80.37(8); N1-Ir1-P2, 80.46(8).

All structures collected have been deposited in the Cambridge Crystallographic Data Centre with deposition numbers as specified below.

	1	5
**Empirical Formula**	$C_{57}H_{55}BF_{24}IrNO_2P_2$	$C_{52}H_{57}B_2F_{24}IrN_2O_2P_2$
**Formula Weight**	1506.97	1473.75
**Temperature/K**	150.0(2)	150.05(10)
**Crystal System**	Triclinic	Monoclinic
**Space group**	$P\overline{1}$	$P2_1/n$
**a/Å**	12.9568(2)	12.54330(10)
**b/Å**	13.0286(2)	26.5647(2)
**c/Å**	19.6838(3)	19.1920(2)
**α/°**	73.7910(10)	90
**β/°**	73.9640(10)	109.0180(10)
**γ/°**	83.3290(10)	90
**Volume/Å^3**	3063.82(8)	6045.89(10)
**Z**	2	4
**ρ_{calc} g/cm^3**	1.634	1.619
**μ/mm^{-1}**	5.751	5.813
**F(000)**	1496.0	2928.0
**Crystal size/mm^3**	0.1 × 0.1 × 0.05	0.1 × 0.05 × 0.01
**Radiation**	Cu Kα (λ = 1.54184)	Cu Kα (λ = 1.54184)
**2Θ range for data collection/°**	7.106 to 152.404	7.46 to 142.664
**Index ranges**	-16 ≤ h ≤ 16, -16 ≤ k ≤ 16, -24 ≤ l ≤ 24	-14 ≤ h ≤ 15, -32 ≤ k ≤ 32, -23 ≤ l ≤ 23
**Reflections collected**	86798	93686
**Independent reflections**	12726 [R_{int} = 0.0384, R_{sigma} = 0.0196]	11637 [R_{int} = 0.0443, R_{sigma} = 0.0225]
**Data/restraints/parameters**	12726/657/941	11637/390/881
**Goodness-of-fit on F^2**	1.051	1.031
**Final R indexes [I>=2σ (I)]**	R_1 = 0.0333, wR_2 = 0.0843	R_1 = 0.0260, wR_2 = 0.0599
**Final R indexes [all data]**	R_1 = 0.0362, wR_2 = 0.0869	R_1 = 0.0303, wR_2 = 0.0624
**Largest diff. peak/hole / e Å^{-3}**	1.51/-0.40	0.94/-1.28
**CCDC Deposition Number**	2175292	2175293

Table 1 Crystallographic Data for Structures Collected on Single Crystal Diffractometer.

	6	7
**Empirical Formula**	$C_{52}H_{43}BF_{24}IrNO_2P_2$	$C_{52}H_{48}BF_{24}IrNO_2P_2$
**Formula Weight**	1440.89	1439.86
**Temperature/K**	273(2)	273(2)
**Crystal System**	Triclinic	Triclinic
**Space group**	$P\overline{1}$	$P\overline{1}$
**a/Å**	12.2766(2)	12.585(3)
**b/Å**	12.5237(2)	12.978(3)
**c/Å**	20.1630(4)	19.526(3)
**α/°**	102.534(2)	104.537(16)
**β/°**	100.2280(10)	100.275(16)
**γ/°**	90.054(2)	90.322(18)
**Volume/Å^3**	2975.54(9)	3033.1(11)
**Z**	2	2
**ρ_{calc} g/cm^3**	1.608	1.577
**μ/mm^{-1}**	0.903	0.886
**F(000)**	1412	1422
**Crystal size/mm^3**	0.07 x 0.07 x 0.05	0.07 × 0.07 × 0.05
**Radiation**	Synchrotron (λ = 0.4859)	Synchrotron (λ = 0.4859)
**2Θ range for data collection/°**	2.878 to 43.896	2.98 to 23.36
**Index ranges**	$-18 \le h \le 18$, $-11 \le k \le 19$, $-30 \le l \le 30$	$-10 \le h \le 10$, $-10 \le k \le 10$, $-16 \le l \le 16$
**Reflections collected**	37719	11125
**Independent reflections**	21029 [R_{int} = 0.0433, R_{sigma} = 0.0527]	3604 [R_{int} = 0.0875, R_{sigma} = 0.0889]
**Data/restraints/parameters**	21029/142/945	3604/1057/936
**Goodness-of-fit on F^2**	0.971	1.159
**Final R indexes [I>=2σ (I)]**	R_1 = 0.0404, wR_2 =0.0961	R_1 = 0.0934, wR_2 = 0.2239
**Final R indexes [all data]**	R_1 = 0.0497, wR_2 = 0.1014	R_1 = 0.1131, wR_2 = 0.2360
**Largest diff. peak/hole / e Å^{-3}**	0.98/-.064	1.62/-1.19
**CCDC Deposition Number**	2175294	2175295

Table 2 Crystallographic Data for Structures Collected at I9 Beamline.

1.4. SEM Imaging

SEM images were taken using a JEOL JSM-7800F Prime Field Emission Scanning Electron Microscope at 1 keV using a lower electron detector (LED) at a working distance (WD) of 10 mm. Crystals for optical analysis were mounted onto aluminium stubs using carbon coated double-sided tape and carbon coated by vapor deposition prior to imaging.

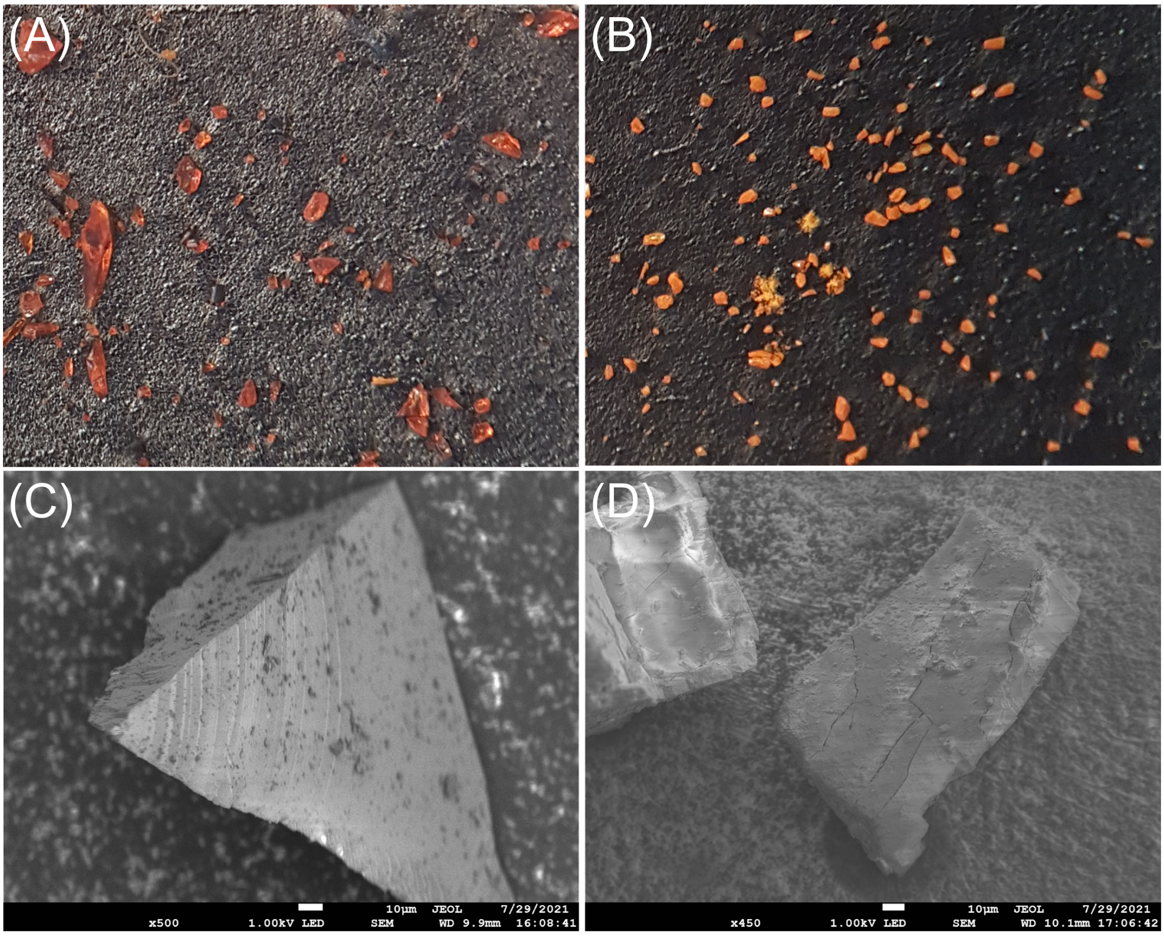

Figure S38 Optical (top) and SEM (bottom) images of samples of **6** (A, C) and a sample (B, D) of complex **6** that has undergone 5 × cycles of exposure to H_2 (2 bar absolute, 5 minutes) and then vacuum (*i.e.* **6** → **7** → **6**) showing cracking and loss of crystallinity after reaction.

2. Computational details

2.1. Solid-state calculations

All static Kohn-Sham DFT calculations were performed on periodic models of the studied iridium complexes, employing the Gaussian Plane Wave (GPW) formalism as implemented in the QUICKSTEP[14] module within the CP2K program suite (Version 5.0).[15] Molecularly optimized basis sets of double-ζ quality plus polarization in their short-range variant (DZVP-MOLOPT-SR-GTH)[16] were used on all atomic species. The interaction between the core electrons and the valence shell (Ir: 17, B: 3, C: 4, N: 5, O: 6, P: 5, F: 7, H: 1 electrons) was described by Goedecker-Teter-Hutter (GTH) pseudo potentials.[17-19] The generalized gradient approximation (GGA) to the exchange-correlation functional according to Perdew-Burke-Ernzerhof (PBE)[20] was used in combination with Grimme's D3-correction for dispersion interactions.[21] The auxiliary plane wave basis set was truncated at a cutoff of 500 Ry. The maximum force convergence criterion was set to 10^{-4} Eh·Bohr^{-1}, whilst default values were used for the remaining criteria. The convergence criterion for the self-consistent field (SCF) accuracy was set to 10^{-7} Eh and 10^{-8} Eh for geometry optimizations and vibrational analysis, respectively.

The Brillouin zone was sampled using the Γ-point. Initial coordinates for [Ir(^{i}Pr-PONOP)H_2(η^2-propene)][BAr$^F{}_4$], **6**, and [Ir(^{i}Pr-PONOP)H_2(η^2-propene)][BAr$^F{}_4$], **7**, were obtained from the experimental crystallographic data where in each case one of the disorder components was selected using Mercury.[22] Periodic boundary conditions (PBC) were applied throughout in combination with fixed unit cell parameters obtained from experiment.

Different reaction pathways were initially explored using an isolated iridium molecular cation model with the Gaussian suite of programs (see details below). Transition states located in this way provided the basis for transition state searches in the solid state, with pre-optimisations in the solid state run by fixing the key reacting atoms at one of the Ir-centres. A partial vibrational analysis was then used to identify the corresponding imaginary mode. This pre-optimized TS structure was then refined using the dimer method[23] with the tighter convergence criteria detailed above. For challenging fluxional processes the climbing image nudged elastic band (CI-NEB) method,[24] using 8 to 16 images, was used to obtain candidate transition states that were then optimised using the dimer method as above. All optimized stationary points were characterized by analysis of their numerical second derivatives with a displacement of 0.01 Bohr. Minima and transition states have no or exactly one imaginary eigenvalue, respectively. All transition states were further analysed displacing the transition state geometries along the negative mode in both directions and then fully optimising the two resulting structures. Further details on this protocol have been reported elsewhere.[25-27]

Geometries are supplied as a separate XYZ file along with the SCF energies. Gibbs free energies for structures computed in the solid state were calculated using the TAMkin software toolkit.[28]

2.2. Molecular calculations

DFT geometry optimizations were run with Gaussian 16 (Revision A.03)[29] using the BP86 functional.[30,31] Ir, and P centers were described with the Stuttgart RECPs and associated basis sets[32] and 6-31G** basis sets were used for all other atoms.[33,34] A set of d-orbital polarization functions was also added to P (ζ^d=0.387).[35] Stationary points were characterized with analytical frequency calculations. Transition states (one negative frequency) were characterized via IRC calculations and subsequent geometry optimizations to confirm the adjacent minima. Electronic energies were re-computed using the triple-ζ basis set Def2-TZVP[36,37] and include corrections for dispersion using the D3BJ method[38] and solvation in CH_2Cl_2 using PCM.[39] Geometries are supplied in the next section and as a separate XYZ file.

2.3. Additional free energy diagrams

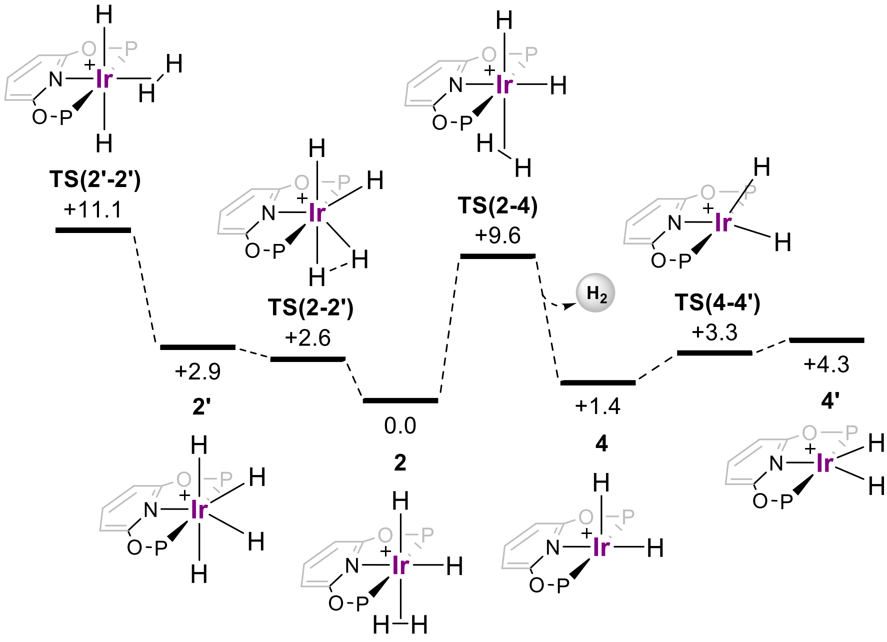

Figure S39 Computed free energy reaction profile (kcal/mol) for the isomerisation of **2**, **4** and related transition states in solution.

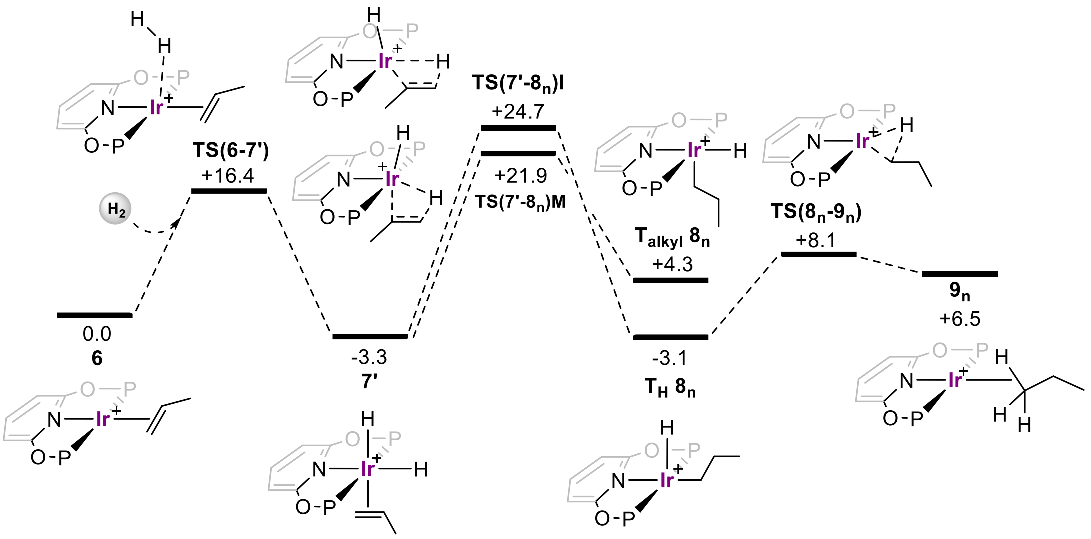

Figure S40 Computed free energy reaction profile (kcal/mol) for propene hydrogenation from **6** via n-propyl intermediate in solution.

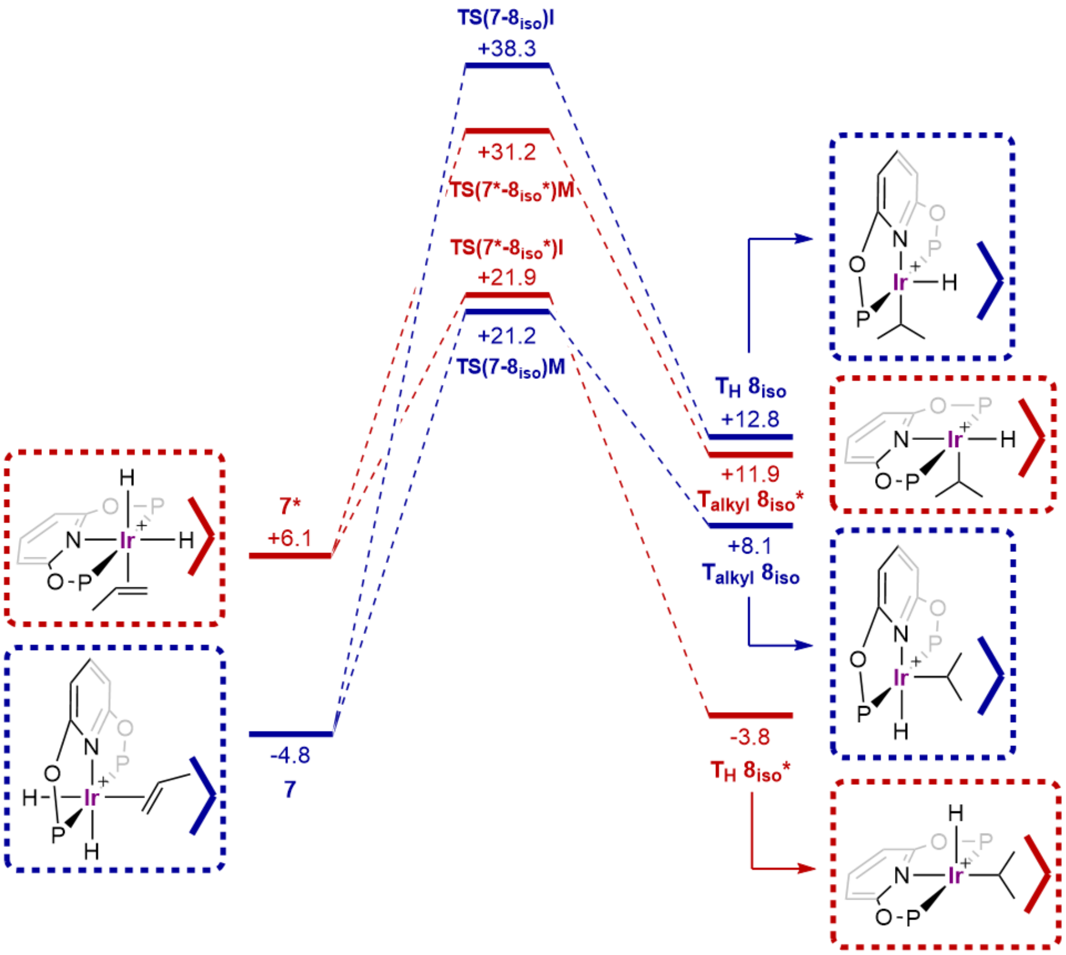

Figure S41 Computed free energy reaction profile (kcal/mol) for the hydride migration (M) and propene insertion (I) from **7** and **7*** in the “red” and “blue” lattices.

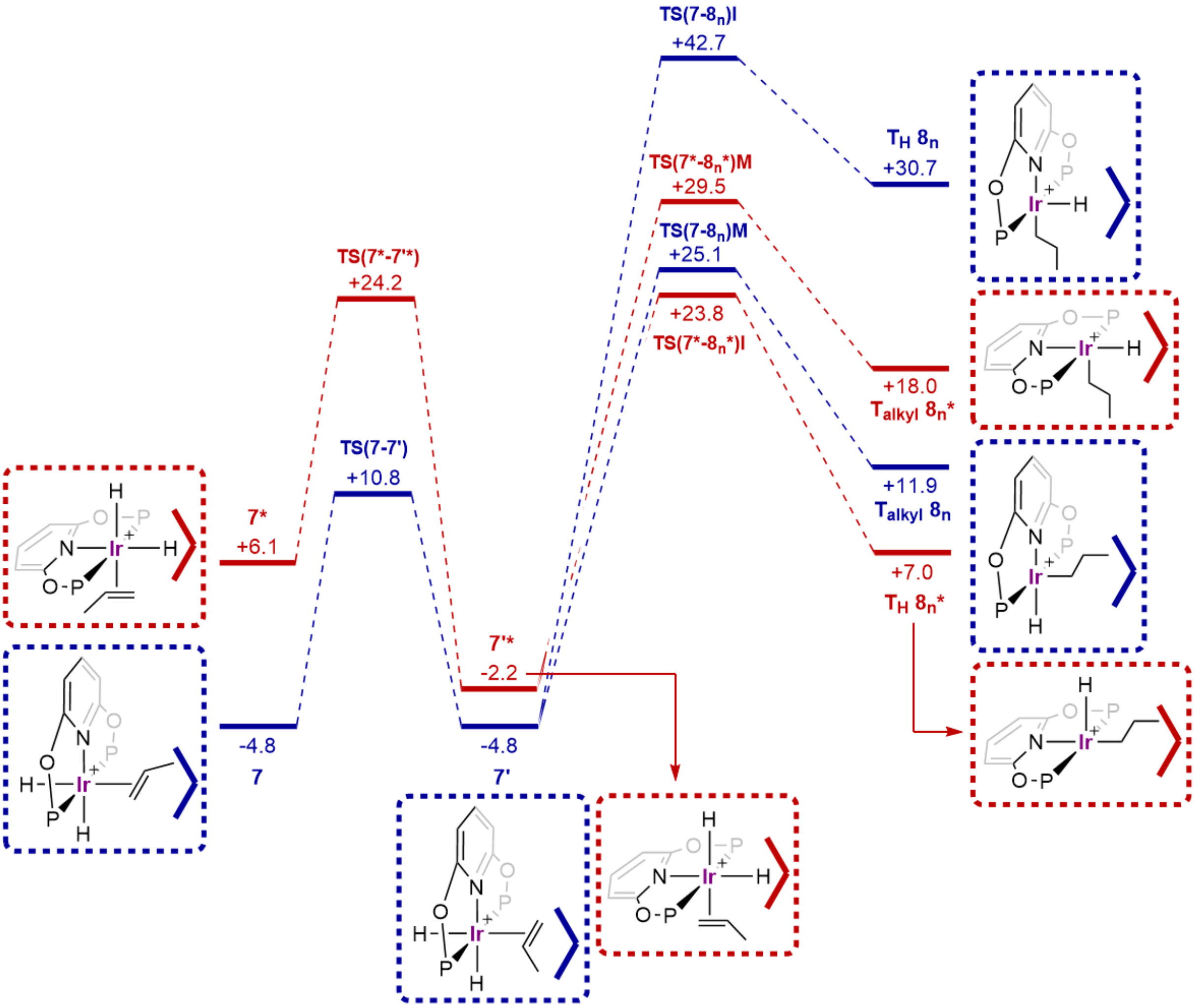

Figure S42 Computed free energy reaction profile (kcal/mol) for the rotation of propene in **7** and **7*** and the hydride migration (M) and propene insertion (I) from **7’** and **7’*** in the “red” and “blue” lattices.

3. References

(1) Martínez-Martínez, A. J.; Weller, A. S. Solvent-Free Anhydrous Li+, Na+ and K+ Salts of [B(3,5-(CF3)2C6H3)4]-, [BArF4]-. Improved Synthesis and Solid-State Structures. *Dalton Trans.* **2019**, *48* (11), 3551–3554. DOI: 10.1039/c9dt00235a.

(2) Crisenza, G. E. M.; McCreanor, N. G.; Bower, J. F. Branch-Selective, Iridium-Catalyzed Hydroarylation of Monosubstituted Alkenes via a Cooperative Destabilization Strategy. *J. Am. Chem. Soc.* **2014**, *136* (29), 10258–10261. DOI: 10.1021/ja505776m.

(3) Johnson, A.; Royle, C. G.; Brodie, C. N.; Martínez-Martínez, A. J.; Duckett, S. B.; Weller, A. S. H2-Alkene Complexes of [Rh(PONOP-IPr)(L)]+Cations (L = COD, NBD, Ethene). Intramolecular Alkene-Assisted Hydrogenation and Dihydrogen Complex [Rh(PONOP-IPr)(η-H2)]+. *Inorg. Chem.* **2021**, *60* (18), 13903–13912. DOI: 10.1021/acs.inorgchem.0c03687.

(4) Fulmer, G. R.; Miller, A. J. M.; Sherden, N. H.; Gottlieb, H. E.; Nudelman, A.; Stoltz, B. M.; Bercaw, J. E.; Goldberg, K. I. NMR Chemical Shifts of Trace Impurities: Common Laboratory Solvents, Organics, and Gases in Deuterated Solvents Relevant to the Organometallic Chemist. *Organometallics* **2010**, *29* (9), 2176–2179. DOI: 10.1021/om100106e.

(5) Morcombe, C. R.; Zilm, K. W. Chemical Shift Referencing in MAS Solid State NMR. *J. Magn. Reson.* **2003**, *162* (2), 479–486. DOI: 10.1016/S1090-7807(03)00082-X.

(6) Hu, B.; Gay, I. D. Probing Surface Acidity by 31 P Nuclear Magnetic Resonance Spectroscopy of Arylphosphines. *Langmuir* **1999**, *15* (2), 477–481. DOI: 10.1021/la980750a.

(7) Crabtree, R. H.; Hamilton, D. G. Classical (M = Os) and Nonclassical (M = Fe, Ru) Polyhydride Structures for the Complexes MH4(PR3)3. *J. Am. Chem. Soc.* **1986**, *108* (11), 3124–3125. DOI: 10.1021/ja00271a063.

(8) Luo, X. L.; Crabtree, R. H. Solution Equilibrium between Classical and Nonclassical Polyhydride Tautomers [ReH4(CO)L3]+ and [ReH2(H2-H2)(CO)L3]+ (L = PMe2Ph). Equilibrium Isotope Effects and an Intermediate Trihydrogen Complex in Intramolecular Site Exchange of Dihydrogen and Hydrid. *J. Am. Chem. Soc.* **1990**, *112* (19), 6912–6918. DOI: 10.1021/ja00175a027.

(9) Cosier, B. J.; Glazer, A. M. A Nitrogen-Gas-Stream Cryostat for General X-Ray Diffraction Studies. *J. Appl. Crystallogr.* **1986**, *19* (2), 105–107. DOI: 10.1107/S0021889886089835.

(10) Oxford Diffraction Ltd. **2011**.

(11) Sheldrick, G. M. A Short History of SHELX. *Acta Crystallogr. Sect. A Found. Crystallogr.* **2008**, *64* (1), 112–122. DOI: 10.1107/S0108767307043930.

(12) Sheldrick, G. M. SHELXT - Integrated Space-Group and Crystal-Structure Determination. *Acta Crystallogr. Sect. A Found. Crystallogr.* **2015**, *71* (1), 3–8. DOI: 10.1107/S2053273314026370.

(13) Dolomanov, O. V.; Bourhis, L. J.; Gildea, R. J.; Howard, J. A. K.; Puschmann, H. OLEX2 : A Complete Structure Solution, Refinement and Analysis Program. *J. Appl. Crystallogr.* **2009**, *42* (2), 339–341. DOI: 10.1107/S0021889808042726.

(14) VandeVondele, J.; Krack, M.; Mohamed, F.; Parrinello, M.; Chassaing, T.; Hutter, J., Quickstep: Fast and accurate density functional calculations using a mixed Gaussian and plane waves approach. *Comput. Phys. Commun.* **2005,** *167* (2), 103-128. DOI: 10.1016/j.cpc.2004.12.014

(15) Hutter, J.; Iannuzzi, M.; Schiffmann, F.; VandeVondele, J., cp2k: atomistic simulations of condensed matter systems. *Wiley Interdiscip. Rev. Comput. Mol. Sci.* **2013,** *4* (1), 15-25. DOI: 10.1002/wcms.1159

(16) VandeVondele, J.; Hutter, J., Gaussian basis sets for accurate calculations on molecular systems in gas and condensed phases. *J. Chem. Phys,* **2007,** *127* (11), 114105. DOI: 10.1063/1.2770708

(17) Hartwigsen, C.; Goedecker, S.; Hutter, J., Relativistic separable dual-space Gaussian pseudopotentials from H to Rn. *Phys. Rev. B* **1998,** *58* (7), 3641-3662. DOI: 10.1103/PhysRevB.58.3641

(18) Goedecker, S.; Teter, M.; Hutter, J., Separable dual-space Gaussian pseudopotentials. *Phys. Rev. B* **1996,** *54* (3), 1703-1710. DOI: 10.1103/PhysRevB.54.1703

(19) Krack, M., Pseudopotentials for H to Kr optimized for gradient-corrected exchange-correlation functionals. *Theor. Chem. Acc.* **2005,** *114* (1), 145-152. DOI: 10.1007/s00214-005-0655-y

(20) Perdew, J. P.; Burke, K.; Ernzerhof, M., Generalized Gradient Approximation Made Simple. *Phys. Rev. Lett.* **1996,** *77* (18), 3865-3868. DOI: 10.1103/PhysRevLett.77.3865

(21) Grimme, S.; Antony, J.; Ehrlich, S.; Krieg, H., A consistent and accurate ab initio parametrization of density functional dispersion correction (DFT-D) for the 94 elements H-Pu. *J. Chem. Phys,* **2010,** *132* (15), 154104. DOI: 10.1063/1.3382344

(22) Macrae, C. F.; Bruno, I. J.; Chisholm, J. A.; Edgington, P. R.; McCabe, P.; Pidcock, E.; Rodriguez-Monge, L.; Taylor, R.; van de Streek, J.; Wood, P. A., Mercury CSD 2.0 – New features for the visualization and investigation of crystal structures. *J. Appl. Crystallogr.* **2008,** *41*, 466-470. DOI: 10.1107/S0021889807067908

(23) Henkelman, G.; Jónsson, H., A dimer method for finding saddle points on high dimensional potential surfaces using only first derivatives. *J. Chem. Phys,* **1999,** *111*, 7010-7022. DOI: 10.1063/1.480097

(24) Henkelman, G.; Uberuaga, B. P.; Jónsson, H., A climbing image nudged elastic band method for finding saddle points and minimum energy paths. *J. Chem. Phys,* **2000,** *113*, 9901-9904. DOI: 10.1063/1.1329672

(25) Chadwick, F. M.; Kramer, T.; Gutmann, T.; Rees, N. H.; Thompson, A. L.; Edwards, A. J.; Buntkowsky, G.; Macgregor, S. A.; Weller, A. S., Selective C-H Activation at a Molecular Rhodium Sigma-Alkane Complex by Solid/Gas Single-Crystal to Single-Crystal H/D Exchange. *J. Am. Chem. Soc.* **2016,** *138* (40), 13369-13378. DOI: 10.1021/jacs.6b07968

(26) Brogaard, R. Y.; Weckhuysen, B. M.; Nørskov, J. K., Guest–host interactions of arenes in H-ZSM-5 and their impact on methanol-to-hydrocarbons deactivation processes. *J. Catal.* **2013,** *300*, 235-241. DOI: 10.1016/j.jcat.2013.01.009

(27) Piccini, G.; Sauer, J., Quantum Chemical Free Energies: Structure Optimization and Vibrational Frequencies in Normal Modes. *J. Chem. Theor. Comput.* **2013,** *9* (11), 5038-45. DOI: 10.1021/ct4005504

(28) Ghysels, A.; Verstraelen, T.; Hemelsoet, K.; Waroquier, M.; Van Speybroeck, V., TAMkin: A Versatile Package for Vibrational Analysis and Chemical Kinetics. *J. Chem. Inf. Model.* **2010,** *50* (9), 1736-1750. DOI: 10.1021/ci100099g

(29) Frisch, M. J.; Trucks, G. W.; Schlegel, H. B.; Scuseria, G. E.; Robb, M. A.; Cheeseman, J. R.; Scalmani, G.; Barone, V.; Petersson, G. A.; Nakatsuji, H.; Li, X.; Caricato, M.; Marenich, A. V.; Bloino, J.; Janesko, B. G.; Gomperts, R.; Mennucci, B.; Hratchian, H. P.; Ortiz, J. V.; Izmaylov, A. F.; Sonnenberg, J. L.; Williams-Young, D.; F. Ding, F. L.; F. Egidi, J. G.; B. Peng, A. P.; Henderson, T.; Ranasinghe, D.; Zakrzewski, V. G.; Gao, J.; Rega, N.; Zheng, G.; Liang, W.; Hada, M.; Ehara, M.; Toyota, K.; Fukuda, R.; Hasegawa, J.; Ishida, M.; Nakajima, T.; Honda, Y.; Kitao, O.; Nakai, H.; Vreven, T.; Throssell, K.; J. A. Montgomery, J.; Peralta, J. E.; Ogliaro, F.; Bearpark, M. J.; Heyd, J. J.; Brothers, E. N.; Kudin, K. N.; Staroverov, V. N.; Keith, T. A.; Kobayashi, R.; Normand, J.; Raghavachari, K.; Rendell, A. P.; Burant, J. C.; Iyengar, S. S.; Tomasi, J.; Cossi, M.; Millam, J. M.; Klene, M.; Adamo, C.; Cammi, R.; Ochterski, J. W.; Martin, R. L.; Morokuma, K.; Farkas, O.; Foresman, J. B.; Fox, D. J. *Gaussian 16, Revision A.03* Gaussian Inc.: Wallingford CT, 2016.

(30) Becke, A. D., Density-functional exchange-energy approximation with correct asymptotic behavior. *Phys. Rev. A* **1988,** *38* (6), 3098-3100. DOI: 10.1103/PhysRevA.38.3098

(31) Perdew, J. P., Density-functional approximation for the correlation energy of the inhomogeneous electron gas. *Phys. Rev. B* **1986,** *33* (12), 8822-8824. DOI: 10.1103/PhysRevB.33.8822

(32) Andrae, D.; Häußermann, U.; Dolg, M.; Stoll, H.; Preuß, H., Energy-adjustedab initio pseudopotentials for the second and third row transition elements. *Theor. Chim. Acta* **1990,** *77* (2), 123-141. DOI: 10.1007/BF01114537

(33) Hehre, W. J.; Ditchfield, R.; Pople, J. A., Self-Consistent Molecular Orbital Methods. XII. Further Extensions of Gaussian-Type Basis Sets for Use in Molecular Orbital Studies of Organic Molecules. *J. Chem. Phys,* **1972,** *56* (5), 2257-2261. DOI: 10.1063/1.1677527

(34) Hariharan, P. C.; Pople, J. A., The influence of polarization functions on molecular orbital hydrogenation energies. *Theor. Chim. Acta* **1973,** *28* (3), 213-222. DOI: 10.1007/BF00533485

(35) Höllwarth, A.; Böhme, M.; Dapprich, S.; Ehlers, A. W.; Gobbi, A.; Jonas, V.; Köhler, K. F.; Stegmann, R.; Veldkamp, A.; Frenking, G., A set of d-polarization functions for pseudo-potential basis sets of the main group elements Al-Bi and f-type polarization functions for Zn, Cd, Hg. *Chem. Phys. Lett.* **1993,** *208* (3), 237-240. DOI: 10.1016/0009-2614(93)89068-S

(36) Weigend, F.; Ahlrichs, R., Balanced basis sets of split valence, triple zeta valence and quadruple zeta valence quality for H to Rn: Design and assessment of accuracy. *Phys. Chem. Chem. Phys.* **2005,** *7* (18), 3297-3305. DOI: 10.1039/B508541A

(37) Weigend, F.; Köhn, A.; Hättig, C., Efficient use of the correlation consistent basis sets in resolution of the identity MP2 calculations. *J. Chem. Phys,* **2002,** *116* (8), 3175-3183. DOI: 10.1063/1.1445115

(38) Grimme, S.; Ehrlich, S.; Goerigk, L., Effect of the damping function in dispersion corrected density functional theory. *J. Comput. Chem.* **2011,** *32* (7), 1456-1465. DOI: 10.1002/jcc.21759

(39) Tomasi, J.; Mennucci, B.; Cammi, R., Quantum mechanical continuum solvation models. *Chem. Rev.* **2005,** *105* (8), 2999-3093. DOI: 10.1021/cr9904009
